# Global Identification of the Full-Length Transcripts and Alternative Splicing Related to Phenolic Acid Biosynthetic Genes in *Salvia miltiorrhiza*

**DOI:** 10.3389/fpls.2016.00100

**Published:** 2016-02-05

**Authors:** Zhichao Xu, Hongmei Luo, Aijia Ji, Xin Zhang, Jingyuan Song, Shilin Chen

**Affiliations:** ^1^Institute of Medicinal Plant Development – Chinese Academy of Medical Sciences, Peking Union Medical CollegeBeijing, China; ^2^Institute of Chinese Materia Medica – Chinese Academy of Chinese Medical ScienceBeijing, China

**Keywords:** *Salvia miltiorrhiza*, hybrid-seq, full-length transcripts, phenolic acid biosynthesis, alternative splicing, cytochrome P450s, laccases

## Abstract

Salvianolic acids are among the main bioactive components in *Salvia miltiorrhiza*, and their biosynthesis has attracted widespread interest. However, previous studies on the biosynthesis of phenolic acids using next-generation sequencing platforms are limited with regard to the assembly of full-length transcripts. Based on hybrid-seq (next-generation and single molecular real-time sequencing) of the *S. miltiorrhiza* root transcriptome, we experimentally identified 15 full-length transcripts and four alternative splicing events of enzyme-coding genes involved in the biosynthesis of rosmarinic acid. Moreover, we herein demonstrate that lithospermic acid B accumulates in the phloem and xylem of roots, in agreement with the expression patterns of the identified key genes related to rosmarinic acid biosynthesis. According to co-expression patterns, we predicted that six candidate cytochrome P450s and five candidate laccases participate in the salvianolic acid pathway. Our results provide a valuable resource for further investigation into the synthetic biology of phenolic acids in *S. miltiorrhiza*.

## Introduction

The alternative splicing events of mutiexon genes in multicellular eukaryotes can enhance the functional diversity of the encoded proteins and regulate gene expression through complex post-transcriptional mechanisms (Reddy et al., 2013). Recent alternative splicing analysis originating from next-generation sequencing (NGS, Illumina) has revealed that over 60% of multiexon genes undergo alternative splicing events in plants, such as *Arabidopsis thaliana* (Marquez et al., 2012), *Glycine max* (Shen et al., 2014), *Brachypodium* *distachyon* (Walters et al., 2013), and *Oryza sativa* (Zhang et al., 2010). However, the short-read assembly strategy of NGS limits its capacity to precisely quantify and predict alternative splicing events. In contrast, the long reads from SMRT sequencing (single molecule, real-time DNA sequencing using Pacific Biosciences RS II, PacBio) have demonstrated their advantage in sequencing full-length transcripts to identify and predict alternative splicing isoforms in human embryonic stem cells (Au et al., 2013; Roberts et al., 2013). Research has also addressed the disadvantage of high sequencing errors by correction with high-quality NGS reads (Au et al., 2012; Koren et al., 2012). Our recent study successfully demonstrated the localization of tanshinones to the root periderm and revealed the molecular mechanism of tanshinone biosynthesis using hybrid-seq (next-generation and single molecular real-time sequencing, NGS and TGS) of the root transcriptome of *Salvia miltiorrhiza* (Xu et al., 2015).

*Salvia miltiorrhiza* Bunge is one of the most commonly used medicinal plants in Traditional Chinese Medicine (TCM), as its dried root or rhizome is of great phytochemical value in the treatment of cardiovascular diseases and inflammation, and as an anti-oxidant, among other uses (Cheng, 2006; Wang et al., 2007; Dong et al., 2011). The main active components of *S. miltiorrhiza* are hydrophilic salvianolic acids (SAs), such as rosmarinic acid (RA) and lithospermic acid B (LAB; Wang et al., 2007), and lipophilic diterpenoid components, such as tanshinones I/IIA, dihydrotanshinone, and cryptotanshinone (Lei et al., 2014). The availability of the nuclear and chloroplast genomes (Qian et al., 2013) and transcriptome (Hua et al., 2011; Luo et al., 2014), along with research related to the molecular regulation (Zhang et al., 2013, 2015; Tan et al., 2014; Li et al., 2015) and biosynthesis of its bioactive components (Guo et al., 2013, 2015; Bloch and Schmidt-Dannert, 2014), strongly favors *S. miltiorrhiza* as a potential model medicinal plant for TCM research.

There are two pathways for RA synthesis, namely, the phenylpropanoid pathway and the tyrosine-derived pathway, and many of the key genes encoding enzymes in *S. miltiorrhiza* have been identified (Di et al., 2013; Hou et al., 2013; Bloch and Schmidt-Dannert, 2014). In the phenylpropanoid pathway, phenylalanine ammonia lyase (PAL), cinnamate 4-hydroxylase (C4H), and 4-coumarate-CoA ligase (4CL) sequentially catalyze the conversion of L-phenylalanine into 4-coumaroyl-CoA. In the tyrosine-derived pathway, tyrosine aminotransferase (TAT) and 4-hydroxyphenylpyruvate reductase (HPPR) sequentially catalyze the conversion of L-tyrosine into 4-hydroxyphenyllactic acid, which is then catalyzed into 3,4-dihydroxyphenllactic acid by an unknown CYP450 in *S. miltiorrhiza* (Di et al., 2013). Rosmarinic acid synthase (RAS) catalyzes conversion of the products from the two pathways to form 4-coumaroyl-3′, 4′-dihydroxyphenllactic acid (4C-DHPL), and SmCYP98A78 (allelic variant of SmCYP98A14, Chen et al., 2014) has been indicated as the specific hydroxylase that catalyzes the conversion of 4C-DHPL to RA (Di et al., 2013). Finally, oxidative dimerization of hydroxystilbene occurs, and laccase has been proposed to catalyze the oxidative reaction from RA to LAB (Giardina et al., 2010; Di et al., 2013). Although the phenolic acid biosynthetic pathway has in essence been proposed and identified, many homologous genes encoding key enzymes were uncovered by genome-wide strategy. Indeed, a total of 28 homologous genes of *SmPAL*, *SmC4H*, *Sm4CL*, *SmTAT*, *SmHPPR*, *SmRAS*, and *SmCYP98A78* have been identified by genome annotation (Wang et al., 2015).

In this study, using the hybrid-seq transcriptome of *S. miltiorrhiza* roots, we systematically analyzed the full-length transcripts and alternative splicing events of these 28 gene loci predicted as being related to RA biosynthesis. We then analyzed co-expression patterns and predicted candidate CYP450s and laccases related to the SA pathway. Our experiments not only reveal full-length transcript and alternative splicing data but also provide a reference tool for future studies on the genes involved in the biosynthesis of phenolic acids.

## Materials and Methods

### Plant Resources

*Salvia miltiorrhiza* (line 99-3) plants were cultivated at the Institute of Medicinal Plant Development (IMPLAD), Chinese Academy of Medical Sciences (CAMS) in an open experimental field. Roots, stems, and flowers were collected from 3-years-old plants growing in the field on May 27th, 2014. The roots were separated into three parts (periderm, phloem, and xylem) according to morphology and microstructure. Leaves with and without MeJA treatment (12 h, 200 μM; Sigma-Aldrich, St. Louis, MO, USA) were collected from tissue culture *S. miltiorrhiza* (line 99-3) plantlets at 25°C under long-day condition of 16-h light/8-h dark. All of the collected tissues originated from the same clone of *S. miltiorrhiza* (line 99-3).

### Transcriptomic Data

Single molecule real-time DNA sequencing data from pooled root tissues (periderm, phloem, and xylem) using the PacBio RS II platform (Pacific Biosciences of California, USA; Accession, SRX753381) and RNA-seq reads from different root tissues (periderm, phloem, and xylem) using the Illumina Hiseq 2500 platform (Illumina, USA) are reported in our recent study (Xu et al., 2015; Accession, SRR1640458). RNA-seq reads for different organs (root, stem, and flower) were generated using the Illumina HiSeq 2000 platform (Illumina, USA; Accession, SRP028388), and Illumina reads from leaves with and without 12 h MeJA treatment were obtained in a previous study (Luo et al., 2014; Accession, SRP051564).

### Bioinformatic Analysis

Single molecule real-time DNA sequencing data were corrected with Illumina short reads using LSC 1.alpha software (Au et al., 2012). Alternative splicing isoforms were analyzed using IDP 0.1.7 software, employing SMRT sequencing reads, Illumina short reads, and genome scaffolds (Au et al., 2013). Differential gene expression in various root tissues, organs and under MeJA treatment was analyzed using Tophat 2.0.12 and Cufflinks 2.2.1 (Trapnell et al., 2012) by mapping the Illumina short reads to *S. miltiorrhiza* genome sequences. Heat maps were constructed using R statistical project (Gentleman et al., 2004).

### Gene Structures and Phylogenetic Analysis

The alternative splicing isoforms found by IDP were viewed using the IGV 2.3.34 software (http://www.broadinstitute.org/software/igv/). The annotated gene sequences were corrected with the SMRT sequencing reads using Apollo software (Lee et al., 2013). Gene structures (e.g., intron, exon, intron phase) were also analyzed with Apollo. The full-length amino acid sequences of candidate CYP450s and laccases from *S. miltiorrhiza* and other species were aligned with MEGA 6 (Tamura et al., 2013). Neighbor-joining trees were then constructed using the bootstrap method with 1,000 replications.

### UPLC Analysis of LAB Content

The detection methods followed the Pharmacopeia of the People’s Republic of China. Periderm, phloem, and xylem samples were ground into powder (with three biological replicates for each sample), and each weighed sample of ground powder (0.2 g) was extracted with 50 mL of 75% methanol. After 1 h of heating reflux extraction, 75% methanol was added to complement and maintain a constant weight, and the sample was filtered through a 0.45-μm syringe filter. In addition, an LAB standard was dissolved with 75% methanol at a concentration of 140 mg/L. Chromatographic separation was performed using an ACQUITY UPLC BEH C18 column (2.1 mm × 100 mm, 1.7 μm) with a mobile phase of 30% methanol, 10% acetonitrile, 1% methanoic acid, and 59% H_2_O in a Waters UPLC system (Waters, USA). The detection wavelength was set to 286 nm.

### Gene Expression Analysis by qRT-PCR

Nine RNA samples were isolated from different *S. miltiorrhiza* tissues (periderm, phloem, xylem, root, stem, leaf, and flower), which were collected from experimental field, and leaves from tissue culture plantlets were treated with MeJA (control or 12-h MeJA treatment). Total RNA (three biological replicates for each sample) was isolated using the RNeasy Plus Mini kit (Qiagen, Germany). Reverse transcription was performed with PrimeScript^TM^ Reverse Transcriptase (TaKaRa, Japan). The qRT-PCR primers were designed with Primer Premier 6 (**Supplementary Table S1**), and their specificity was verified by PCR. The qRT-PCR analysis was performed in triplicate using SYBR^®^ Premix Ex Taq^TM^ II (TaKaRa, Japan), with *SmActin* as a reference gene, and a 7500 real-time PCR system (ABI, USA). The Ct value was calculated for analyzing relative expression levels using the 2^-ΔΔCT^ method (Livak and Schmittgen, 2001). To detect differences in the expression of candidate genes among various tissues, one-way ANOVA was performed using IBM SPSS 20 software (IBM Corporation, USA). *P* < 0.01 was considered highly significant. Gene co-expression analysis of candidate genes was performed using Pearson’s correlation test.

## Results

### Localization of SA Accumulation in *S. miltiorrhiza* Root

**FIGURE 1:**
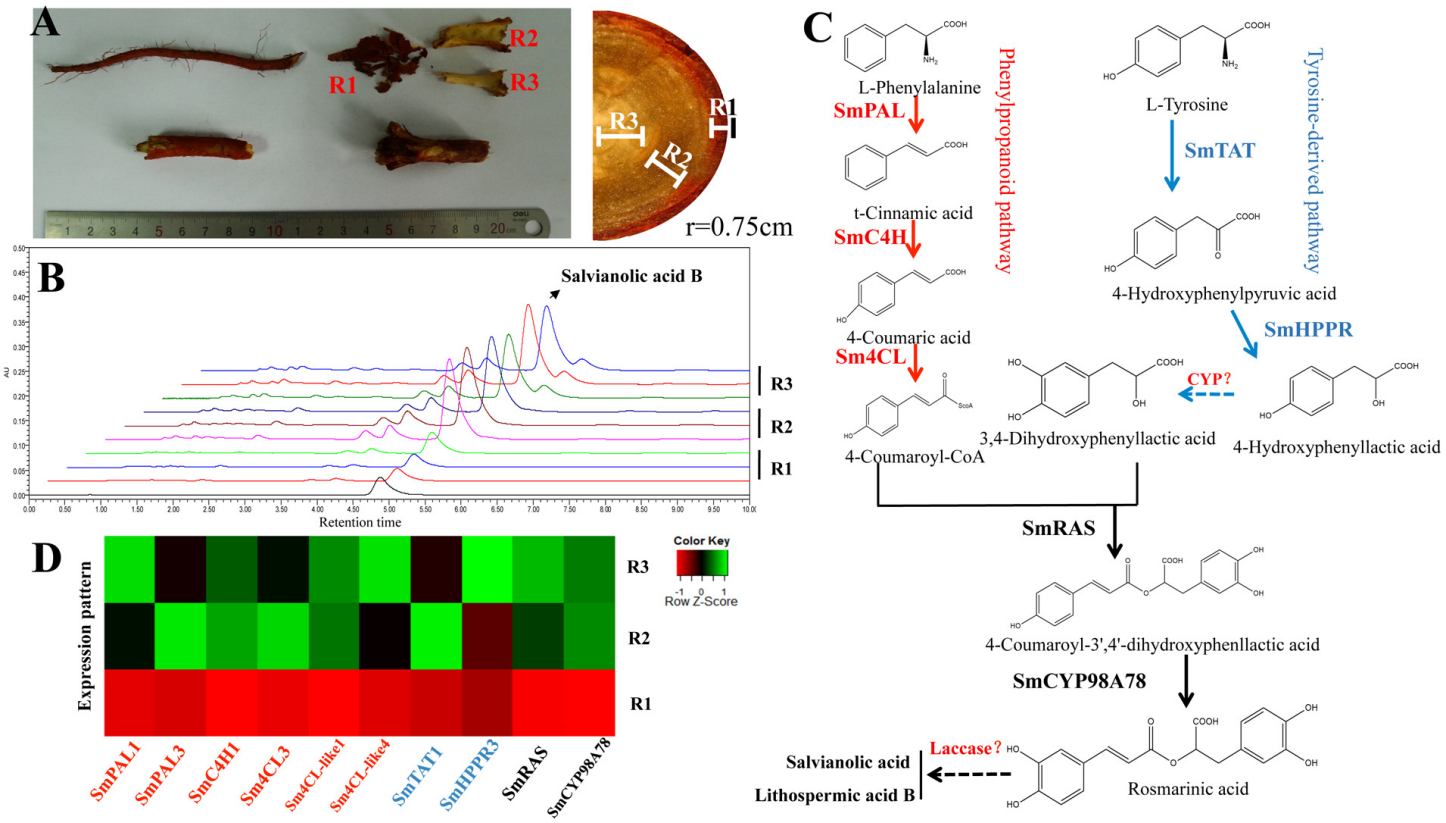
**The distribution of SA in different root tissues (periderm, phloem, and xylem) and the proposed biosynthetic pathway of SA in *Salvia miltiorrhiza*.** **(A)** The root of *S. miltiorrhiza*. R1: periderm; R2: phloem; R3: xylem. **(B)** Differences in LAB contents in the three different root tissues. **(C)** The proposed pathway related to SA biosynthesis. **(D)** Differentially expressed genes related to SA; their expression in different root tissues was consistent with the distribution of LAB.

The rhizome or root of *S. miltiorrhiza* is the primary medicinal part of this well-known herb. The hydrophilic phenolic acids in the *S. miltiorrhiza* root are mainly distributed in the phloem and xylem. UPLC identification demonstrated a similar LAB content in the phloem and xylem, which were five times higher than that in the periderm (**Figures 1A,B**). These results provided a potential basis for co-expression analysis of SA biosynthetic genes in the *S. miltiorrhiza* root.

### Isoform Detection and Prediction of RA Biosynthetic Genes

Using the next-generation sequencing platform (Illumina), RNA-seq data (a total of 867,864,885 reads) from *S. miltiorrhiza* periderm, phloem, xylem, root, stem, flower, leaf, and leaf after 12 h of MeJA treatment were collected. Using SMRT sequencing (PacBio RS II platform), full-length cDNA libraries from pooled periderm, phloem, and xylem samples were performed for a long-read survey, and 796,011 subreads were employed to identify full-length transcripts and alternative splicing events by hybrid-seq using the IDP (isoforms detection and prediction) pipeline.

**FIGURE 2:**
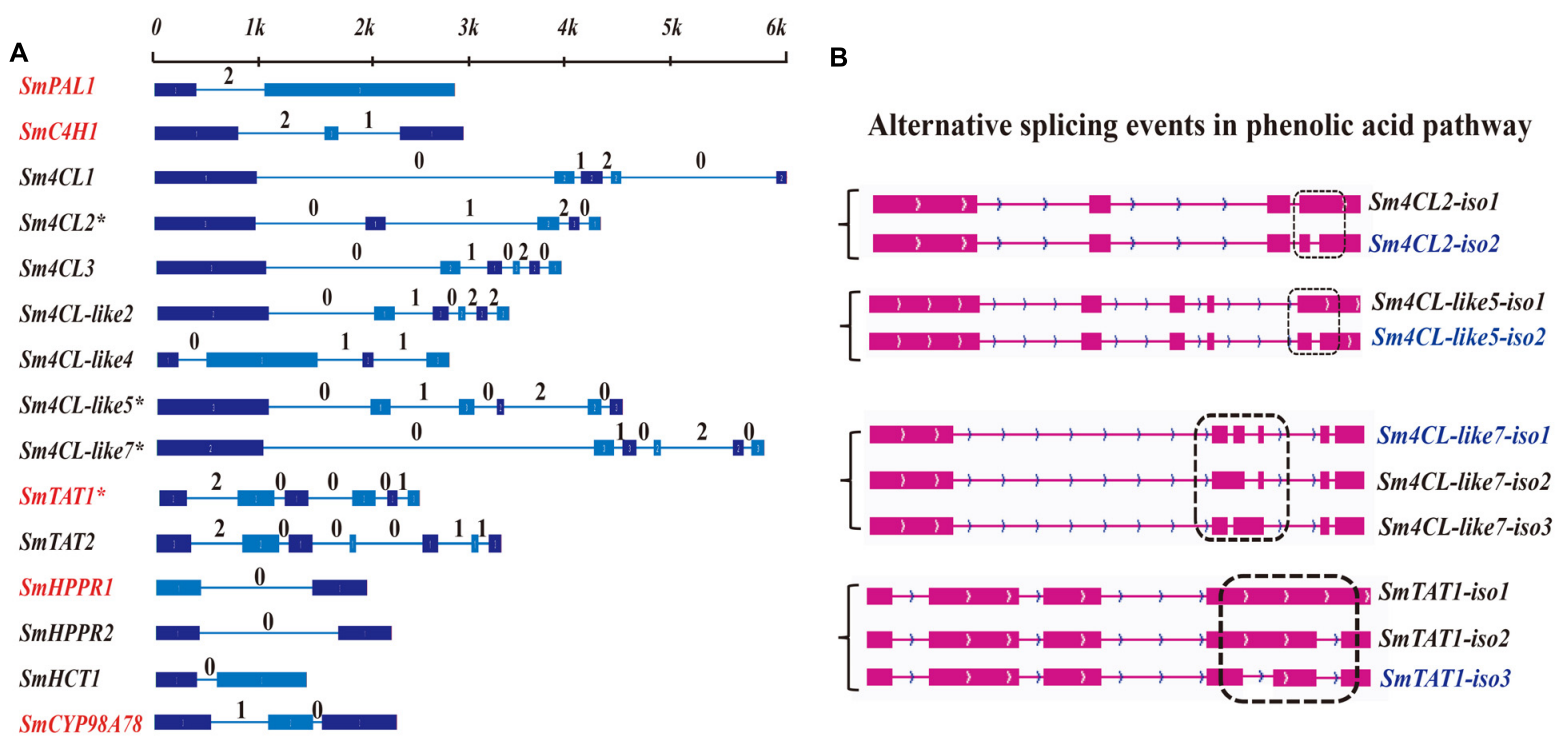
**Gene structure and alternative splicing isoforms of RA biosynthetic genes covered by PacBio long reads.** **(A)** Gene structure and intron phase of full-length transcripts covered by the PacBio survey. Red indicates that these genes have been identified in previous studies. The asterisk (^∗^) represents genes identified as alternative splicing events. **(B)** Alternative splicing isoforms related to RA.

A total of 28 candidate genes from the phenylpropanoid pathway and tyrosine-derived pathway, related to RA biosynthesis, were selected based on a genome-wide approach; these gene included *SmPALs* (3), *SmC4Hs* (2), *Sm4CLs* (10), *SmTATs* (3), *SmHPPRs* (3), *SmRASs* (6), and *SmCYP98A78* (**Supplementary Table S2** and **Figure 1C**). The same approach was previously used to detect tanshinone biosynthetic genes (Xu et al., 2015). Fifteen gene loci were detected as full-length transcripts (**Supplementary Figure S1**), and their gene structures and intron phases are described in **Figure 2A**. *SmC4H2* might be a duplicated pseudogene of *SmC4H1* with an N-terminal deletion, as *SmC4H2* exhibits 74% homology with *SmC4H1* and is located at a distance of 7.5 kb from *SmC4H1* in the genome. *Sm4CL2*, *Sm4CL-like5*, *Sm4CL-like7*, and *SmTAT1* were identified as expressing alternatively spliced isoforms (**Figure 2B**), and all of the alternatively spliced junctions were characterized as intron retention. *Sm4CL2* and *Sm4CL-like5* each expressed two isoforms, whereas *Sm4CL-like7* and *SmTAT1* each expressed three isoforms (**Supplementary Table S5**). Among their respective alternative splicing events, *Sm4CL2-iso2*, *Sm4CL-like5-iso2*, and *SmTAT1-iso3* were the dominantly expressed isoforms (**Supplementary Table S5**), though three isoforms of *Sm4CL-like7* all exhibited similar expression. We found that all of the intron retentions introduced premature termination codons (PTCs), and the PTC locations in *Sm4CL2-iso1*, *Sm4CL-like5-iso1*, *Sm4CL-like7-iso2*, *Sm4CL-like7-iso3*, *SmTAT1-iso1*, and *SmTAT1-iso2* were in intron 4, intron 5, intron 2, intron 3, intron 4, and intron 4, respectively, (**Supplementary Figure S2**).

### Expression Profiles of Candidate RA Biosynthetic Genes

In this study, analysis of differentially expressed genes in the three root tissues showed that *SmPAL1*, *SmPAL3*, *SmC4H1*, *Sm4CL3*, *Sm4CL-like1*, *Sm4CL-like4*, *SmTAT1*, *SmHPPR3*, *SmRAS*, and *SmCYP98A78* exhibited low expression in the periderm and high expression in the phloem and xylem, in accord with the distribution of LAB (**Figure 1D**). In addition, the transcript levels of *SmPAL1*, *SmC4H1*, *Sm4CL3*, *Sm4CL-like1*, *SmTAT1*, *SmHPPR3*, *SmRAS*, and *SmCYP98A78* were significantly up-regulated after 12 h of MeJA treatment (**Supplementary Table S2**); however, the expression of *Sm4CL2*, *Sm4CL-like4*, *Sm4CL-like6*, and *SmHPPR2* was down-regulated after MeJA treatment. *SmTAT3*, *SmHCT2*, *SmHCT3*, and *SmHCT4* were identified as silenced genes. *SmHCT1* exhibited remarkably specific expression in the root xylem, yet *SmHCT5* showed only slight expression in the stem. Phylogenetic trees for 18 hydroxycinnamoyltransferase (HCT family) amino acid sequences including hydroxycinnamoyl-CoA:shikimate/quinate hydroxycinnamoyltransferases (HCS/QTs), RASs, and hydroxycinnamoyl/benzoyltransferases (HCBTs), in different species revealed clustering of five unidentified HCTs from *S. miltiorrhiza* with RASs, rather than with HCS/QTs (**Supplementary Figure S3**).

### Co-expression Analysis and Isoform Identification of Candidate CYP450s

**FIGURE 3:**
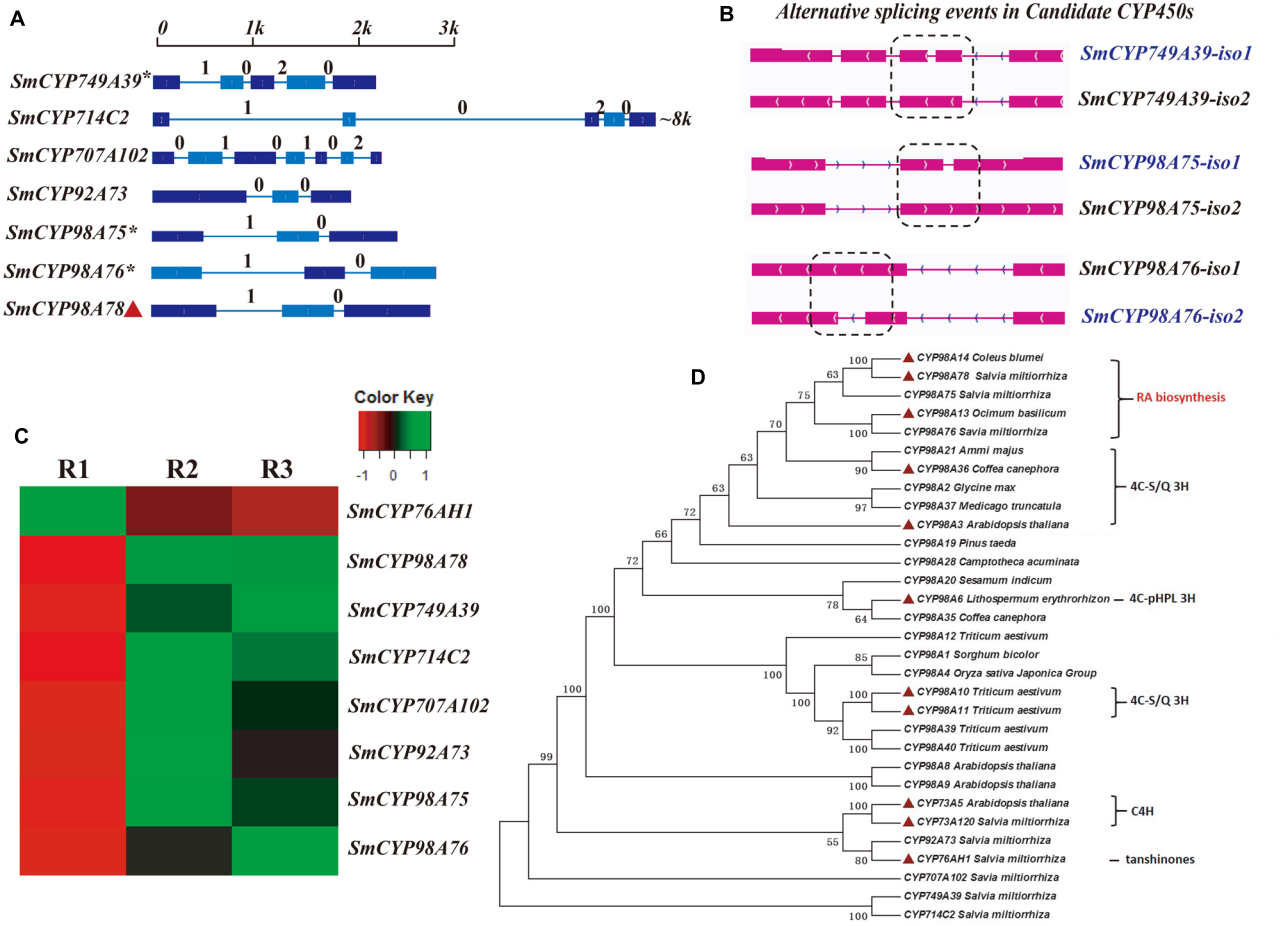
**Gene structure, alternative splicing isoforms, expression patterns, and phylogenetic tree of candidate CYP450s.** **(A)** Full-length gene structures and intron phases of candidate CYP450s related to RA. The asterisk (^∗^) indicates genes identified as alternative splicing events. **(B)** Alternative splicing isoforms of candidate CYP450s. **(C)** Differentially expressed patterns of candidate CYP450s in different root tissues. **(D)** A phylogenetic tree constructed from 31 amino acid sequences of candidate and identified CYP450s in *S. miltiorrhiza* and other species (Petersen et al., 2009). 4C-S/Q 3H, 4-coumaroylshikimate/quinate 3-hydroxylase; 4C-pHPL 3H, 4-coumaroyl-4′-hydroxyphenyllactate 3-hydroxylase. C4Hs (SmCYP73A120), CYP76AH1 from *S. miltiorrhiza* (Guo et al., 2013) and CYP73A5 from *Arabidopsis thaliana* were used as outgroups. The phylogenetic tree with respect to RA biosynthesis showed the clustering of SmCYP98A75, SmCYP98A76, and SmCYP98A78 into one branch with CYP98A14 (4C-DHPL 3H) from *C. blumei* and CYP98A13 (4C-pHPL 3H) from *Ocimum basilicum*.

**FIGURE 4:**
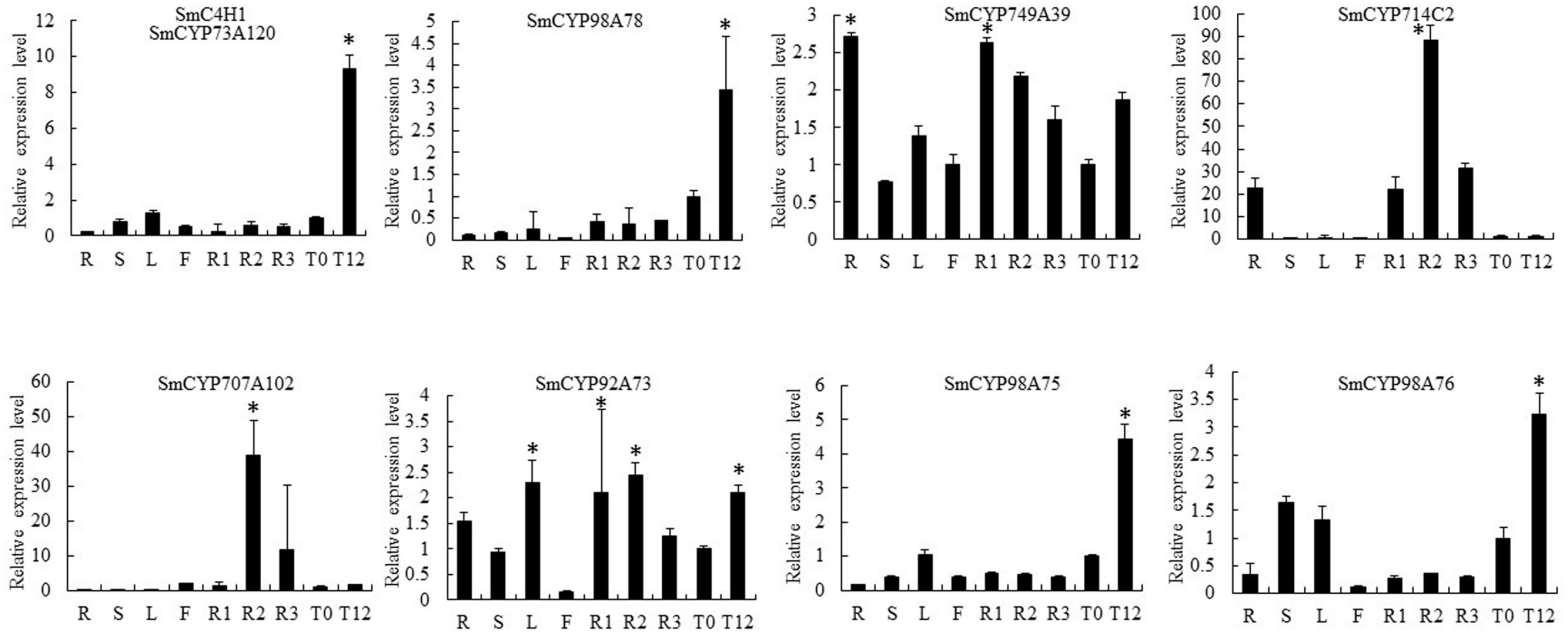
**Quantitative reverse transcription-polymerase chain reaction (qRT-PCR) analysis of candidate CYP450s with putative roles in RA biosynthesis in different tissues (periderm, phloem, xylem, root, stem, leaf, flower) and in leaves without or with MeJA treatment (MeJA-0 and MeJA-12, respectively) in *S. miltiorrhiza*.** SmC4H1 and SmCYP98A78 were used as positive controls. The asterisks (^∗^) represent significant differences in this comparison (*P* < 0.01).

Our RNA-seq results showed opposite expression patterns for *SmCYP76AH1* and *SmCYP98A78*, in accord with the different distribution of tanshinones and phenolic acids in the periderm, phloem, and xylem. Moreover, six CYP450s were selected as candidate CYP450s related to RA biosynthesis based on the criteria of a phloem/periderm FPKM greater than 1.5 and a xylem/periderm FPKM greater than 1.5. The selected CYP450s included *SmCYP749A39*, *SmCYP714C2*, *SmCYP92A73*, *SmCYP98A75*, and *SmCYP98A76* (**Supplementary Table S3**). A comprehensive evaluation of eight RNA-seq and qRT-PCR analyses of these CYP450s, including *SmCYP98A78* and *SmC4H1* (*SmCYP73A120*), indicated that expression level of the candidate CYP450s was significantly up-regulated by MeJA, with the exception of *SmCYP714C2*, which was not expressed in leaves (**Figures 3C** and **4**). Furthermore, Pearson’s correlation analysis of the qRT-PCR results showed highly significant co-expression of *SmCYP98A75*, *SmCYP98A76*, *SmCYP98A78*, and *SmC4H1* (*P* < 0.01).

All six CYP450s were identified as full-length transcripts in the PacBio transcriptome, and their gene structures and intron phases are described in **Figures 3A,B**. In addition, gene loci *SmCYP749A39*, *SmCYP98A75*, and *SmCYP98A76* were found to undergo alternative splicing events, with each expressing two gene isoforms. All alternative splicing events of these candidate CYP450s were classified as intron retention. For their respective alternative splicing events, *SmCYP749A39-iso1*, *SmCYP98A75-iso1*, and *SmCYP98A76-iso1* were found to be the dominantly expressed isoforms (**Supplementary Table S5**), and intron retention for *SmCYP749A39-iso2*, *SmCYP98A75-iso2*, and *SmCYP98A76-iso2* introduced PTCs in exon 3, intron 2, and intron 2, respectively (**Supplementary Figure S4**).

To better understand the putative functions of these candidate CYP450s, we constructed a phylogenetic tree with 31 full-length amino acid sequences from various species, including some functionally identified CYP98As. CYP92A73 clustered into one branch with CYP76AH1, which was identified as catalyzing the miltiradiene to ferruginol step in tanshinone biosynthesis (**Figure 3D**). These two CYP450s were found to be neighbors of C4H from *S. miltiorrhiza* and *A. thaliana* (**Figure 3D**). The other predicted CYP450s, SmCYP707A102, SmCYP749A39, and SmCYP714C2, were distant from the CYP98A subfamily (**Figure 3D**).

### Co-expression Analysis and Isoform Identification of Candidate Laccases

**FIGURE 5:**
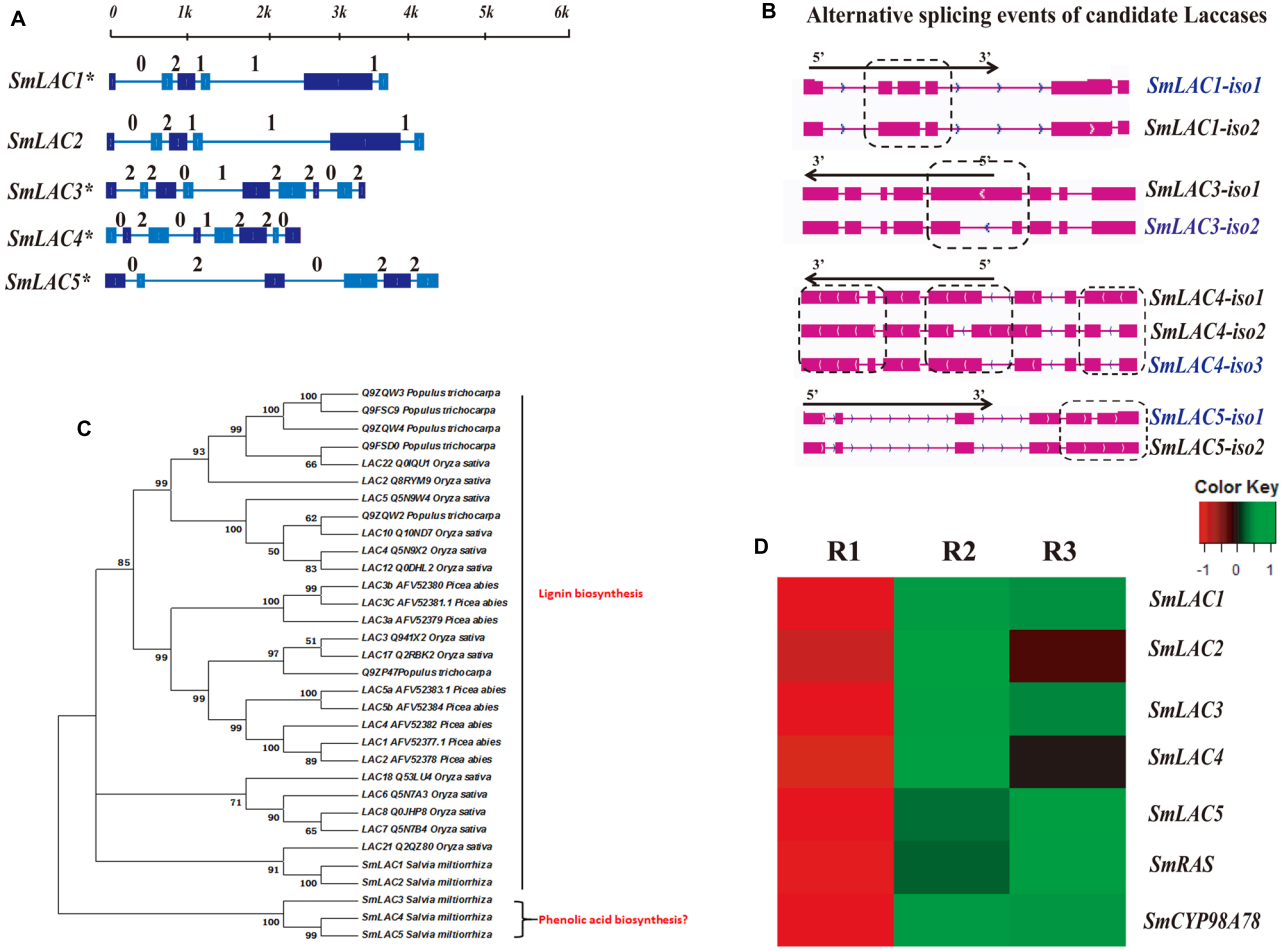
**Gene structure, alternative splicing isoforms, expression patterns, and phylogenetic tree of candidate laccases.** **(A)** Full-length gene structures and intron phases of candidate laccases related to SA. The asterisk (^∗^) indicates genes identified as alternative splicing events. **(B)** Alternative splicing isoforms of candidate laccases. **(C)** Differentially expressed patterns of candidate laccases in different root tissues. SmCYP98A78 was used as a positive control. **(D)** A phylogenetic tree constructed using 32 amino acid sequences of candidate laccases in *S. miltiorrhiza* and lignin biosynthetic laccases in other species.

**FIGURE 6:**
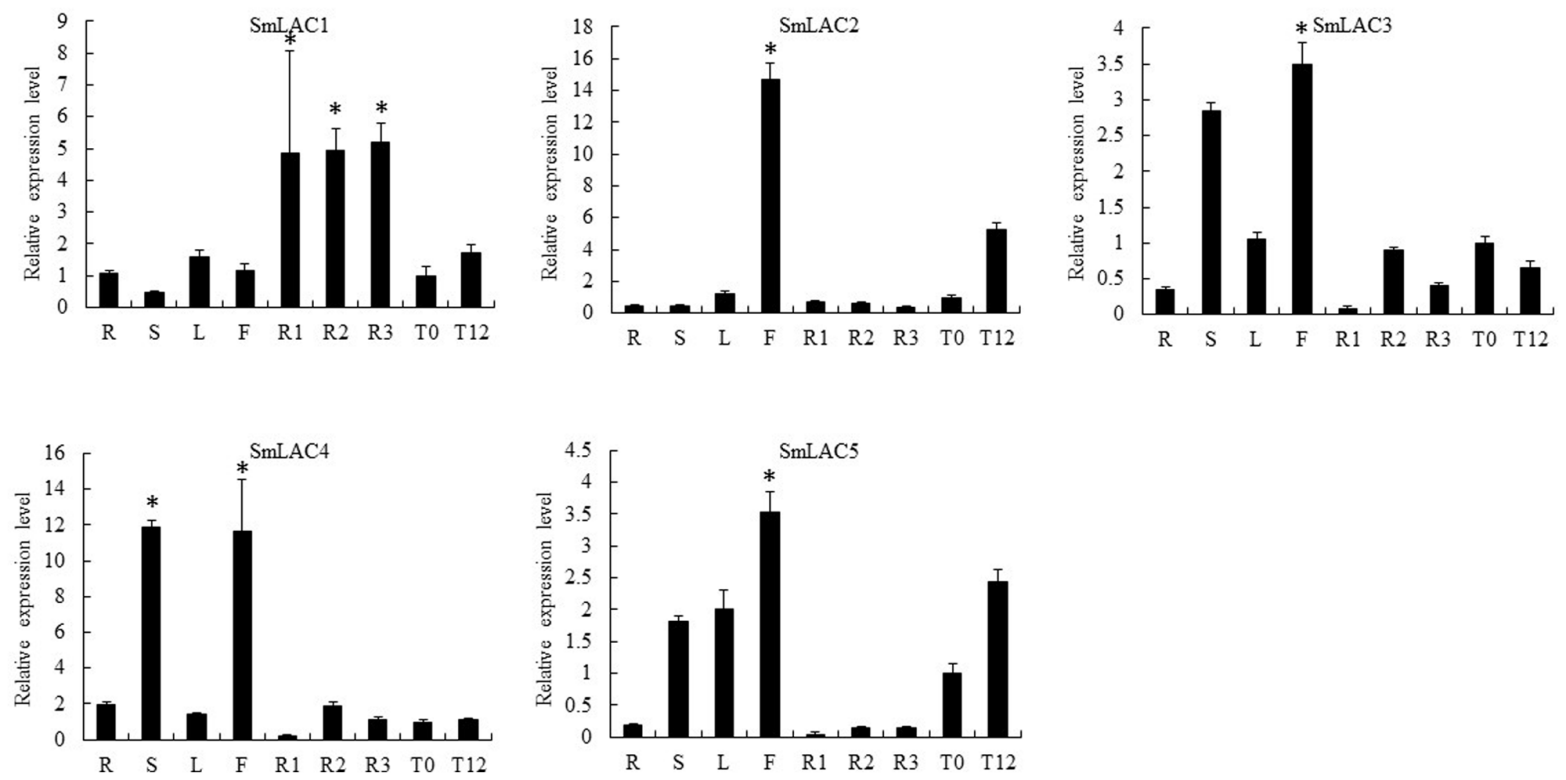
**Quantitative reverse transcription-polymerase chain reaction analysis of the candidate laccases with putative roles in SA biosynthesis in different tissues (periderm, phloem, xylem, root, stem, leaf, flower) and in leaves without or with MeJA treatment (MeJA-0 and MeJA-12, respectively) in *S. miltiorrhiza*.** The asterisks (^∗^) represent significant differences in this comparison (*P* < 0.01).

Eighty laccases were identified through genome-wide analysis in *S. miltiorrhiza*, with five identified based on the criterion of higher expression in the phloem and xylem than in the periderm: *SmLAC1*, *SmLAC2*, *SmLAC3*, *SmLAC4*, and *SmLAC5* (**Figure 5C**, **Supplementary Table S4**). Furthermore, RNA-seq and qRT-PCR analyses indicated that MeJA up-regulated the expression of *SmLAC1*, *SmLAC2* and *SmLAC5* (**Figures 5C** and **6**). According to the qRT-PCR data, *SmLAC5* was significantly co-expressed (*P* < 0.05) with *SmCYP98A78* and *SmC4H1*.

All five laccases were identified as full-length transcripts in the PacBio transcriptome, and their gene structures and intron phases are described in **Figures 5A,B**. In addition, the *SmLAC1*, *SmLAC3*, *SmLAC4*, and *SmLAC5* gene loci were found to undergo alternative splicing events, with *SmLAC1*, *SmLAC3*, and *SmLAC5* each expressing two isoforms and *SmLAC4* three isoforms. All of the alternative splicing events in these selected laccases were classified as intron retention. *SmLAC1-iso1*, *SmLAC3-iso2*, *SmLAC4-iso3*, and *SmLAC5-iso1* were the dominantly expressed isoforms among their respective alternative splicing events (**Supplementary Table S5**), and the intron retention in *SmLAC1-iso2*, *SmLAC3-iso1*, *SmLAC4-iso1*, *SmLAC4-iso2*, and *SmLAC5-iso2* introduced PTCs in intron 2, intron 4, intron 1, intron 4, and intron 5, respectively (**Supplementary Figure S5**).

To predict the functions of the candidate laccases, a phylogenetic tree was constructed using 32 amino acid sequences from *Populus trichocarpa*, *Picea abies*, *Oryza sativa*, and *S. miltiorrhiza*. SmLAC3, SmLAC4, and SmLAC5 were classified into different branches with other laccases that have been described as closely correlated with lignin biosynthesis in other species (**Figure 5D**).

## Discussion

In this study, we analyzed full-length transcripts and alternative splicing events related to phenolic acid biosynthesis in different root tissue of *S. miltiorrhiza* by combining NGS and TGS technologies. Previous studies have only cloned a small number of full-length genes, such as *SmPAL1*, *SmC4H1*, *SmTAT1*, *SmHPPR1*, *SmRAS*, and *SmCYP98A78*, and identified their functions (Huang et al., 2008; Song and Wang, 2009, 2011; Xiao et al., 2009a, 2011; Di et al., 2013). Despite predicted locations and functions based on genome annotation, other full-length homologous genes and their functions have not yet been identified (Wang et al., 2015). Among the 28 homologous genes identified as being involved in RA biosynthesis, the ability to detect 68% of full-length transcripts (15 full-length transcripts/22 expressed genes) and 27% of alternative splicing events at gene loci (4/15) indicates a significant advantage of hybrid sequencing in such discovery (**Supplementary Table S2**). Indeed, the availability of full-length transcripts will allow for establishing a metabolic engineering strategy with the aim of modulating the phenolic acid content, and the identification of alternative splicing events is beneficial for understanding the molecular mechanisms of phenolic acid biosynthesis in *S. miltiorrhiza*.

In line with our interest in phenolic acid biosynthesis, we found that not only the distribution of LAB but also the major expression of phenolic acid biosynthetic genes in the root occurred in the phloem and xylem (**Figure 1**). This agreement between phytochemical assay and gene expression in the root provided a basis for co-expression analysis. In addition, MeJA was found to dramatically promote the accumulation of phenolic acids and the expression of key genes (Xiao et al., 2009b). Although many genes related to RA biosynthesis have been cloned and identified in other species, 28 homologous genes based on genome-wide identification generated more candidates to assist in fully elucidating the RA biosynthetic pathway in *S. miltiorrhiza*. *4CL1* and *4CL2* of phenylpropanoid pathway have been cloned and their functions identified *in vitro* (Zhao et al., 2006); however, the 4CL catalyzing 4-cinnamic acid to 4-coumaroyl-CoA *in vivo* remains unknown. *Sm4CL3*, *Sm4CL-like1*, and *Sm4CL-like4* are most likely to be involved in the synthesis of RA. The overexpression of *SmHPPR1* in tyrosine-derived pathway resulted in the accumulation of 4-hydroxyphenylpyruvic acid, the substrate of SmHPPR (Xiao et al., 2011). However, *SmHPPR3*, rather than *SmHPPR1*, might participate in RA biosynthesis. An additional step from 4-hydroxyohenyllactic acid to 3,4-dihydroxyphenyllactic acid was found in *S. miltiorrhiza* using a C^13^ tracer. As this step is likely to be catalyzed by an unknown CYP450 (Di et al., 2013), we then selected six CYP450s that were more significantly expressed in the phloem and xylem than in the periderm (**Supplementary Table S3**). According to phylogenetic tree and qRT-PCR analyses, CYP98A75 and CYP98A76 likely participate in RA biosynthesis rather than as 4-coumaroylshikimate/quinate 3-hydroxylases in quinic acid and shikimic acid biosynthesis (Chen et al., 2014). A previous study proposed that a laccase was potentially involved in the oxidative dimerization of RA to synthesize LAB (Di et al., 2013). To explore the reactions that convert RA to LAB, five laccases were identified as exhibiting higher expression in the phloem and xylem than in the periderm (**Supplementary Table S4**). Furthermore, *SmLAC5* was considered to be the best candidate for LAB synthesis. Further studies of these candidate CYPs and laccases using transgenic methods and biochemical reactions may accurately elucidate the mechanism of phenolic acid biosynthesis.

The complexity of alternative splicing events plays a potentially important regulatory role in SA biosynthesis, and many studies focusing on alternative splicing events in *Arabidopsis* have been reported (Filichkin et al., 2010; Marquez et al., 2012). In contrast to humans, the most common type of alternative splicing event in plants appears to be intron retention (Au et al., 2013; Reddy et al., 2013). In fact, all of the identified alternative splicing events in *S. miltiorrhiza* SA biosynthesis showed intron retention. A recent study reported that most of the intron retention isoforms in *Arabidopsis* are predicted to be targets of nonsense-mediated decay (NMD) to regulate mRNA stability. Expect for *Sm4CL-like7*, the alternative splicing events related to SA biosynthesis in *S. miltiorrhiza* expressed one predominant isoform (**Supplementary Table S5**). Low-expression isoforms have been described as splicing errors, which commonly trigger NMD to maintain mRNA stability (Filichkin et al., 2010; Zhang et al., 2010; Marquez et al., 2012; Reddy et al., 2013; Walters et al., 2013; Shen et al., 2014). In addition, the highly expressed genes *Sm4CL-like7-iso2* and *Sm4CL-like7-iso3*, which contain PTCs downstream of the splice junctions, might be subjected to NMD to eliminate incomplete transcripts (**Supplementary Figure S2**). Another prediction about these two PTC isoforms of *Sm4CL-like7* is that small interfering peptides with absent functional domains could form non-functional dimers that compete with and negatively regulate functional proteins. Our results clearly detected and predicted alternative splicing events related to SA biosynthesis, though the actual functions of the alternative splicing isoforms remain unknown. Thus, the systematic identification of co-expression, full-length transcripts and alternative splicing events related to the biosynthesis of lipophilic diterpenoid pigments (Xu et al., 2015) and hydrophilic phenolic acids in various root tissues of *S. miltiorrhiza* could better resolve the biology of the synthesis of such natural products.

In summary, we localized SA metabolism in the medicinal plant *S. miltiorrhiza* to the root phloem and xylem. We then identified full-length transcripts, encoding isoforms as well as alternative splicing events in SA biosynthesis and systematically analyzed six candidate CYP450s and five candidate laccases related to SA biosynthesis in *S. miltiorrhiza* using hybrid sequencing. Furthermore, our study provides a model for analyzing the full-length transcriptome and the biosynthesis of active constituents in other medicinal plants.

## Author Contributions

ZX, JS, and SC designed and coordinated the study. ZX, HL, XZ, and AJ performed experiments and analyzed the data. ZX, JS, HL, and SC wrote the manuscript.

## Conflict of Interest Statement

The authors declare that the research was conducted in the absence of any commercial or financial relationships that could be construed as a potential conflict of interest.
