# Supplementary material for: Global Identification of the Full-Length Transcripts and Alternative Splicing Related to Phenolic Acid Biosynthetic Genes in Salvia miltiorrhiza
Source: Front Plant Sci. 2016 Feb 5;7:100. doi: 10.3389/fpls.2016.00100 (PMC4742575; doi:10.3389/fpls.2016.00100)
Supplement: Supplementary file 1 [file Data_Sheet_1.PDF]

SUPPORTING INFORMATION

Global identification of the full-length transcripts and alternative splicing related to salvianolic acid biosynthetic genes in *Salvia miltiorrhiza*

Zhichao Xu[1], Hongmei Luo[1], Aijia Ji[1], Xin Zhang[1], Jingyuan Song[1*] and Shilin Chen[1,2*]

[1]Institute of Medicinal Plant Development, Chinese Academy of Medical Sciences & Peking Union Medical College, Beijing, China
[2]Institute of Chinese Materia Medica, Chinese Academy of Chinese Medical Science, Beijing, 100700, China

[*]Authors to whom corresponding should be addressed: No. 151, Malianwa North Rd., HaiDian District, Beijing 100193, China. E-mail: jysong@implad.ac.cn; and No. 16, Dongzhimenneinanxiaojie, Dongcheng District, Beijing, 100700, China. E-mail: chenshilin@icmm.ac.cn.

#SmPAL1

```
SmPAL1-hybrid-seq         TAAACCAACGCAAACGTGTGTGTTCGACGGCGATCAATCCATGGCGGCGGAGAACGGTCATCACGAGGAGTCCAACGG
SmPAL1-EF462460.1         ----------------------------------------ATGGCGGCGGAGAACGGTCATCACGAGGAGTCCAACGG
SmPAL1-genome_annotation  ----------------------------------------ATGGCGGCGGAGAACGGTCATCACGAGGAGTCCAACGG

SmPAL1-hybrid-seq         CTTCTGCGTCAAGCAGAACGATCCCTTGAACTGGGTGGCGGCGGCGGAGTCGCTCAAGGGGAGCCACCTCGACGAGGT
SmPAL1-EF462460.1         CTTCTGCGTCAAGCAGAACGATCCCTTGAACTGGGTGGCGGCGGCGGAGTCGCTCAAGGGGAGCCACCTCGACGAGGT
SmPAL1-genome_annotation  CTTCTGCGTCAAGCAGAACGATCCCTTGAACTGGGTGGCGGCGGCGGAGTCGCTCAAGGGGAGCCACCTCGACGAGGT

SmPAL1-hybrid-seq         TAAGCGGATGGTGGAGGAGTTCAGGAAGCCGGTGGTCAAGCTCGGCGGGGAGACGCTCACCATATCTCAGGTGGCGGC
SmPAL1-EF462460.1         TAAGCGGATGGTGGAGGAGTTCAGGAAGCCGGTGGTCAAGCTCGGCGGGGAGACGCTCACCATATCTCAGGTGGCGGC
SmPAL1-genome_annotation  TAAGCGGATGGTGGAGGAGTTCAGGAAGCCGGTGGTCAAGCTCGGCGGGGAGACGCTCACCATATCTCAGGTGGCGGC

SmPAL1-hybrid-seq         GATCGCGGCCAAGGATAATGCGGTGGCGGTGGAGCTGGCCGAGTCGTCCAGGGCCGGCGTCAAGGCCAGCAGCGATTG
SmPAL1-EF462460.1         GATTGCGGCCAAGGATAATGCGGTGGCGGTGGAGCTGGTCGAGTCGTCCAGGGCCGGCGTCAAGGCCAGCAGTGATTG
SmPAL1-genome_annotation  GATCGCGGCCAAGGATAATGCGGTGGCGGTGGAGCTGGCCGAGTCGTCCAGGGCCGGCGTCAAGGCCAGCAGCGATTG

SmPAL1-hybrid-seq         GGTTATGGAGAGCATGAGTAAAGGCACCGACAGCTACGGCGTCACCACCGGTTTCGGTGCCACCTCTCACCGGAGGAC
SmPAL1-EF462460.1         GGTTATGGAGAGCATGAGTAAGGGCACCGACAGCTACGGCGTCACCACCGGTTTCGGTGCCACCTCTCACCGGAGGAC
SmPAL1-genome_annotation  GGTTATGGAGAGCATGAGTAAAGGCACCGACAGCTACGGCGTCACCACCGGTTTCGGTGCCACCTCTCACCGGAGGAC

SmPAL1-hybrid-seq         CAAGCAGGGCGGCGCTCTTCAGAAGGAGCTCATTAGGTTCTTGAACGCCGGAATATTCGGAAACGGGACAGAATCCAA
SmPAL1-EF462460.1         CAAGCAGGGCGGCGCTCTTCAGAAGGAGCTCATTAGGTTCTTGAACGCCGGAATATTCGGAAACGGGACAGAATCCAA
SmPAL1-genome_annotation  CAAGCAGGGCGGCGCTCTTCAGAAGGAGCTCATTAGGTTCTTGAACGCCGGAATATTCGGAAACGGGACAGAATCCAA

SmPAL1-hybrid-seq         CCACACGCTGCCGCACACGGCGACGAGAGCGGCGATGCTGGTTCGGATCAACACCCTCCTCCAAGGCTACTCCGGCAT
SmPAL1-EF462460.1         CCACACGCTGCCGCACACGGCGACGAGAGCGGCGATGCTGGTTCGGATCAACACCCTCCTCCAAGGCTACTCCGGCAT
SmPAL1-genome_annotation  CCACACGCTGCCGCACACGGCGACGAGAGCGGCGATGCTGGTTCGGATCAACACCCTCCTCCAAGGCTACTCCGGCAT

SmPAL1-hybrid-seq         CATATTCTAAATCCTGGAATCCATCACTAAATTCCGTAACTAGTAACATTCACCCCATTGCCTCCCCCTCCTCTGCAC
SmPAL1-EF462460.1         CAGATTCGAAATCCTGGAAGCCATCACTAAATTCCTGAACGAG-AACA-TCACCCCA-TGCCTCCCCCTCCGCGGCAC
SmPAL1-genome_annotation  CAGATTCGAAATCCTGGAAGCCATCACTAAATTCCTGAACGAG-AACA-TCACCCCA-TGCCTCCCCCTCCGCGGCAC

SmPAL1-hybrid-seq         CATCACGGCCTCCTCTATCTTTTTCCTGCTATCCTACATCGC--CCTTCCTTAC-GTCCGCCCAAACTCTAAGTCTGT
SmPAL1-EF462460.1         CATCACGGCCTCCGGCGATCTGGTGCCGCTATCCTACATCGCGGGCCTCCTGACGGGGCGGCCCAACTCGAAGGCTGT
SmPAL1-genome_annotation  CATCACGGCCTCCGGCGATCTGGTGCCGCTATCCTACATCGCGGGCCTCCTGACGGGGCGGCCCAACTCGAAGGCTGT

SmPAL1-hybrid-seq         -GTCCCCAACGTTCGAGCCCCTTAACTGCGAGTAATCCTTCATCTTTTGCCGTTCG--TTGAAATTCTTCTTCTTCTA
SmPAL1-EF462460.1         GGGCCCCAACG-GCGAGCCCCTGAACGCGGAGGAAGCCTTCA-----AGCTGGCCGGCGTGAAAGGCGGCTTCTTCGA
SmPAL1-genome_annotation  GGGCCCCAACG-GCGAGCCCCTGAACGCGGAGGAAGCCTTCA-----AGCTGGCCGGCGTGAAAGGCGGCTTCTTCGA

SmPAL1-hybrid-seq         TCTTGCATCCCAATGAATTTCTTTCCCTGTTAACT--CACCTCCGTTGTTCCGTTACTAGCCTCCATCGTCCCTCTTC
SmPAL1-EF462460.1         GC-TGCAGCCCAAGGAAGGGCTGGCCCTGGTGAACGGCACCGCCGTGGGGTCCGGACTGGCCTCCATCG-CCCTCTTC
SmPAL1-genome_annotation  GC-TGCAGCCCAAGGAAGGGCTGGCCCTGGTGAACGGCACCGCCGTGGGGTCCGGACTGGCCTCCATCG-CCCTCTTC
```

```
SmPAL1-hybrid-seq          TACTCCAACATCCTGGCCGTCCTATCGGAGGTGATGTCCGCCGTGTTCGCGGAGGTGATGAACGGGAAGCCGGAGTTC
SmPAL1-EF462460.1          GACGCCAACATCCTGGCCGTCCTATCGGAGGTGATGTCCGCCGTGTTCGCGGAGGTGATGAACGGGAAGCCGGAGTTC
SmPAL1-genome_annotation GACGCCAACATCCTGGCCGTCCTATCGGAGGTGATGTCCGCCGTGTTCGCGGAGGTGATGAACGGGAAGCCGGAGTTC

SmPAL1-hybrid-seq          ACGGATCACCTGACGCACAAGCTGAAGCACCACCCGGGGCAGATCGAGGCGGCGGCGATCATGGAGCACATCCTGGAC
SmPAL1-EF462460.1          ACGGATCACCTGACGCACAAGCTGAAGCACCACCCGGGGCAGATCGAGGCGGCGGCGATCATGGAGCACATCCTGGAC
SmPAL1-genome_annotation ACGGATCACCTGACGCACAAGCTGAAGCACCACCCGGGGCAGATCGAGGCGGCGGCGATCATGGAGCACATCCTGGAC

SmPAL1-hybrid-seq          GGCAGCGGGTACGTGAAGGCGGCGCAGAAGCTGCACGAGCAGGATCCCCTGCAGAAGCCCAAGCAGGACAGGTACGCC
SmPAL1-EF462460.1          GGCAGCGGGTACGTGAAGGCGGCGCAGAAGCTGCACGAGCAGGATCCCCTGCAGAAGCCCAAGCAGGACAGGTACGCC
SmPAL1-genome_annotation GGCAGCGGGTACGTGAAGGCGGCGCAGAAGCTGCACGAGCAGGATCCCCTGCAGAAGCCCAAGCAGGACAGGTACGCC

SmPAL1-hybrid-seq          CTCCGCACCTCGCCCCAGTGGCTGGGCCCGCAGATTGAGGTCATCCGCACCGCCACCAAGATGATCGAGAGGGAGATC
SmPAL1-EF462460.1          CTCCGCACCTCGCCCCAGTGGCTGGGCCCGCAGATTGAGGTCATCCGCACCGCCACCAAGATGATCGAGAGGGAGATC
SmPAL1-genome_annotation CTCCGCACCTCGCCCCAGTGGCTGGGCCCGCAGATTGAGGTCATCCGCACCGCCACCAAGATGATCGAGAGGGAGATC

SmPAL1-hybrid-seq          AACTCCGTCAACGACAACCCCTTGATCGATGTTTCTAGAAACAAGGCCTTGCACGGTGGAAACTTCCAAGGCACCCCC
SmPAL1-EF462460.1          AACTCCGTCAACGACAACCCCTTGATCGATGTTTCTAGAAACAAGGCCTTGCACGGTGGAAACTTCCAAGGCACCCCC
SmPAL1-genome_annotation AACTCCGTCAACGACAACCCCTTGATCGATGTTTCTAGAAACAAGGCCTTGCACGGTGGAAACTTCCAAGGCACCCCC

SmPAL1-hybrid-seq          ATCGGAG--TCCATGGACAATGCCCGCCTAGCCATCGCCTCCATCGGGAAGCTTCTCTTCGCTCAGTTCTCCGAGCTC
SmPAL1-EF462460.1          ATCGGAGTTTCCATGGACAATGCCCGCCTAGCCATCGCCTCCATCGGGAAGCTTCTCTTCGCTCAGTTCTCCGAGCTC
SmPAL1-genome_annotation ATCGGAGTTTCCATGGACAATGCCCGCCTAGCCATCGCCTCCATCGGGAAGCTTCTCTTCGCTCAGTTCTCCGAGCTC

SmPAL1-hybrid-seq          GTCAACGATTTCTACAACAACGGACTGCCGTCCAACCTCTCCGGCGGCCGCAACCCCAGCCTCGACTACGGCTTCAAG
SmPAL1-EF462460.1          GTCAACGATCTCTACAACAACGGATTGCCGTCCAACCTCTCCGGCGGCCGCAACCCCAGCCTCGACTACGGCTTCAAG
SmPAL1-genome_annotation GTCAACGATTTCTACAACAACGGACTGCCGTCCAACCTCTCCGGCGGCCGCAACCCCAGCCTCGACTACGGCTTCAAG

SmPAL1-hybrid-seq          GGCTCCGAGATCGCCATGGCCTCCTACTGCTCCGAGCTCCAGTTCCTGGCCAACCCCGTCACCAACCACGTGCAGCGC
SmPAL1-EF462460.1          GGCTCCGAGATCGCCATGGCCTCCTACTGCTCCGAGCTCCAGTTCCTGGCCAACCCCGTCACCAACCACGTGCAGAGC
SmPAL1-genome_annotation GGCTCCGAGATCGCCATGGCCTCCTACTGCTCCGAGCTCCAGTTCCTGGCCAACCCCGTCACCAACCACGTGCAGAGC

SmPAL1-hybrid-seq          TCCGAGCAGCACAACCAGGATGTCAACTCATTGGGCTTGATCTCTTCACGGAAGACCGTTGAAGCTCTCGACATTCTT
SmPAL1-EF462460.1          GCCGAGCAGCACAACCAGGATGTCAACTCATTGGGCTTGATCTCTTCACGGAAGACCGTTGAAGCTCTCGACATTCTT
SmPAL1-genome_annotation GCCGAGCAGCACAACCAGGATGTCAACTCATTGGGCTTGATCTCTTCACGGAAGACCGTTGAAGCTCTCGACATTCTT

SmPAL1-hybrid-seq          AAACTCATGTCGTCCACCTACCTCGTCGCCCTATGCCAAGCCGTGGATTTGAGGCATTTGGAGGAGAATCTCAAGCAC
SmPAL1-EF462460.1          AAACTCATGTCGTCCACCTACCTCGTCGCCCTATGCCAAGCCGTGGATTTGAGGCATTTGGAGGAGAATCTCAAGCAC
SmPAL1-genome_annotation AAACTCATGTCGTCCACCTACCTCGTCGCCCTATGCCAAGCCGTGGATTTGAGGCATTTGGAGGAGAATCTCAAGCAC

SmPAL1-hybrid-seq          GCGGTGAAGAACACCGTGAGCCAGGTTGCTAAACGAACTCTCACAATGGGCGTCAATGGCGAGCTCCATCCTTCCAGA
SmPAL1-EF462460.1          GCGGTGAAGAACACCGTGAGCCAGGTTGCTAAACGAACTCTCACAATGGGCGTCAATGGCGAGCTCCATCCTTCCAGA
SmPAL1-genome_annotation GCGGTGAAGAACACCGTGAGCCAGGTTGCTAAACGAACTCTCACAATGGGCGTCAATGGCGAGCTCCATCCTTCCAGA
```

```
SmPAL1-hybrid-seq         TTCTGCGAGAAGGACTTGATCCGCGTGGTCGACCGCGAGTACGTTTTCGCCTACATCGACGACCCCTGCAGCGCCACC
SmPAL1-EF462460.1         TTCTGCGAGAAGGACTTGATCCGCGTGGTCGACCGCGAGTACGTTTTCGCCTACATCGACGACCCCAGCAGCGCCACC
SmPAL1-genome_annotation  TTCTGCGAGAAGGACTTGATCCGCGTGGTCGACCGCGAGTACGTTTTCGCCTACATCGACGACCCCTGCAGCGCCACC

SmPAL1-hybrid-seq         TACCCCTTGATGCAGAAGCTGAGGCAGGTGCTCGTCGATCACGCCCTCAAGAACGGGGACTTGGAGAAGAACGCGAGC
SmPAL1-EF462460.1         TACCCCTTGATGCAGAAGCTGAGGCAGGTGCTCGTCGATCACGCCCTCAAGAACGGGGACTTGGAGAAGAACGCGAGC
SmPAL1-genome_annotation  TACCCCTTGATGCAGAAGCTGAGGCAGGTGCTCGTCGATCACGCCCTCAAGAACGGGGACTTGGAGAAGAACGCGAGC

SmPAL1-hybrid-seq         ACCTCGATCTTCCAGAAGATCGAGGCGTTCGAGGAGGAGCTCAAGGCCTTATTGCCCAAGGAGGTGGAGAGCGCGAGG
SmPAL1-EF462460.1         ACCTCGATCTTCCAGAAGATCGAGGCGTTCGAGGAGGAGCTCAAGGCCTTATTGCCCAAGGAGGTGGGGAGCGCGAGG
SmPAL1-genome_annotation  ACCTCGATCTTCCAGAAGATCGAGGCGTTCGAGGAGGAGCTCAAGGCCTTATTGCCCAAGGAGGTGGAGAGCGCGAGG

SmPAL1-hybrid-seq         ATGGCCCTCGAGAGCGGGAGCCCCACCGTGGCCAACCGGATAGCGGAGTGCAGGTCGTACCCGTTGTATAAGTTCATA
SmPAL1-EF462460.1         ATGGCCCTCGAGAGCGGGAGCCCCACCGTGGCCAACCGGATAGCGGAGTGCAGGTCGTACCCGTTGTATAAGTTCATA
SmPAL1-genome_annotation  ATGGCCCTCGAGAGCGGGAGCCCCACCGTGGCCAACCGGATAGCGGAGTGCAGGTCGTACCCGTTGTATAAGTTCATA

SmPAL1-hybrid-seq         AGGGAGCAGCTCGGCGCGGGGTTCTTGACGGGGGAGAAGGCGGTGTCGCCGGGGGAGGAGTGCGAGAAGGTGTTCACG
SmPAL1-EF462460.1         AGGGAGCAGCTCGGCGCGGGGTTCTTGACGGGGGAGAAGGCGGTGTCGCCGGGGGAGGAGTGCGAGAAGGTGTTCACG
SmPAL1-genome_annotation  AGGGAGCAGCTCGGCGCGGGGTTCTTGACGGGGGAGAAGGCGGTGTCGCCGGGGGAGGAGTGCGAGAAGGTGTTCACG

SmPAL1-hybrid-seq         GCGTTGAGCAATGGCCTCATTATTGACCCCTTGTTGGAATGCCTTCAAGGATGGAATGGTCAACCTCTGCCAATCTGC
SmPAL1-EF462460.1         GCGTTGAGCAATGGCCTCATTATTGACCCCTTGTTGGAATGCCTTCAAGGATGGAATGGTCAACCTCTGCCAATCTGC
SmPAL1-genome_annotation  GCGTTGAGCAATGGCCTCATTATTGACCCCTTGTTGGAATGCCTTCAAGGATGGAATGGTCAACCTCTGCCAATCTGC

SmPAL1-hybrid-seq         TAGTTCGTTTTCTATATGTTTCGCCCAAATAATTTTTTTCAATTTGTTTTCTGTGCTTAAATTTGCTCTTTTTTTTCA
SmPAL1-EF462460.1         TAG---------------------------------------------------------------------------
SmPAL1-genome_annotation  TAG---------------------------------------------------------------------------

SmPAL1-hybrid-seq         TTTGTGTTCTTCTTCGACGGAATCTCCTGTGTTTTGTGAAATAATGTGTAAAATTCAATGGGATTCTCGCGTTTGCAA
SmPAL1-EF462460.1         ------------------------------------------------------------------------------
SmPAL1-genome_annotation  ------------------------------------------------------------------------------
#SmC4H1
SmC4H1-hybrid-seq         -------------AAAAACCACCAACTCCCGCCATCACCACCGCCACTGCAGACCGCAAACCATTTTAAAATGGATCT
SmC4H1-DQ355979.1         GCGGGGCAGTTCCAAAAACCACCAACTCCCGCCATCACCACCGCCACTGCAGACCGCAACCCATTTTAAAATGGATCT
SmC4H1-genome_annotation  ----------------------------------------------------------------------ATGGATCT

SmC4H1-hybrid-seq         CCTCCTCCTCGAGAAGGCGCTAATAGGCCTCTTCTCCGCCATCGTCGTCGCCGCGGTGGTGTCGAAGCTCCGCGGCAA
SmC4H1-DQ355979.1         CCTCCTCCTCGAGAAGGCGCTAATAGGCCTCTTCTCCGCCATCGTCGTCGCCGCGGTGGTGTCGAAGCTCCGCGGCAA
SmC4H1-genome_annotation  CCTCCTCCTCGAGAAGGCGCTAATAGGCCTCTTCTCCGCCATCGTCGTCGCCGCGGTGGTGTCGAAGCTCCGCGGCAA

SmC4H1-hybrid-seq         GAAATTCAAGTTGCCGCCGGGACCGATTCCGGTCCCAATCTTCGGCAATTGGCTCCAGGTCGGGGATGATCTGAACCA
SmC4H1-DQ355979.1         GAAATTCAAGTTGCCGCCGGGACCGATTCCGGTCCCAATCTTCGGCAATTGGCTCCAGGTCGGGGATGATCTGAACCA
SmC4H1-genome_annotation  GAAATTCAAGTTGCCGCCGGGACCGATTCCGGTCCCAATCTTCGGCAATTGGCTCCAGGTCGGGGATGATCTGAACCA
```

SmC4H1-hybrid-seq CCGCAACCTCACTGACTACGCGAAGCGTTTCGGCGACATCTTCCTCCTCCGCATGGGGCAGCGCAACCTCGCCGTCGT

SmC4H1-DQ355979.1 CCGCAACCTCACTGACTACGCGAAGCGTTTCGGCGACATCTTCCTCCTCCGCATGGGGCAGCGCAACCTCGCCGTCGT

SmC4H1-genome_annotation CCGCAACCTCACTGACTACGCGAAGCGTTTCGGCGACATCTTCCTCCTCCGCATGGGGCAGCGCAACCTCGCCGTCGT

SmC4H1-hybrid-seq GTCGTCGCCGGAGTTGGCGAAGGAGGTCCTCCACACGCAGGGCGTGGAGTTCGGATCGCGCACGCGCAACGTCGTGTT

SmC4H1-DQ355979.1 GTCGTCGCCGGAGTTGGCGAAGGAGGTCCTCCACACGCAGGGCGTGGAGTTCGGGTCGCGCACGCGCAACGTCGTGTT

SmC4H1-genome_annotation GTCGTCGCCGGAGTTGGCGAAGGAGGTCCTCCACACGCAGGGCGTGGAGTTCGGATCGCGCACGCGCAACGTCGTGTT

SmC4H1-hybrid-seq CGACATCTTCACCGGTAAAGGTCAGGACATGGTGTTCACCGTGTACGGCGAGCACTGGCGCAAGATGCGGCGGATCAT

SmC4H1-DQ355979.1 CGACATCTTCACCGGTAAAGGTCAGGACATGGTGTTCACCGTGTACGGCGAGCACTGGCGCAAGATGCGGCGGATCAT

SmC4H1-genome_annotation CGACATCTTCACCGGTAAAGGTCAGGACATGGTGTTCACCGTGTACGGCGAGCACTGGCGCAAGATGCGGCGGATCAT

SmC4H1-hybrid-seq GACGGTGCCGTTCTTCACCAACAAGGTGGTGCAGCAGTACCGCCATGGCTGGGAGGCAGAGGCCGCCGCCGTCGTGGA

SmC4H1-DQ355979.1 GACGGTGCCGTTCTTCACCAACAAGGTGGTGCAGCAGTACCGCCATGGCTGGGAGGCAGAGGCCGCCGCCGTCGTGGA

SmC4H1-genome_annotation GACGGTGCCGTTCTTCACCAACAAGGTGGTGCAGCAGTACCGCCATGGCTGGGAGGCAGAGGCCGCCGCCGTCGTGGA

SmC4H1-hybrid-seq GGACGTGAAGAAGAATCCGGAGTCGGCGACGAACGGGATCGTGCTGAGGCGGCGGCTGCAGCTGATGATGTACAACAA

SmC4H1-DQ355979.1 GGACGTGAAGAAGAATCCGGAGTCGGCGACGAACGGGATCGTGCTGAGGCGACGGCTGCAGCTGATGATGTACAACAA

SmC4H1-genome_annotation GGACGTGAAGAAGAATCCGGAGTCGGCGACGAACGGGATCGTGCTGAGGCGGCGGCTGCAGCTGATGATGTACAACAA

SmC4H1-hybrid-seq CATGTACCGGATCATGTTCGATAGAAGGTTCGAGAGCGAGGACGATCCTCTGTTTGTGAAATTGAAGGCGTTGAATGG

SmC4H1-DQ355979.1 CATGTACCGGATCATGTTCGATAGAAGGTTCGAGAGCGAGGACGATCCTCTGTTTGTGAAATTGAAGGCGTTGAATGG

SmC4H1-genome_annotation CATGTACCGGATCATGTTCGATAGAAGGTTCGAGAGCGAGGACGATCCTCTGTTTGTGAAATTGAAGGCGTTGAATGG

SmC4H1-hybrid-seq GGAGAGAAGCCGATTGGCGCAGAGCTTCGAATACAACTATGGCGATTTCATCCCAATTTTGAGGCCTTTCCTTAGAGG

SmC4H1-DQ355979.1 GGAGAGAAGCCGATTGGCGCAGAGCTTCGAATACAACTATGGCGATTTCATCCCAATTTTGAGGCCTTTCCTTAGAGG

SmC4H1-genome_annotation GGAGAGAAGCCGATTGGCGCAGAGCTTCGAATACAACTATGGCGATTTCATCCCAATTTTGAGGCCTTTCCTTAGAGG

SmC4H1-hybrid-seq TTACCTCAAGCTGTGCCAGCAGGTTAAGGAGAGAAGATTACAGCTGTTCAAAGACTATTTCGTTGATGAGAGAAAGAA

SmC4H1-DQ355979.1 TTACCTCAAGCTGTGCCAGCAGGTTAAGGAGAGAAGATTACAGCTGTTCAAAGACTATTTCGTTGATGAGAGAAAGAA

SmC4H1-genome_annotation TTACCTCAAGCTGTGCCAGCAGGTTAAGGAGAGAAGATTACAGCTGTTCAAAGACTATTTCGTTGATGAGAGAAAGAA

SmC4H1-hybrid-seq GCTGGTGAGCACAAAGGGGGGTGACAATGGCCTAAAGTGCGCGATCGATCACATGCTTGAAGCCCAGCAGAAGGGAGA

SmC4H1-DQ355979.1 GCTGGTGAGCACAAAGGGGGGTGACAATGGCCTAAAGTGCGCGATCGATCACATGCTTGAAGCCCAGCAGAAGGGAGA

SmC4H1-genome_annotation GCTGGTGAGCACAAAGGGGGGTGACAATGGCCTAAAGTGCGCGATCGATCACATGCTTGAAGCCCAGCAGAAGGGAGA

SmC4H1-hybrid-seq GATCAACGAGGATAACGTCCTTTACATTGTTGAGAATATTAATGTTGCTGCAATTGAGACAACTCTATGGTCAATTGA

SmC4H1-DQ355979.1 GATCAACGAGGATAACGTCCTTTACATTGTTGAGAATATTAATGTTGCTGCAATTGAGACAACTCTATGGTCAATTGA

SmC4H1-genome_annotation GATCAACGAGGATAACGTCCTTTACATTGTTGAGAATATTAATGTTGCTGCAATTGAGACAACTCTATGGTCAATTGA

SmC4H1-hybrid-seq GTGGGGCATTGCTGAGCTAGTGAACCACCCCGAGATCCAGAACAAGCTCCGACACGAACTCGACACGGTACTCGGCCC

SmC4H1-DQ355979.1 GTGGGGCATTGCTGAGCTAGTGAACCACCCCGAGATCCAGAACAAGCTCCGACACGAACTCGACACGGTACTCGGCCC

SmC4H1-genome_annotation GTGGGGCATTGCTGAGCTAGTGAACCACCCCGAGATCCAGAACAAGCTCCGACACGAACTCGACACGGTACTCGGCCC

```
SmC4H1-hybrid-seq          AGGAGTCCAAATAACAGAGCCGGATACTACCAAGCTCCCGTACCTTCAGGCTGTGGTCAAGGAGACCCTTCGTCTTCG
SmC4H1-DQ355979.1          AGGAGTCCAAATAACAGAGCCGGATACTACCAAGCTCCCGTACCTTCAGGCTGTGGTCAAGGAGACCCTTCGTCTTCG
SmC4H1-genome_annotation   AGGAGTCCAAATAACAGAGCCGGATACTACCAAGCTCCCGTACCTTCAGGCTGTGGTCAAGGAGACCCTTCGTCTTCG

SmC4H1-hybrid-seq          AATGGCCATCCCGCTACTAGTGCCCCACATGAACCTCCACGACGCGAAGCTCGGCGGCTTCGACATCCCCGCCGAGAG
SmC4H1-DQ355979.1          AATGGCCATCCCGCTACTAGTGCCCCACATGAACCTCCACGACGCGAAGCTTGGCGGCTTTGACATCCCTGCCGAGAG
SmC4H1-genome_annotation   AATGGCCATCCCGCTACTAGTGCCCCACATGAACCTCCACGACGCGAAGCTCGGCGGCTTCGACATCCCCGCCGAGAG

SmC4H1-hybrid-seq          TAAGATCTTGGTGAACGCTTGGTGGCTCGCCAACAACCCCGACCACTGGAAAAAGCCCGAAGAGTTTAGGCCCGAGAG
SmC4H1-DQ355979.1          TAAGATCTTGGTGAACGCTTGGTGGCTCGCCAACAACCCCGACCACTGGAAAAAGCCCGAAGAGTTTAGGCCCGAGAG
SmC4H1-genome_annotation   TAAGATCTTGGTGAACGCTTGGTGGCTCGCCAACAACCCCGACCACTGGAAAAAGCCCGAAGAGTTTAGGCCCGAGAG

SmC4H1-hybrid-seq          GTTCTTGGAGGAGGAGGCTAAAGTTGATGCCAACGGCAACGACTTTAGGTACCTCCCGTTCGGGGTTGGCCGGAGGAG
SmC4H1-DQ355979.1          GTTCTTGGAGGAGGAGGCTAAAGTTGATGCCAACGGCAACGACTTTAGGTACCTCCCGTTCGGGGTTGGCCGGAGGAG
SmC4H1-genome_annotation   GTTCTTGGAGGAGGAGGCTAAAGTTGATGCCAACGGCAACGACTTTAGGTACCTCCCGTTCGGGGTTGGCCGGAGGAG

SmC4H1-hybrid-seq          CTGCCCCGGGATCATTCTTGCGTTGCCTATTCTCGGCATCACGATAGGCCGTCTCGTGCAGAACTTCGAGCTGCTGCC
SmC4H1-DQ355979.1          CTGCCCCGGGATCATTCTTGCGTTGCCTATTCTCGGCATCACGATAGGCCGTCTCGTGCAGAACTTCGAGATGCTGCC
SmC4H1-genome_annotation   CTGCCCCGGGATCATTCTTGCGTTGCCTATTCTCGGCATCACGATAGGCCGTCTCGTGCAGAACTTCGAGCTGCTGCC

SmC4H1-hybrid-seq          TCCTCCGGGGCAGTCGAAGATCGACACATCGGAGAAGGGCGGGCAGTTCAGCCTCCACATTTTGAAGCACTCGACCAT
SmC4H1-DQ355979.1          TCCTCCGGGGCAGTCGAAGATCGACACATCGGAGAAGGGCGGGCAGTTCAGCCTCCACATTTTGAAGCACTCGACCAT
SmC4H1-genome_annotation   TCCTCCGGGGCAGTCGAAGATCGACACATCGGAGAAGGGCGGGCAGTTCAGCCTCCACATTTTGAAGCACTCGACCAT

SmC4H1-hybrid-seq          TGTTTTGAAGCCGAGATCATTTTGAAAAACACTAAAAAAAATCAATGGCTTTCTTTGAACATTTCTTGATTGTGTACT
SmC4H1-DQ355979.1          TGTTTTGAAGCCGAGATCATTTTGAAAAACACTAAAAAAAATCAATGGCTTTCTTTGAACATTTCTTGATTGTGTACT
SmC4H1-genome_annotation   TGTTTTGAAGCCGAGATCATTTTGA-----------------------------------------------------

SmC4H1-hybrid-seq          GAAAAATATTGAAGGGGCGAAGTTATGTTGTAATGATGATGTTTCTAAGTGCGATCGATCTTTGGATTTTACAAAATA
SmC4H1-DQ355979.1          GAAAAATATTGAAGGGGGGAAGTTATGTTGTAATGATGATGTTTTTAAGTGCGATCGATATTTGGATTTTACAAAATA
SmC4H1-genome_annotation   ------------------------------------------------------------------------------

SmC4H1-hybrid-seq          GTCTACGTTATGTTGTAATATTCATGTTTCTAATGTTACTCAATCTTTTGA---------------------------
SmC4H1-DQ355979.1          GTTTACGTTATGTTGTAATATTCATGTTTCTAATGTTACTCAATCTTTTGATTTGTCAAAAAAAAAAAAAAAAAAAAA
SmC4H1-genome_annotation   ------------------------------------------------------------------------------

SmC4H1-hybrid-seq          -----
SmC4H1-DQ355979.1          AAAAA
SmC4H1-genome_annotation   -----
```

#Sm4CL1

```
4CL1-hybrid-seq        --------------------AGAAAGTGAAGAAATGGAGTTGAAACAGGAAAACGAACACATCTTCCGTTCAAAGCTTCC
4CL1-AY237163.1        GTTGAGAAAACATTAGTTAGAGAAAGTGAAGAAATGGAGTTGAAACAGGAAAGCGAACACATCTTCCGTTCAAAGCTTCC
```

```
4CL1-genome_annotation ---------------------------------ATGGAGTTGAAACAGGAAAACGAACACATCTTCCGTTCAAAGCTTCC

4CL1-hybrid-seq        AGACATCAATATACCCACCCATCTCCCTCTACACACATATTGCTTCCAAAACCTCTCCACCCACCGGGCCCGCCCCTATC
4CL1-AY237163.1        AGACATCAATATACCCACCCATCTCCCTCTACACACATATTGCTTCCAAAACCTCTCCACCCACCGGGCCCGCCCCTATC
4CL1-genome_annotation AGACATCAATATACCCACCCATCTCCCTCTACACACATATTGCTTCCAAAACCTCTCCACCCACCGGGCCCGCCCCTATC

4CL1-hybrid-seq        TAATCAACGCCGCCACCGGCGATACCTTCACCCATGCCGGATTCGAGCTCACCGCGCGCAGAGTCGCCGCCGGCCTCCAC
4CL1-AY237163.1        TAATCAACGCCGCCACCGGCGATACCTTCACCCATGCCGGATTCGAGCTCACCGCGCGCAGAGTCGCCGCCGGCCTCCAC
4CL1-genome_annotation TAATCAACGCCGCCACCGGCGATACCTTCACCCATGCCGGATTCGAGCTCACCGCGCGCAGAGTCGCCGCCGGCCTCCAC

4CL1-hybrid-seq        AACCTCGGCATCCGAAAATCGGACGTCGTCATGCTTCTCCTCCACAACTCGCCCGAATTCGCATTCGCATTTCTCGGCGC
4CL1-AY237163.1        AACCTCGGCATCCGAAAATCGGACGTCGTCACGCTTCTCCTCCACAACTCGCCCGAATTCGCATTCGCATTTCTCGGCGC
4CL1-genome_annotation AACCTCGGCATCCGAAAATCGGACGTCGTCATGCTTCTCCTCCACAACTCGCCCGAATTCGCATTCGCATTTCTCGGCGC

4CL1-hybrid-seq        CTCCTTCATCGGCGCGATCTCCACCACCGCCAATCCTCTCTACACCGCCTCGGAGATCGCGCTTCAGGCTAGAATCTCCA
4CL1-AY237163.1        CTCCTTCATCGGCGCGATCTCCACCACCGCCAATCCTCTCTACACCGCATCGGAGATCGCGCTTCAGGCTAGAATCTCCA
4CL1-genome_annotation CTCCTTCATCGGCGCGATCTCCACCACCGCCAATCCTCTCTACACCGCCTCGGAGATCGCGCTTCAGGCTAGAATCTCCA

4CL1-hybrid-seq        GACCTAAGCTCATAGTCACCCACGCTTGCCACGTGGAAAAGGTGAAGCACTACGCCGCCGAGGCCGGCGCCAAAATCGCC
4CL1-AY237163.1        GACCTAAGCTCATAGTCACCCACGCTTGCCACGTGGAAAAGGTGAAGCACTACGCCGCCGAGGCCGGCGCCAAAATCGCC
4CL1-genome_annotation GACCTAAGCTCATAGTCACCCACGCTTGCCACGTGGAAAAGGTGAAGCACTACGCCGCCGAGGCCGGCGCCAAAATCGCC

4CL1-hybrid-seq        ACGATCGACCCCCCGCCCTCGCCGGAGATCATCCACTTCGCCGAACTGAGGAGATCCGACGAGAAGCTGCTCACGCCGGT
4CL1-AY237163.1        ACGATCGACCCCCCGCCCTCGCCGGAGATCATCCACTTCGCCGAACTGAGGAGATCCGACGAGAAGCTGCTCACGCCGAT
4CL1-genome_annotation ACGATCGACCCCCCGCCCTCGCCGGAGATCATCCACTTCGCCGAACTGAGGAGATCCGACGAGAAGCTGCTCACGCCGGT

4CL1-hybrid-seq        CGAGATCCACGCCGACGACACGGTGGCGCTGCCATTCTCCTCCGGCACCACCGGGCCTCCACCAAGGGAGTAATGCTGAG
4CL1-AY237163.1        CGAGATCCACGCCGACGACACGGTGGCGCTGCCATTCTCCTCCGGCACCACC-GGCCTCC-CCAAGGGAGTAATGCTGAG
4CL1-genome_annotation CGAGATCCACGCCGACGACACGGTGGCGCTGCCATTCTCCTCCGGCACCACC-GGCCTCC-CCAAGGGAGTAATGCTGAG

4CL1-hybrid-seq        CCACAAAAACCTAGTCGCGTGCGTGTCGCAGCAAGTGGACGGCGAGAATCCGGCAGTTCACATCGATCGTGAGGATCGGA
4CL1-AY237163.1        CCACAAAAACCTAGTCGCGTGCGTGTCGCAGCAAGTGGACGGCGAGAATCCGGCAGTTCACATCGATCGTGAGGATCGGA
4CL1-genome_annotation CCACAAAAACCTAGTCGCGTGCGTGTCGCAGCAAGTGGACGGCGAGAATCCGGCAGTTCACATCGATCGTGAGGATCGGA

4CL1-hybrid-seq        TGCTCTGCGTGTTGCCGCTGTTCCACGTGTACAGTATGATCTCGGTGATGCTCTGCTGCCTGCGTGTCGGTGCGGCCGTG
4CL1-AY237163.1        TGCTCTGCGTGTTGCCGCTGTTCCACGTGTACAGTATGATCTCGGTGATGCTCTGCTGTCTGCGTGTCGGTGCGGCCGTG
4CL1-genome_annotation TGCTCTGCGTGTTGCCGCTGTTCCACGTGTACAGTATGATCTCGGTGATGCTCTGCTGCCTGCGTGTCGGTGCGGCCGTG

4CL1-hybrid-seq        GTGTTTATGCCTAAGTTTGAAATCAGTGAGTTAATGGAGTTAATAGAGAAATACAGGGTGACGATTGCACCATTTGTGCC
4CL1-AY237163.1        GTGATTATGCCTAAGTTTGAAATCAGTGAGTTAATGGAGTTAATAGAGAAATACAGGGTGACGATTGCACCATTTGTGCC
4CL1-genome_annotation GTGATTATGCCTAAGTTTGAAATCAGTGAGTTAATGGAGTTAATAGAGAAATACAGGGTGACGATTGCACCATTTGTGCC

4CL1-hybrid-seq        ACCGATATTGCTGGCGATTGCGAAGAGCCCGGCGGCGGCGAAGTTCGATTTTTCGTCGGTCAGGAGAGTCGTCTGCGGCG
4CL1-AY237163.1        ACCGATATTGCTGGCGATTGCGAAGAGCCCGGCGGCGGCGAAGTTCGATTTTTCGTCGGTGAGGAGAGTCGTCTGCGGCG
```

```
4CL1-genome_annotation ACCGATATTGCTGGCGATTGCGAAGAGCCCGGCGGCGGCGAAGTTCGATTTTTCGTCGGTCAGGAGAGTCGTCTGCGGCG

4CL1-hybrid-seq        CGGCGGCACCCATGGATAGAGAACTTGAGCTAGCACTCAAAGCGAAGCTCCCTAATGCCGTTATTGGCCAGGGTTATGGT
4CL1-AY237163.1        CGGCG---CCCATGGATAGAGAACTTGAGCTAGCACTCAAAGCGAAGCTCCCTAATGCCGTTATTGGCCAGGGTTATGGT
4CL1-genome_annotation CGGCG---CCCATGGATAGAGAACTTGAGCTAGCACTCAAAGCGAAGCTCCCTAATGCCGTTATTGGCCAGGGTTATGGT

4CL1-hybrid-seq        ATGACAGAAGCTGGAGTATTATCGATGAGCTTAGGGTTCGCGAAGAGGCCATTAAAATTCAAAGCTGGTTCATGTGGAAC
4CL1-AY237163.1        ATGACAGAAGCTGGAGTATTATCGATGAGCTTAGGGTTCGCGAAGAGGCCATTAAAATTCAAAGCTGGTTCATGTGGAAC
4CL1-genome_annotation ATGACAGAAGCTGGAGTATTATCGATGAGCTTAGGGTTCGCGAAGAGGCCATTAAAATTCAAAGCTGGTTCATGTGGAAC

4CL1-hybrid-seq        TGTGATTAGAAACGCGCGGATGAAGATCGTGGACCCCTCCAGCGCCGCCTCTCTTCCTCGGAATCAGACCGGGGAGATCT
4CL1-AY237163.1        TGTGATTAGAAACGCGCGGATGAAGATCGTGGACCCCTCCAGCGCCGCCTCTCTTCCTCGGAATGAGACCGGGGAGATCT
4CL1-genome_annotation TGTGATTAGAAACGCGCGGATGAAGATCGTGGACCCCTCCAGCGCCGCCTCTCTTCCTCGGAATCAGACCGGGGAGATCT

4CL1-hybrid-seq        GCATCAAGGGAGACGCTGTGATGAAAGGGTATTACAATGATCCGGAGGCGACGAGGAGGACTATTGATGAGGAGGGGTGG
4CL1-AY237163.1        GCATCAAGGGAGACGCTGTGATGAAAGGGTATTACAATGATCCGGAGGCGACGAGGAGGACTATTGATGAGGAGGGGTGG
4CL1-genome_annotation GCATCAAGGGAGACGCTGTGATGAAAGGGTATTACAATGATCCGGAGGCGACGAGGAGGACTATTGATGAGGAGGGGTGG

4CL1-hybrid-seq        CTGCACACGGGCGATTTAGGGTTCGTGGACGATGATGAGGAAGTGTACATCGTCGACAGGTTGAAGGAATTGATCAAATA
4CL1-AY237163.1        CTGCACACGGGCGATTTAGGGTTCGTGGACGATGATGAGGAAGTGTACATCGTCGACAGGTTGAAGGAATTGATCAAATA
4CL1-genome_annotation CTGCACACGGGCGATTTAGGGTTCGTGGACGATGATGAGGAAGTGTACATCGTGGACAGGTTGAAGGAATTGATCAAATA

4CL1-hybrid-seq        CAAAGGCTTTCATATTGCTCCTGCTGAACTTGAAGCTTTGCTCGTCGCACATCCCTCTATATCTGAAGCTGCCGTTGTGC
4CL1-AY237163.1        CAAAGGCTTTCATATTGCCCCTGCTGAACTTGAAGCTTTGCTCGTCGCACATCCCTCTATATCTGAAGCTGCCGTTGTGC
4CL1-genome_annotation CAAAGGCTTTCATATTGCTCCTGCTGAACTTGAAGCTTTGCTCGTCGCACATCCCTCTATATCTGAAGCTGCCGTTGTGC

4CL1-hybrid-seq        CTATGGCGGATGAGGCTGCTGGAGAAGTTCCAGTTGCATTTGTAGTGCGAGCAAATGCTGCATATATTACCGAGCTAGAG
4CL1-AY237163.1        CTATGGCGGATGAGGCTGCTGGAGAAGTTCCAGTTGCATTTGTAGTGCGAGCAAATGCTGCATATATTACCGAGCTACAG
4CL1-genome_annotation CTATGGCGGATGAGGCTGCTGGAGAAGTTCCAGTTGCATTTGTAGTGCGAGCAAATGCTGCATATATTACCGAGCTAGAG

4CL1-hybrid-seq        ATCAAGAGGTATATTAGCGAACCGACGTGCGCGCGTACAAAC--CTCAGATCGTGTGATTGTTA-CGATGCAATTCCTAA
4CL1-AY237163.1        ATCAAGAGATATA-TAGCGAACCAGGTGGCGC-CGTACAAGCGGATCA-ATCGTGTATTTTTTACCGATACAATTCCTAA
4CL1-genome_annotation ATCAAGAGATATA-TAGCGAACCAGGTGGCGC-CGTACAAGCGGATCA-ATCGTGTATTTTTTACCGATACAATTCCTAA

4CL1-hybrid-seq        AGCCCCCACGGGTAAAATTTTAAGAAAAGATCTACGAGCTAGACTTTAATTTCAATGGAGCTACTCAAGTAGTATTAGAC
4CL1-AY237163.1        AGCCCCCACGGGTAAAATTTTAAGAAAAGATCTACGAGCTAGACTTTAATTTCAATGGAGCTACTCAAGTAGTATTAGAC
4CL1-genome_annotation AGCCCCCACGGGTAAAATTTTAAGAAAAGATCTACGAGCTAGACTTTAA-------------------------------

4CL1-hybrid-seq        TAACATCAAATAAATTGACATTTGGAACGA--------------------------------------------------
4CL1-AY237163.1        TAACATCAAATAAATTGACATTTGGAACGAGGATATTTATATTTTCAATTTTAAGCTCGATGTTAGTAGAAAAAAAAAAA
4CL1-genome_annotation --------------------------------------------------------------------------------

4CL1-hybrid-seq        --------
4CL1-AY237163.1        AAAAAAAA
```

4CL1-genome_annotation --------

#Sm4CL2

4CL2-hybrid-seq ---TCCAATTCCCTTCCCAAAAAAGAAAAAAGAAAA

4CL2-AY237164.1 GTCTCAGGTTCAACTCTACAAAACTAAAGTTGATCCAAAAAAAATTCCAATTCCCTTCCCAAAAAAGAAAAAAGAAAA

4CL2-genome_annotation --

4CL2-hybrid-seq AAAATCCACTTCATTTCCAGCAATGGAGGTTCCCACGATGCCGGAAGAGATCGTATTCCGATCCAAGCTCCCCGACAT

4CL2-AY237164.1 AAAATCCACTTCATTTCCAGCAATGGAGGTTCCCACGATGCCGGAAGAGATCGTATTCCGATCCAAGCTCCCCGACAT

4CL2-genome_annotation ----------------------ATGGAGGTTCCCACGATGCCGGAAGAGATCGTATTCCGATCCAAGCTCCCCGACAT

4CL2-hybrid-seq ATACATTCCGAAGCATCTCCCGTTACACTCATACTGCTTCGAGAACATCTCCAAGTTCAGCTCGCGGCCGTGCATTAT

4CL2-AY237164.1 ATACATTCCGAAGCATCTCCCGTTACACTCATACTGCTTCGAGAACATCTCCAAGTTCAGCTCGCGGCCGTGCATTAT

4CL2-genome_annotation ATACATTCCGAAGCATCTCCCGTTACACTCATACTGCTTCGAGAACATCTCCAAGTTCAGCTCGCGGCCGTGCATTAT

4CL2-hybrid-seq CAACGGCGCGACGGGCGACGTGTACACGTACGAGGAGGTGGAGATGACGGCGCGCAAGGTGGCGTCGGGGCTGAGCCA

4CL2-AY237164.1 CAACGGCGCGACGGGCGACGTGTACACGTACGAGGAGGTGGAGATGACGGCGCGCAAGGTGGCGTCGGGGCTGAGCCA

4CL2-genome_annotation CAACGGCGCGACGGGCGACGTGTACACGTACGAGGAGGTGGAGATGACGGCGCGCAAGGTGGCGTCGGGGCTGAGCCA

4CL2-hybrid-seq GGTGGGCATCCAGCAGGGCGAGACGATCATGCTCCTCCTCCCCAACACGCCCGAGTACATCTTCGCATTCCTCGGCGC

4CL2-AY237164.1 GGTGGGCATCCAGCAGGGCGAGACGATCATGCTCCTCCTCCCCAACACGCCCGAGTACATCTTCGCATTCCTCGGCGC

4CL2-genome_annotation GGTGGGCATCCAGCAGGGCGAGACGATCATGCTCCTCCTCCCCAACACGCCCGAGTACATCTTCGCATTCCTCGGCGC

4CL2-hybrid-seq GTCCTACATAGGCGCGGTCTCCACCATGGCGAATCCCTTCTTCACCCCGGCCGAGGTGATCAAGCAGGCCAAGGCCTC

4CL2-AY237164.1 GTCCTACATAGGCGCGGTCTCCACCATGGCGAATCCCTTCTTCACCCCGGCCGAGGTGATCAAGCAGGCCAAGGCCTC

4CL2-genome_annotation GTCCTACATAGGCGCGGTCTCCACCATGGCGAATCCCTTCTTCACCCCGGCCGAGGTGATCAAGCAGGCCAAGGCCTC

4CL2-hybrid-seq CGCGGCCAAGCTCATCATCACGCAGGCGTGCTACGTGGACAAGGTGCGCGACTACGCGGCGGAGGCCGGGGCGAAGGT

4CL2-AY237164.1 CGCGGCCAAGCTCATCATCACGCAGGCGTGCTACGTGGACAAGGTGCGCGACTACGCGGCGGAGGCCGGGGCGAAGGT

4CL2-genome_annotation CGCGGCCAAGCTCATCATCACGCAGGCGTGCTACGTGGACAAGGTGCGCGACTACGCGGCGGAGGCCGGGGCGAAGGT

4CL2-hybrid-seq GGTGTGCATCGACGCGCCGCCGGCCGGGTGCCTGACGTTCTCGGAGCTGACGGCGGCGGACGAGCGGGAGATGCCGGC

4CL2-AY237164.1 GGTGTGCATCGACGCGCCGCCGGCCGGGTGCCTGACGTTCTCGGAGCTGACGGCGGCGGACGAGCGGGAGATGCCGGC

4CL2-genome_annotation GGTGTGCATCGACGCGCCGCCGGCCGGGTGCCTGACGTTCTCGGAGCTGACGGCGGCGGACGAGCGGGAGATGCCGGC

4CL2-hybrid-seq GGTGAAGATACACCCGGAGGACGCGGTGGCGCTGCCGTATTCGTCAGGGACGACGGGGCTGCCGAAGGGGGTGATGCT

4CL2-AY237164.1 GGTGAAGATACACCCGGAGGACGCGGTGGCGCTGCCGTATTCGTCAGGGACGACGGGGCTGCCGAAGGGGGTGATGCT

4CL2-genome_annotation GGTGAAGATACACCCGGAGGACGCGGTGGCGCTGCCGTATTCGTCAGGGACGACGGGGCTGCCGAAGGGGGTGATGCT

4CL2-hybrid-seq GACGCACAAGGGGCTGGTGACGAGCGTGGCGCAGCAGGTGGACGGGGAGAACCCGAATTTGTATATCCATAGCGAGGA

4CL2-AY237164.1 GACGCACAAGGGGCTGGTGACGAGCGTGGCGCAGCAGGTGGACGGGGAGAACCCGAATTTGTATATCCATAGCGAGGA

4CL2-genome_annotation GACGCACAAGGGGCTGGTGACGAGCGTGGCGCAGCAGGTGGACGGGGAGAACCCGAATTTGTATATCCATAGCGAGGA

4CL2-hybrid-seq CGTGATGCTGTGCGTGCTGCCGCTGTTTCATATATACTCGTTGAACTCGGTTTTGCTGTGCGGGCTGCGGGTCGGGGC

4CL2-AY237164.1 CGTGATGCTGTGCGTGCTGCCGCTGTTTCATATATACTCGTTGAACTCGGTTTTGCTGTGCGGGCTGCGGGTCGGGGC
4CL2-genome_annotation CGTGATGCTGTGCGTGCTGCCGCTGTTTCATATATACTCGTTGAACTCGGTTTTGCTGTGCGGGCTGCGGGTCGGGGC

4CL2-hybrid-seq GGCCATCCTCATCATGCAGAAGTTCGAGATCGTGCCGTTTCTCGACCTCATGCAGAGGTACAAAGTCACGATTGGGCC
4CL2-AY237164.1 GGCCATCCTCATCATGCAGAAGTTCGAGATCGTGCCGTTTCTCGACCTCATGCAGAGGTACAAAGTCACGATTGGGCC
4CL2-genome_annotation GGCCATCCTCATCATGCAGAAGTTCGAGATCGTGCCGTTTCTCGACCTCATGCAGAGGTACAAAGTCACGATTGGGCC

4CL2-hybrid-seq CTTCGTGCCGCCCATAGTGCTGGCCATCGCCAAGAGCCCGCTCGTCGCCAAATACGACCTTTCCTCCGTGCGGATGGT
4CL2-AY237164.1 CTTCGTGCCGCCCATAGTGCTGGCCATCGCCAAGAGCCCGCTCGTCGCCAAATACGACCTTTCCTCCGTGCGGATGGT
4CL2-genome_annotation CTTCGTGCCGCCCATAGTGCTGGCCATCGCCAAGAGCCCGCTCGTCGCCAAATACGACCTTTCCTCCGTGCGGATGGT

4CL2-hybrid-seq CATGTCCGGCGCCGCGCCGCTCGGGAAGGAGCTCGAGGATTCTGTCAGGACCAAGTTTCCTAATGCAAAACTTGGACA
4CL2-AY237164.1 CATGTCCGGCGCCGCGCCGCTCGGGAAGGAGCTCGAGGATTCTGTCAGGACCAAGTTTCCTAATGCAAAACTTGGACA
4CL2-genome_annotation CATGTCCGGCGCCGCGCCGCTCGGGAAGGAGCTCGAGGATTCTGTCAGGACCAAGTTTCCTAATGCAAAACTTGGACA

4CL2-hybrid-seq -------GGGATGACTGAAGCAGGGCCAGTGCTATCGATGTGCCTAGCGTTTGCAAAAGAGCCATTTGAGATAAAATC
4CL2-AY237164.1 GGGGTATGGGATGACTGAAGCAGGGCCTGTGCTATCGATGTGCCTAGCGTTTGCAAAAGAGCCATTTGAGATAAAATC
4CL2-genome_annotation GGGGTATGGGATGACTGAAGCAGGGCCAGTGCTATCGATGTGCCTAGCGTTTGCAAAAGAGCCATTTGAGATAAAATC

4CL2-hybrid-seq AGGAGCATGTGGAACTGTTGTTAGAAATGCTGAAATGAAAATCATTGATCCTCAAACTGGTGTTTCTCTTGGGCGTAA
4CL2-AY237164.1 AGGAGCATGTGGAACTGTTGTTAGAAATGCTGAAATGAAAATCATTGATCCTCAAACTGGTGTTTCTCTTGGGCGTAA
4CL2-genome_annotation AGGAGCATGTGGAACTGTTGTTAGAAATGCTGAAATGAAAATCATTGATCCTCAAACTGGTGTTTCTCTTGGGCGTAA

4CL2-hybrid-seq TCAATCTGGAGAGATTTGCATTAGAGGAGACCAGATTACGAAAGGTTATCTGAACGACCCAGAGTCAACAAAGAACAC
4CL2-AY237164.1 TCAATCCGGAGAGATTTGCATTAGAGGAGACCAGATTATGAAAGGTTATCTGAACGACCCAGAGTCAACAAAGAACAC
4CL2-genome_annotation TCAATCCGGAGAGATTTGCATTAGAGGAGACCAGATTATGATAGGTTATCTGAACGACCCAGAGTCAACAAAGAACAC

4CL2-hybrid-seq CATAGACGAAGACGGGTGGCTGCACACTGGCGACATCGGATTCATTGATGCCGACGACGAGCTCTTCATCGTGGACCG
4CL2-AY237164.1 CATAGACGAAGACGGGTGGCTGCACACTGGCGACATCGGATTCATTGATGCCGACGACGAGCTCTTCATCGTGGACCG
4CL2-genome_annotation CATAGACGAAGACGGGTGGCTGCACACTGGCGACATCGGATTCATTGATGCCGACGACGAGCTCTTCATCGTGGACCG

4CL2-hybrid-seq ATTGAAGGAAATTATAAAATACAAAGGGTTCCAAGTTGCGCCAGCTGAAATCGAAGCCCTCCTCCTCAATAATCCATA
4CL2-AY237164.1 ATTGAAGGAAATTATAAAATACAAAGGGTTCCAAGTTGCGCCAGCTGAAATCGAAGCCCTCCTCCTCAATAATCCATA
4CL2-genome_annotation ATTGAAGGAAATTATAAAATACAAAGGGTTCCAAGTTGCGCCAGCTGAAATCGAAGCCCTCCTCCTCAATAATCCATA

4CL2-hybrid-seq CATCTCCGACGCTGCAGTTGTCTCAATGCAAGATGAGCAAGCTGGAGAAGTCCCAGTTGCTTTCGTGGTGAGATCGAA
4CL2-AY237164.1 CATCTCCGACGCTGCAGTTGTCTCAATGCAAGATGAGCAAGCTGGAGAAGTCCCAGTTGCTTTCGTCGTGAGATCGAA
4CL2-genome_annotation CATCTCCGACGCTGCAGTTGTCAGAATGCAAGATGAGCAAGCTGGAGAAGTCCCAGTTGCTTTCGTGGTGAGATCGAA

4CL2-hybrid-seq TGGTTCTACAATCACTGAGGATGAAATCAAGCAATTTATCTCGAAACAGGTTATCTTCTACAAGAGAATAAATCGTGT
4CL2-AY237164.1 TGGTTCTACAATCACTGAGGATGAAATCAAGCAATTTATCTCGAAACAGGTTATCTTCTACAAGAGAATAAATCGTGT
4CL2-genome_annotation TGGTTCTACAATCACTGAGGATGAAATCAAGCAATTTATCTCGAAACAGGTTATCTTCTACAAGAGAATAAATCGTGT

4CL2-hybrid-seq ATTTTTCATTGATGCAATTCCCAAGTCTCCATCAGGAAAAATATTGAGAAAGGATTTGAGAGCAAGATTAGCAGCTGC

```
4CL2-AY237164.1         ATTTTTCATTGATGCAATTCCCAAGTCTCCATCAGGAAAAATATTGAGAAAGGATTTGAGAGCAAGATTAGCAGCTGC
4CL2-genome_annotation  ATTTTTCATTGATGCAATTCCCAAGTCTCCATCAGGAAAAATATTGAGAAAGGATTTGAGAGCAAGATTAGCAGCTGC

4CL2-hybrid-seq         AGTCTAAAATTATCAATGAAAAAGATCTATCCATTATATAATTTCTTTCAGCCTTGATTTTTTTTTAATTCACACAA
4CL2-AY237164.1         AGTCTAAAATTATCAATGAAAAAGATCTATCCATTATATAATTTCTTTCAGCCTTGATTTTTTTTTAATTCACACAA
4CL2-genome_annotation  AGTCTAA-------------------------------------------------------------------------

4CL2-hybrid-seq         TATGGAAATTTAAA------------------------------------------------------------------
4CL2-AY237164.1         TATGGAAATTTAAAGACCAATGCTTGTATATTACATGTGGATGTTGAGGTTTAAAAAAAAAAAAAAA
```

#Sm4CL3

```
4CL3-hybrid-seq         TTTCCTCAAACAGAACTGCAACTTCTCTCTCTCTCTCTCTCTCTCCCTCTCTCCCTCTCTCTCAATTCACTAACGCAA
4CL3-KF220556           --------------------------------------------------------------------------------
4CL3-genome_annotation  --------------------------------------------------------------------------------

4CL3-hybrid-seq         ACGGAGTAGCTAGCAGTTAATCGATCAACGATGTTGTCGGTGGCAGCCGCCGAAGCTCAGAATCCAGAGCTCTCATCT
4CL3-KF220556           ------------------------------ATGTTGTCGGTGGCAGCCGCCGAAGCTCAGAATCCAGAGCTCTCATCT
4CL3-genome_annotation  ------------------------------ATGTTGTCGGTGGCAGCCGCCGAAGCTCAGAATCCAGAGCTCTCATCT

4CL3-hybrid-seq         CACGCGCTGCCGCAAACTCGATCACCGGAAGAAACTCATATCTTCGTTTCCAAACTCCCCACCATTCCCATCTCCAAT
4CL3-KF220556           CACGCGCTGCCGCAAACTCGATCACCGGAAGAAACTCATATCTTCGTTTCCAAACTCCCCACCATTCCCATCTCCAAT
4CL3-genome_annotation  CACGCGCTGCCGCAAACTCGATCACCGGAAGAAACTCATATCTTCGTTTCCAAACTCCCCACCATTCCCATCTCCAAT

4CL3-hybrid-seq         GATATCCCACTCCACACATACTGCTTCCAGAATTACGCGCAGTACCCCGATCGCCCCTGCCTCATCGTCGGCACCGGC
4CL3-KF220556           GATATCCCACTCCACACATACTGCTTCCAGAATTACGCGCAGTACCCCGATCGCCCCTGCCTCATCGTCGGCACCGGC
4CL3-genome_annotation  GATATCCCACTCCACACATACTGCTTCCAGAATTACGCGCAGTACCCCGATCGCCCCTGCCTCATCGTCGGCACCGGC

4CL3-hybrid-seq         GCCGGAAAATCCTACACCTTCGCCGAAACGCACCTCATCTGCCGGAAGGTCGCCGCCGGCTTCTCCAATCGCGGAATT
4CL3-KF220556           GCCGGAAAATCCTACACCTTCGCCGAAACGCACCTCATCTGCCGGAAGGTCGCCGCCGGCTTCTCCAATCGCGGAATT
4CL3-genome_annotation  GCCGGAAAATCCTACACCTTCGCCGAAACGCACCTCATCTGCCGGAAGGTCGCCGCCGGCTTCTCCAATCGCGGAATT

4CL3-hybrid-seq         AAGAAAGGCGATGTGATCATGGTGCTCCTCCAGAACTGCGCCGAATTCGTCTTCACGTTCATGGGGGCTTCCATGATC
4CL3-KF220556           AAGAAAGGCGATGTGATCATGGTGCTCCTCCAGAACTGCGCCGAATTCGTCTTCACGTTCATGGGGGCTTCCATGATC
4CL3-genome_annotation  AAGAAAGGCGATGTGATCATGGTGCTCCTCCAGAACTGCGCCGAATTCGTCTTCACGTTCATGGGGGCTTCCATGATC

4CL3-hybrid-seq         GGCGCCGTCACCACCACCGCCAATCCTTTCTGCACCTCCAATGAGATCTTTAAGCAGTTCAAGGCCTCCAAATCGAAG
4CL3-KF220556           GGCGCCGTCACCACCACCGCCAATCCTTTCTGCACCTCCAATGAGATCTTTAAGCAGTTCAAGGCCTCCAAATCGAAG
4CL3-genome_annotation  GGCGCCGTCACCACCACCGCCAATCCTTTCTGCACCTCCAATGAGATCTTTAAGCAGTTCAAGGCCTCCAAATCGAAG

4CL3-hybrid-seq         ATGATCGTCACGCAGGCTATGTACGTCGACAAGCTCCGCGACACCGGCGACGAGTCCATCGTATTCGGCGAGGATTTC
4CL3-KF220556           ATGATCGTCACGCAGGCTATGTACGTCGACAAGCTCCGCGACACCGGCGACGAGTCCATCGTATTCGGCGAGGATTTC
4CL3-genome_annotation  ATGATCGTCACGCAGGCTATGTACGTCGACAAGCTCCGCGACACCGGCGACGAGTCCATCGTATTCGGCGAGGATTTC

4CL3-hybrid-seq         TCCGTCGTCACCATCGACGCCCCGCCGGAGGGATGCCTGCCTTTCTCCGCGCTCTCGGAGGCCGACGAGGCCACCGCG
```

```
4CL3-KF220556           TCCGTCGTCACCATCGACGCCCCGCCGGAGGGATGCCTGCCTTTCTCCGCGCTCTCGGAGGCCGACGAGGCCACCGCG
4CL3-genome_annotation  TCCGTCGTCACCATCGACGCCCCGCCGGAGGGATGCCTGCCTTTCTCCGCGCTCTCGGAGGCCGACGAGGCCACCGCG

4CL3-hybrid-seq         CCGGAGGTCGAGATCGATCCGAACGACGCCGTCGCACTGCCGTTCTCCTCCGGGACCACCGGACTGCCCAAGGGCGTG
4CL3-KF220556           CCGGAGGTCGAGATCGATCCGAACGACGCCGTCGCACTGCCGTTCTCCTCCGGGACCACCGGACTGCCCAAGGGCGTG
4CL3-genome_annotation  CCGGAGGTCGAGATCGATCCGAACGACGCCGTCGCACTGCCGTTCTCCTCCGGGACCACCGGACTGCCCAAGGGCGTG

4CL3-hybrid-seq         ATCCTCACGCACAAGAGCTTGATCACCAGCATCGCGCAGCAGGTCGACGGCGACAACCCTAATTTATACCTCAAGCCG
4CL3-KF220556           ATCCTCACGCACAAGAGCTTGATCACCAGCATCGCGCAGCAGGTCGACGGCGACAACCCTAATTTATACCTCAAGCCG
4CL3-genome_annotation  ATCCTCACGCACAAGAGCTTGATCACCAGCATCGCGCAGCAGGTCGACGGCGACAACCCTAATTTATACCTCAAGCCG

4CL3-hybrid-seq         GACGACGTCGTTCTCTGCGTGCTGCCGCTCTTCCACATCTACTCCTTGAATTCGGTGCTTCTCTGCTCGCTCAGAGCC
4CL3-KF220556           GACGACGTCGTTCTCTGCGTGCTGCCGCTCTTCCACATCTACTCCTTGAATTCGGTGCTTCTCTGCTCGCTCAGAGCC
4CL3-genome_annotation  GACGACGTCGTTCTCTGCGTGCTGCCGCTCTTCCACATCTACTCCTTGAATTCGGTGCTTCTCTGCTCGCTCAGAGCC

4CL3-hybrid-seq         GGCGCCGGCGTTCTGCTTATGCAGAAATTTGAGATCGGCTCGCTGCTGGAGCTGATTCAGCGGCACCGCGTCTCGGTG
4CL3-KF220556           GGCGCCGGCGTTCTGCTTATGCAGAAATTTGAGATCGGCTCGCTGCTGGAGCTGATTCAGCGGCACCGCGTCTCGGTG
4CL3-genome_annotation  GGCGCCGGCGTTCTGCTTATGCAGAAATTTGAGATCGGCTCGCTGCTGGAGCTGATTCAGCGGCACCGCGTCTCGGTG

4CL3-hybrid-seq         GCGGCGGTGGTGCCGCCGCTTGTGCTGGCGCTGGCGAAGAATCCGCTTGTGGACAACTTCGATTTGAGCTCGATCAGG
4CL3-KF220556           GCGGCGGTGGTGCCGCCGCTTGTGCTGGCGCTGGCGAAGAATCCGCTGGTGGACAACTTCGATTTGAGCTCGATCAGG
4CL3-genome_annotation  GCGGCGGTGGTGCCGCCGCTTGTGCTGGCGCTGGCGAAGAATCCGCTGGTGGACAACTTCGATTTGAGCTCGATCAGG

4CL3-hybrid-seq         ATGGTGCTGTCCGGGGCGGCGCCGCTGGGGAAGGAGCTGGAGACGGCGTTGCTCAGCCGTCTGCCGCAGGCAGTTTTT
4CL3-KF220556           ATGGTGCTGTCCGGGGCGGCGCCGCTGGGGAAGGAGCTGGAGACGGCGTTGCTCAGCCGTCTGCCGCAGGCAGTTTTT
4CL3-genome_annotation  ATGGTGCTGTCCGGGGCGGCGCCGCTGGGGAAGGAGCTGGAGACGGCGTTGCTCAGCCGTCTGCCGCAGGCAGTTTTT

4CL3-hybrid-seq         GGACAGGGTTATGGCATGACTGAGGCCGGGCCGGTATTATCTATGTCCCCGTCGTTCGCGAAGCAGCCACTACCAACC
4CL3-KF220556           GGACAGGGTTATGGCATGACTGAGGCCGGGCCGGTATTATCTATGTCCCCGTCGTTCGCGAAGCAGCCACTACCAACC
4CL3-genome_annotation  GGACAGGGTTATGGCATGACTGAGGCCGGGCCGGTATTATCTATGTCCCCGTCGTTCGCGAAGCAGCCACTACCAACC

4CL3-hybrid-seq         AAGTCCGGCTCCTGCGGCAATGTCGTCCGCAATGCCGAGCTCAAGGTCGTCGACCCCGACACCGGCTGCTCCCTCCCC
4CL3-KF220556           AAGTCCGGCTCCTGCGGCAATGTCGTCCGCAATGCCGAGCTCAAGGTCGTCGACCCCGACACCGGCTGCTCCCTCCCC
4CL3-genome_annotation  AAGTCCGGCTCCTGCGGCAATGTCGTCCGCAATGCCGAGCTCAAGGTCGTCGACCCCGACACCGGCTGCTCCCTCCCC

4CL3-hybrid-seq         CGCAACCAACCGGGCGAAATTTGTATTCGTGGACCCCAGATCATGAAAGGGTACTTGAATGATGCGGAGGCGACTGCT
4CL3-KF220556           CGCAACCAACCGGGCGAAATTTGTATTCGTGGACCCCAGATCATGAAAGGGTACTTGAATGATGCGGAGGCGACTGCT
4CL3-genome_annotation  CGCAACCAACCGGGCGAAATTTGTATTCGTGGACCCCAGATCATGAAAGGGTACTTGAATGATGCGGAGGCGACTGCT

4CL3-hybrid-seq         AGAACCGTAGACGTGGACGGTTGGCTCCACACC---GACATCGGATATGTGGACGACGACGACGATGTTTTCATTGTA
4CL3-KF220556           AGAACCGTAGACGTGGACGGTTGGCTCCACACCGGTGACATCGGATATGTGGACGACGACGACGATGTTTTCATTGTA
4CL3-genome_annotation  AGAACCGTAGACGTGGACGGTTGGCTCCACACCGGTGACATCGGATATGTGGACGACGACGACGATGTTTTCATTGTA

4CL3-hybrid-seq         GACAGGGTGAAGGAACTCATCAAATTCAAAGGCTTTCAGGTCCCACCAGCTGAACTGGAGGCTCTTCTCATCTCCCAT
```

4CL3-KF220556 GACAGGGTGAAGGAACTCATCAAATTCAAAGGCTTTCAGGTCCCACCAGCTGAACTGGAGGCTCTTCTCATCTCCCAT

4CL3-genome_annotation GACAGGGTGAAGGAACTCATCAAATTCAAAGGCTTTCAGGTCCCACCAGCTGAACTGGAGGCTCTTCTCATCTCCCAT

4CL3-hybrid-seq CCCCAAATTTCTGATGCTGCCGTTGTACCGCAAAAAGATGAAGCTGCTGGTGAAGTTCCCGTGGCTTTTGTAGTTCCA

4CL3-KF220556 CCCCAAATTTCTGATGCTGCCGTTGTACCGCAAAAAGATGAAGCTGCTGGTGAAGTTCCCGTGGCTTTTGTAGTTCCA

4CL3-genome_annotation CCCCAAATTTCTGATGCTGCCGTTGTACCGCAAAAAGATGAAGCTGCTGGTGAAGTTCCCGTGGCTTTTGTAGTTCCA

4CL3-hybrid-seq TCAAATGGATCCGAACTAACTGAAGAAGCTGTCAAAGAATTCATTTCCAAACAGGTTGTGTTTTATAAGAGACTGCAC

4CL3-KF220556 TCAAATGGATCCGAACTAACTGAAGAAGCTGTCAAAGAATTCATTTCCAAACAGGTTGTGTTTTATAAGAGACTGCAC

4CL3-genome_annotation TCAAATGGATCCGAACTAACTGAAGAAGCTGTCAAAGAATTCATTTCCAAACAGGTTGTGTTTTATAAGAGACTGCAC

4CL3-hybrid-seq AAAGTGTACTTTGTTCACGCCATTCCCAAGTCGCCAGCTGGTAAAATATTGAGGAAAGATCTGAGAGCCAAACTTGCT

4CL3-KF220556 AAAGTGTACTTTGTTCACGCCATTCCCAAGTCGCCAGCTGGTAAAATATTGAGGAAAGATCTGAGAGCCAAACTTGCT

4CL3-genome_annotation AAAGTGTACTTTGTTCACGCCATTCCCAAGTCGCCAGCTGGTAAAATATTGAGGAAAGATCTGAGAGCCAAACTTGCT

4CL3-hybrid-seq TCTGCAGCTTCCCCCACATCTTAATTTTATATTCAACCTTCGGAAGTATTTTATTTAGATCTATATATCATTTCTTGC

4CL3-KF220556 TCTGCAGCTTCCCCCACATCTTAA--

4CL3-genome_annotation TCTGCAGCTTCCCCCACATCTTAA--

4CL3-hybrid-seq CTCGCCTCTATTGCCAAATTTCCTTGTTTATATAATTTATTT

4CL3-KF220556 --

4CL3-genome_annotation --

#Sm4CL-like2

4CL-like2-hybrid-seq AAAATGTGATTAAATGCGAGGCCAGCCACAACATTGAGTTCTGGAAATTCGCAGTAATCATTGATACTCCACTACAAA

4CL-like2-KF220558 --

4CL-like2-genome_annotation --

4CL-like2-hybrid-seq GAAGATGGAGAAATCCGGTTATGGCCGCGACGGCGTGTTCCGATCTCTGCGCCCGCCGTTATCCCTTCCCACAAACCC

4CL-like2-KF220558 ----ATGGAGAAATCCGGTTATGGCCGCGACGGCGTGTTCCGATCTCTGCGCCCGCCGTTATCCCTTCCCACAAACCC

4CL-like2-genome_annotation ----ATGGAGAAATCCGGTTATGGCCGCGACGGCGTGTTCCGATCTCTGCGCCCGCCGTTATCCCTTCCCACAAACCC

4CL-like2-hybrid-seq CAACCTCTCCTTGGTCTCCTTCCTCTTCAGAAACACTTCGTCCTTCGCCGAAAAACGCGCCCTAATCGACGCGCACAC

4CL-like2-KF220558 CAACCTCTCCTTGGTCTCCTTCCTCTTCAGAAACACTTCGTCCTTCGCCGAAAAACGCGCCCTAATCGACGCGCACAC

4CL-like2-genome_annotation CAACCTCTCCTTGGTCTCCTTCCTCTTCAGAAACACTTCGTCCTTCGCCGAAAAACGCGCCCTAATCGACGCGCACAC

4CL-like2-hybrid-seq CGGCGAAACCCTCACTTTCTCTCACTTCAAATCCATGGTTTCCAGAGTCGCCCACGGCTTGCTCCAGCTCGGCATCAA

4CL-like2-KF220558 CGGCGAAACCCTCACTTTCTCTCACTTCAAATCCATGGTTTCCAGAGTCGCCCACGGCTTGCTCCAGCTCGGCATCAA

4CL-like2-genome_annotation CGGCGAAACCCTCACTTTCTCTCACTTCAAATCCATGGTTTCCAGAGTCGCCCACGGCTTGCTCCAGCTCGGCATCAA

4CL-like2-hybrid-seq GAAAAACGACGTTGTCCTCATTTTCTCCCCCAATTCGATCCAGTTCCCGCTCTGTTTCTTCGGGATTATTGCCATCGG

4CL-like2-KF220558 GAAAAACGACGTTGTCCTCATTTTCTCCCCCAATTCGATCCAGTTCCCGCTCTGTTTCTTCGGGATTATTGCCATCGG

4CL-like2-genome_annotation GAAAAACGACGTTGTCCTCATTTTCTCCCCCAATTCGATCCAGTTCCCGCTCTGTTTCTTCGGGATTATTGCCATCGG

```
4CL-like2-hybrid-seq         CGCCGTCGCCACCACCGTCAACCCCGTCTACACCGCCAGGGAGGTCTCGAAGCAGCTCAAGGATTCCATGGCCAAGAT
4CL-like2-KF220558           CGCCGTCGCCACCACCGTCAACCCCGTCTACACCGCCAGGGAGGTCTCGAAGCAGCTCAAGGATTCCATGGCCAAGAT
4CL-like2-genome_annotation  CGCCGTCGCCACCACCGTCAACCCCGTCTACACCGCCAGGGAGGTCTCGAAGCAGCTCAAGGATTCCATGGCCAAGAT

4CL-like2-hybrid-seq         GATCATCACCGTCGAGGAGTTGCTGCCCAAGGTCAACGATTTTGACCTCCCCATTATTCTGCTCGGCGATTCGGAGAA
4CL-like2-KF220558           GATCATCACCGTCGAGGAGTTGCTGCCCAAGGTCAACGATTTTGACCTCCCCATTATTCTGCTCGGCGATTCGGAGAA
4CL-like2-genome_annotation  GATCATCACCGTCGAGGAGTTGCTGCCCAAGGTCAACGATTTTGACCTCCCCATTATTCTGCTCGGCGATTCGGAGAA

4CL-like2-hybrid-seq         ACCCGTTTCGCCGAATGGGAGAACCCCTAAAATCACTCTGTTTTCGAAGCTCGTCGAAAATGAGGGCTCCGTCGATAT
4CL-like2-KF220558           ACCCGTTTCGCCGAATGGGAGAACCCCTAAAATCACTCTGTTTTCGAAGCTCGTCGAAAATGAGGGCTCCGTCGATAT
4CL-like2-genome_annotation  ACCCGTTTCGCCGAATGGGAGAACCCCTAAAATCACTCTGTTTTCGAAGCTCGTCGAAAATGAGGGCTCCGTCGATAT

4CL-like2-hybrid-seq         AGACGGCAATTCATTCGGAATCAAACAAAACGACACAGCTGCGCTGCTATACTCTTCGGGGACAACTGGCGCGAGCAA
4CL-like2-KF220558           AGACGGCAATTCATTCGGAATCAAACAAAACGACACAGCTGCGCTGCTATACTCTTCGGGGACAACTGGCGCGAGCAA
4CL-like2-genome_annotation  AGACGGCAATTCATTCGGAATCAAACAAAACGACACAGCTGCGCTGCTATACTCTTCGGGGACAACTGGCGCGAGCAA

4CL-like2-hybrid-seq         GGGCGTGATTCTCACTCATCGAAACTTCATCGCCTCCTCGCAAATGATCACCGCCGATCAGGAGATGGCGGGGGAGGT
4CL-like2-KF220558           GGGCGTGATTCTCACTCATCGAAACTTCATCGCCTCCTCGCAAATGATCACCGCCGATCAGGAGATGGCGGGGGAGGT
4CL-like2-genome_annotation  GGGCGTGATTCTCACTCATCGAAACTTCATCGCCTCCTCGCAAATGATCACCGCCGATCAGGAGATGGCGGGGGAGGT

4CL-like2-hybrid-seq         CAACTGCGTGTTCATGTGCGTGCTGCCGATGTTCCACGTGTTCGGCCTCGCGGTGATCATGCTCTCGCAGCTGCAGCG
4CL-like2-KF220558           CAACTGCGTGTTCATGTGCGTGCTGCCGATGTTCCACGTGTTCGGCCTCGCGGTGATCATGCTCTCGCAGCTGCAGCG
4CL-like2-genome_annotation  CAACTGCGTGTTCATGTGCGTGCTGCCGATGTTCCACGTGTTCGGCCTCGCGGTGATCATGCTCTCGCAGCTGCAGCG

4CL-like2-hybrid-seq         CGGCAGCGCCATCATCTCGATGTCCAAGTTCGATTTCGAGATGCTTTTGAGGACGGTGGAGAAGTATGCGGTGACGCA
4CL-like2-KF220558           CGGCAGCGCCATCATCTCGATGTCCAAGTTCGATTTCGAGATGCTTTTGAGGACGGTGGAGAAGTATGCGGTGACGCA
4CL-like2-genome_annotation  CGGCAGCGCCATCATCTCGATGTCCAAGTTCGATTTCGAGATGCTTTTGAGGACGGTGGAGAAGTATGCGGTGACGCA

4CL-like2-hybrid-seq         TTTGTGGGTGGTGCCGCCGATCATACTGGCGTTGGCCAAGAACAGTGTTGTGAAGAAGTACGATATGTCGTCGTTGAA
4CL-like2-KF220558           TTTGTGGGTGGTGCCGCCGATCATACTGGCGTTGGCCAAGAACAGTGTTGTGAAGAAGTACGATATGTCGTCGTTGAA
4CL-like2-genome_annotation  TTTGTGGGTGGTGCCGCCGATCATACTGGCGTTGGCCAAGAACAGTGTTGTGAAGAAGTACGATATGTCGTCGTTGAA

4CL-like2-hybrid-seq         GCAGGTTGGATCGGGGGCTGCTCCACTCGGGAAGGAGTTGATGCAGGAATGTGCCAAGACTTTCCCTCACGCTGTTGT
4CL-like2-KF220558           GCAGGTTGGATCGGGGGCTGCTCCACTCGGGAAGGAGTTGATGCAGGAATGTGCCAAGACTTTCCCTCAGGCTGTTGT
4CL-like2-genome_annotation  GCAGGTTGGATCGGGGGCTGCTCCACTCGGGAAGGAGTTGATGCAGGAATGTGCCAAGACTTTCCCTCAGGCTGTTGT

4CL-like2-hybrid-seq         TATGCAAGGGTATGGAATGACAGAAACCTGTGGAATTGTTTCTGTCGAGAATGCATATGCAGGGCCTCGACATTCTGG
4CL-like2-KF220558           TATGCAAGGGTATGGAATGACAGAAACCTGTGGAATTGTTTCTGTCGAGAATGCATATGCAGGGCCTCGACATTCTGG
4CL-like2-genome_annotation  TATGCAAGGGTATGGAATGACAGAAACCTGTGGAATTGTTTCTGTCGAGAATGCATATGCAGGGCCTCGACATTCTGG

4CL-like2-hybrid-seq         TTCCGCTGGCATGCTTGTTCCGGGAGTGGAGTGTCAAATAGTTAGTGTCGAAACATTGAAGCCTCTTCCGCCAACCCA
4CL-like2-KF220558           TTCCGCTGGCATGCTTGTTCCGGGAGTGGAGTGTCAAATAGTTAGTGTCGAAACATTGAAGCCTCTTCCGCCAACCCA
4CL-like2-genome_annotation  TTCCGCTGGCATGCTTGTTCCGGGAGTGGAGTGTCAAATAGTTAGTGTCGAAACATTGAAGCCTCTTCCGCCAACCCA
```

4CL-like2-hybrid-seq GATGGGGGAAATTTGGGTTCGAGGACAAAATATGATGCAAGGTTATTTCAGGAATCCGCAAGCTACAAAGCTTACGAT

4CL-like2-KF220558 GATGGGGGAAATTTGGGTTCGAGGACAAAATATGATGCAAGGTTATTTCAGGAATCCGCAAGCTACAAAGCTTACGAT

4CL-like2-genome_annotation GATGGGGGAAATTTGGGTTCGAGGACAAAATATGATGCAAGGTTATTTCAGGAATCCGCAAGCTACAAAGCTTACGAT

4CL-like2-hybrid-seq AGATAAACAGGGTTGGGTGCATACGGGAGATCTTGGATACTTTGATGAGGATGGCCTGCTATATGTTGTTGACCGAAT

4CL-like2-KF220558 AGATAAACAGGGTTGGGTGCATACGGGAGATCTTGGATACTTTGATGAGGATGGCCTGCTATATGTTGTTGACCGAAT

4CL-like2-genome_annotation AGATAAACAGGGTTGGGTGCATACGGGAGATCTTGGATACTTTGATGAGGATGGCCTGCTATATGTTGTTGACCGAAT

4CL-like2-hybrid-seq CAAAGAGCTGATCAAGTATAAGGGCTTGCAGGTTGCTCCAGCAGAGCTCGAAGGGCTGCTCGTCTCCCATCCCGAGAT

4CL-like2-KF220558 CAAAGAGCTGATCAAGTATAAGGGCTTGCAGGTTGCTCCAGCAGAGCTCGAAGGGCTGCTCGTCTCGCATCCCGAGAT

4CL-like2-genome_annotation CAAAGAGCTGATCAAGTATAAGGGCTTGCAGGTTGCTCCAGCAGAGCTCGAAGGGCTGCTCGTCTCGCATCCCGAGAT

4CL-like2-hybrid-seq ATCAGATGCTGTGGTCATCCCATATCCGGACGCAGAAGCTGGTGAAGTTCCGGCTGCTTATGTCGTTCGGGCACCCAA

4CL-like2-KF220558 ATCAGATGCTGTGGTCATCCCATATCCGGATGCAGAAGCTGGTGAAGTTCCGGCTGCTTATGTCGTTCGGGCACCCAA

4CL-like2-genome_annotation ATCAGATGCTGTGGTCATCCCATATCCGGATGCAGAAGCTGGTGAAGTTCCGGCTGCTTATGTCGTTCGGGCACCCAA

4CL-like2-hybrid-seq CAGCTCACTGTCCGAAGAAGATATCAAGAAATTCATCGCCGATCAGGTTGCGCCTTTCAAAAGATTGAGGAGAGTTAC

4CL-like2-KF220558 CAGCTCACTGTCCGAAGAAGATATCAAGAAATTCATCGCCGATCAGGTTGCGCCTTTCAAAAGATTGAGGAGAGTTAC

4CL-like2-genome_annotation CAGCTCACTGTCCGAAGAAGATATCAAGAAATTCATCGCCGATCAGGTTGCGCCTTTCAAAAGATTGAGGAGAGTTAC

4CL-like2-hybrid-seq ATTCATAAACAGTGTCCCAAAATCGGCTTCAGGCAAAATACTGAGGCGAGAACTGATCGACAAAGTCCGATCAAACTT

4CL-like2-KF220558 ATTCATAAACAGTGTCCCAAAATCGGCTTCAGGCAAAATACTGAGGCGAGAACTGATCGACAAAGTCCGATCAAACTT

4CL-like2-genome_annotation ATTCATAAACAGTGTCCCAAAATCGGCTTCAGGCAAAATACTGAGGCGAGAACTGATCGACAAAGTCCGATCAAACTT

4CL-like2-hybrid-seq GTAATAATCTATTTTATCTCCACCTTGCCTATGTATGTTGTCAGAAATAAATTCTTCCTAAACTTTTGGGGGTTATGT

4CL-like2-KF220558 GTAA--

4CL-like2-genome_annotation GTAA--

4CL-like2-hybrid-seq CATGTCATGCCTCATTTGATGATTCACATGGATGTATAAAAATAGAGAATTGATAAGGAAGTAGTTTATTTATTTATG

4CL-like2-KF220558 --

4CL-like2-genome_annotation --

4CL-like2-hybrid-seq TATTCGACAGAAAATTCGCT

4CL-like2-KF220558 --------------------

4CL-like2-genome_annotation --------------------

#Sm4CL-like4

4CL-like4-hybrid-seq GGGCAACCAAATTCATTTTCATTTACATATCTCATCCTTATTTCCCATTCATTCATTTTATTTATTTTTCTAGAGATT

4CL-like4-KF220560 --

4CL-like4-genome_annotation --

4CL-like4-hybrid-seq TCCAATGGCTATCTCCACTCTCACCGCCTTGCTGAACCATGTCGCCGAGAAATTCCCTTCCCGCCGCGCCATCAGCGT

4CL-like4-KF220560 ----ATGGCTATCTCCACTCTCACCGCCTTGCTGAACCATGTCGCCGAGAAATTCCCTTCCCGCCGCGCCATCAGCGT

4CL-like4-genome_annotation ----ATGGCTATCTCCACTCTCACCGCCTTGCTGAACCATGTCGCCGAGAAATTCCCTTCCCGCCGCGCCATCAGCGT

```
4CL-like4-hybrid-seq          CTCCGGCAAGTTCGATCTCACCCATGCACGCCTCAATCAGCTCGTTGATCACGCCGCCGCTCAGCTCGTCGCCGCCGG
4CL-like4-KF220560            CTCCGGCAAGTTCGATCTCACCCATGCACGCCTCAATCAGCTCGTTGATCACGCCGCCGCTCAGCTCGTCGCCGCCGG
4CL-like4-genome_annotation   CTCCGGCAAGTTCGATCTCACCCATGCACGCCTCAATCAGCTCGTTGATCACGCCGCCGCTCAGCTCGTCGCCGCCGG

4CL-like4-hybrid-seq          CGTCAAGCCCGGCGACGTCGTCGCGCTCACCTTCCCTAACGCCGTTGAGTTTGTTATAATGTTTCTGGCCGTGATACG
4CL-like4-KF220560            CGTCAAGCCCGGCGACGTCGTCGCGCTCACCTTCCCTAACGCCGTTGAGTTTGTTATAATGTTTCTGGCCGTGATACG
4CL-like4-genome_annotation   CGTCAAGCCCGGCGACGTCGTCGCGCTCACCTTCCCTAACGCCGTTGAGTTTGTTATAATGTTTCTGGCCGTGATACG

4CL-like4-hybrid-seq          AGCCCGCGCAACGGCGGCGCCGCTTAACGCCACCTACACATTTGATGAATTCGATTTCTACTTATCGGATTCAGAATC
4CL-like4-KF220560            AGCCCGCGCAACGGCGGCGCCGCTTAACGCCACCTACACATTTGATGAATTCGATTTCTACTTATCGGATTCAGAATC
4CL-like4-genome_annotation   AGCCCGCGCAACGGCGGCGCCGCTTAACGCCACCTACACATTTGATGAATTCGATTTCTACTTATCGGATTCAGAATC

4CL-like4-hybrid-seq          GAAGCTCCTACTCACATCAAAAGAAGGCAACGAGCAGGCACAGGCAGCGGCGGCGAAGCTCAATATTCCTCATCTCTC
4CL-like4-KF220560            GAAGCTCCTACTCACATCAAAAGAAGGCAACGAGCAGGCACAGGCAGCGGCGGCGAAGCTCAATATTCCTCATCTCTC
4CL-like4-genome_annotation   GAAGCTCCTACTCACATCAAAAGAAGGCAACGAGCAGGCACAGGCAGCGGCGGCGAAGCTCAATATTCCTCATCTCTC

4CL-like4-hybrid-seq          CGCCGCGCTGCCAGCCGGCGACTCGGAAATCGTCCTATCTCCGACTCAGTCGAACGTGGACACCGACTCGGTCGCTAA
4CL-like4-KF220560            CGCCGCGCTGCCAGCCGGCGACTCGGAAATCGTCCTATCTCCGACTCAGTCGAACGTGGACACCGACTCGGTCGCTAA
4CL-like4-genome_annotation   CGCCGCGCTGCCAGCCGGCGACTCGGAAATCGTCCTATCTCCGACTCAGTCGAACGTGGACACCGACTCGGTCGCTAA

4CL-like4-hybrid-seq          ACTCACTAACGAGCCTTCCGACGCTGCACTCTTCCTCCACACGTCCGGCACCACTAGTCGACCCAAAGGCGTGCCTCT
4CL-like4-KF220560            ACTCACTAACGAGCCTTCCGACGCTGCACTCTTCCTCCACACGTCCGGCACCACTAGTCGACCCAAAGGCGTGCCTCT
4CL-like4-genome_annotation   ACTCACTAACGAGCCTTCCGACGCTGCACTCTTCCTCCACACGTCCGGCACCACTAGTCGACCCAAAGGCGTGCCTCT

4CL-like4-hybrid-seq          AACTCAGCAGAATCTCTACTCCTCTGTCCAAAATATCAAATCAGTCTACAAATTAACCGAATCGGACTCCACCGTCAT
4CL-like4-KF220560            AACTCAGCAGAATCTCTACTCCTCTGTCCAAAATATCAAATCAGTCTACAAATTAACCGAATCGGACTCCACCGTCAT
4CL-like4-genome_annotation   AACTCAGCAGAATCTCTACTCCTCTGTCCAAAATATCAAATCAGTCTACAAATTAACCGAATCGGACTCCACCGTCAT

4CL-like4-hybrid-seq          CGTCCTGCCCCTGTTCCACGTCCACGGCCTCTTAGCCGGCTTACTGAGCTCGCTCGGCGCCGGCGCCGCGGTAGCACT
4CL-like4-KF220560            CGTCCTGCCCCTGTTCCACGTCCACGGCCTCTTAGCCGGCTTACTGAGCTCGCTCGGCGCCGGCGCCGCGGTAGCACT
4CL-like4-genome_annotation   CGTCCTGCCCCTGTTCCACGTCCACGGCCTCTTAGCCGGCTTACTGAGCTCGCTCGGCGCCGGCGCCGCGGTAGCACT

4CL-like4-hybrid-seq          TCCCGCCGCCGGGAGATTCTCGGCGTCGACCTTCTGGTCCGACATGAAGAGATACGACGCGACGTGGTACACCGCCGT
4CL-like4-KF220560            TCCCGCCGCCGGGAGATTCTCGGCGTCGACCTTCTGGTCCGACATGAAGAGATACGACGCGACGTGGTACACCGCCGT
4CL-like4-genome_annotation   TCCCGCCGCCGGGAGATTCTCGGCGTCGACCTTCTGGTCCGACATGAAGAGATACGACGCGACGTGGTACACCGCCGT

4CL-like4-hybrid-seq          GCCTACAATACACCAGATCGTGCTCGACCGCCACCTCAGCAAGCCCGAGCCCGCTTATCCCAAGCTCCGCTTCATCCG
4CL-like4-KF220560            GCCTACAATACACCAGATCGTGCTCGACCGCCACCTCAGCAAGCCCGAGCCCGCTTATCCCAAGCTCCGCTTCATCCG
4CL-like4-genome_annotation   GCCTACAATACACCAGATCGTGCTCGACCGCCACCTCAGCAAGCCCGAGCCCGCTTATCCCAAGCTCCGCTTCATCCG

4CL-like4-hybrid-seq          TAGCTGCAGCGCGTCGCTGGCTCCTTCTATACTGGCTCGGCTGGAAGAGGCGTTCGGCGCGCCGGTGCTGGAGGCGTA
4CL-like4-KF220560            TAGCTGCAGCGCGTCGCTGGCTCCTTCTATACTGGCTCGGCTGGAAGAGGCGTTCGGCGCGCCGGTGCTGGAGGCGTA
4CL-like4-genome_annotation   TAGCTGCAGCGCGTCGCTGGCTCCTTCTATACTGGCTCGGCTGGAAGAGGCGTTCGGCGCGCCGGTGCTGGAGGCGTA
```

```
4CL-like4-hybrid-seq           CGCGATGACGGAAGCGACGCATCTGATGACGTCGAATCCGTTGCCGGAGGAGGGGCCGCACAAGGCCGGGTCGGTGGG
4CL-like4-KF220560             CGCGATGACGGAAGCGACGCATCTGATGACGTCGAATCCGTTGCCGGAGGAGGGGCCGCACAAGGCCGGGTCGGTGGG
4CL-like4-genome_annotation    CGCGATGACGGAAGCGACGCATCTGATGACGTCGAATCCGTTGCCGGAGGAGGGGCCGCACAAGGCCGGGTCGGTGGG

4CL-like4-hybrid-seq           GAAGCCTGTGGGGCAGGAGATGGCGATTTTGGACCAGAATGGTGTTGTGCAAGAGCCTAATGCTACTGGAGAGGTTTG
4CL-like4-KF220560             GAAGCCTGTGGGGCAGGAGATGGCGATTTTGGACCAGAATGGTGTTGTGCAAGAGCCTAATGCTACTGGAGAGGTTTG
4CL-like4-genome_annotation    GAAGCCTGTGGGGCAGGAGATGGCGATTTTGGACCAGAATGGTGTTGTGCAAGAGCCTAATGCTACTGGAGAGGTTTG

4CL-like4-hybrid-seq           CATAAGGGGACCTAATGTGACCAAAGGTTACAAGAATAATGTTGACGCCAATAAATCGGCTTTTCTGTTTGGATGGTT
4CL-like4-KF220560             CATAAGGGGACCTAATGTGACCAAAGGTTACAAGAATAATGTTGACGCCAATAAATCGGCTTTTCTGTTTGGATGGTT
4CL-like4-genome_annotation    CATAAGGGGACCTAATGTGACCAAAGGTTACAAGAATAATGTTGACGCCAATAAATCGGCTTTTCTGTTTGGATGGTT

4CL-like4-hybrid-seq           CCATACTGGAGATTTGGGATACTTGGATTCAGATGGATATCTGCATCTTGTTGGCAGAATCAAGGAAATGATCAATCG
4CL-like4-KF220560             CCATACTGGAGATTTGGGATACTTGGATTCAGATGGATATCTGCATCTTGTTGGCAGAATCAAGGAAATGATCAATCG
4CL-like4-genome_annotation    CCATACTGGAGATTTGGGATACTTGGATTCAGATGGATATCTGCATCTTGTTGGCAGAATCAAGGAAATGATCAATCG

4CL-like4-hybrid-seq           CGGAGGAGAAAACATTTCACCAATTGAAGTAGATGCAGTCCTATTGTCCCATCCTGATGTTGCACAAGGTGTTGCATT
4CL-like4-KF220560             CGGAGGAGAAAAGATTTCACCAATTGAAGTAGATGCAGTCCTATTGTCCCATCCTGATGTTGCACAAGGTGTTGCATT
4CL-like4-genome_annotation    CGGAGGAGAAAAGATTTCACCAATTGAAGTAGATGCAGTCCTATTGTCCCATCCTGATGTTGCACAAGGTGTTGCATT

4CL-like4-hybrid-seq           TGGTGTCCCAGATGACAAATATGGTGAAGAGATTAACTGTGCAGTTATTCCAAGAGACGAATCAAATCTTGATGAAGA
4CL-like4-KF220560             TGGTGTCCCAGATGACAAATATGGTGAAGAGATTAACTGTGCAGTTATTCCAAGAGACGAATCAAATCTTGATGAAGA
4CL-like4-genome_annotation    TGGTGTCCCAGATGACAAATATGGTGAAGAGATTAACTGTGCAGTTATTCCAAGAGACGAATCAAATCTTGATGAAGA

4CL-like4-hybrid-seq           AGAGGTTATCAAGTTCTGCAAGAAAAATTTGGCAGCATTCAAGGTTCCAAAGAAGGTTTTCATCACCGATTCACTCCC
4CL-like4-KF220560             AGAGGTTATCAAGTTCTGCAAGAAAAATTTGGCAGCATTCAAGGTTCCAAAGAAGGTTTTCATCACCGATTCACTCCC
4CL-like4-genome_annotation    AGAGGTTATCAAGTTCTGCAAGAAAAATTTGGCAGCATTCAAGGTTCCAAAGAAGGTTTTCATCACCGATTCACTCCC

4CL-like4-hybrid-seq           CAAAACCGCATCCGGCAAAATCCAGAGGCGGATTGTTTCCGAGCACTTCCTTGCACAAATATCTACCGCCAAGGTGCC
4CL-like4-KF220560             CAAAACCGCATCCGGCAAAATCCAGAGGCGGATTGTTTCCGAGCACTTCCTTGCACAAATATCTACCGCCAAGGTGCC
4CL-like4-genome_annotation    CAAAACCGCATCCGGCAAAATCCAGAGGCGGATTGTTTCCGAGCACTTCCTTGCACAAATATCTACCGCCAAGGTGCC

4CL-like4-hybrid-seq           CAAGTTTGGAGCCTAAGCTGCTGCTAGTTTCATATGGCTCGTTGGAAATCATGATGACACCAAGATTTGAAAGCAATA
4CL-like4-KF220560             CAAGTTTGGAGCCTAA~---------------------------------------------------------------
4CL-like4-genome_annotation    CAAGTTTGGAGCCTAA----------------------------------------------------------------

4CL-like4-hybrid-seq           AATAATATTATATGCCAGTGTTGCAGTCGTCAATATTCATGATTCAAGTTTCCATGATATGCAGATTTTTGTCACTGT
4CL-like4-KF220560             --------------------------------------------------------------------------------
4CL-like4-genome_annotation    --------------------------------------------------------------------------------

4CL-like4-hybrid-seq           TGATGTTAATCATAGGGCCATAACATAGTTATGTACTCCTTTAGTTTGCTCAATTAAG
4CL-like4-KF220560             ----------------------------------------------------------
4CL-like4-genome_annotation    ----------------------------------------------------------
```

#Sm4CL-like5

```
4CL-like5-hybrid-seq         AGGACAGCAATTAACTCTCATTTCCATGGCGGAAAATCAATCACTCCACTGGCACCGCAGTGACCCCCAAAGCGGCTA
4CL-like5-KF220561           --------------------------------------------------------------------------------
4CL-like5-genome_annotation  -------------------------ATGGCGGAAAATCAATCACTCCACTGGCACTGCAGTGACCCCCAAAGCGGCTA

4CL-like5-hybrid-seq         TTGCTCACAAACCAGAATTTACCGCAGCCTCCACCCCGCCATCCCTCTTCCTCCGCCATCCGCGCCCCTCTCCATCTC
4CL-like5-KF220561           --------------------------------------------------------------------------------
4CL-like5-genome_annotation  TTGCTCACAAACCAGAATTTACCGCAGCCTCCACCCCGCCATCCCTCTTCCTCCGCCATCCGCGCCCCTCTCCATCTC

4CL-like5-hybrid-seq         CTCCTACGCCATATCCCTTCTTCACTCCCCCACCAACCCTACCTCCGCCGCCCTCTCCAACACCGCATTCCTCATCGA
4CL-like5-KF220561           --------------------------------------------------------------------------------
4CL-like5-genome_annotation  CTCCTACGCCATATCCCTTCTTCACTCCCCCACCACCCCTACCTCCGCCGCCCTCTCCAACACCGCATTCCTCATCGA

4CL-like5-hybrid-seq         TGCAGACACCGGCCACCGCCTCACTTACTCCGCTTTCCTCCAGCGAGTCAGGTCCCTCTCATCCTCCCTCAACTCCCT
4CL-like5-KF220561           --------------------------------------------------------------------------------
4CL-like5-genome_annotation  TGCAGACACCGGCCACCGCCTCACTTACTCCGCTTTCCTCCAGCGAGTCAGGTCCCTCTCATCCTCCCTCAACTCCCT

4CL-like5-hybrid-seq         CATCTCCAAGAACGACGTCGCTTTCATCATCTCCCCGCCCTCGTTCCACATCCCCGTCCTCTACTTCTCTCTCCTCTC
4CL-like5-KF220561           --------------------------------------------------------------------------------
4CL-like5-genome_annotation  CATCTCCAAGAACGACGTCGCTTTCATCATCTCCCCGCCCTCGTTCCACATCCCCGTCCTCTACTTCTCTCTCCTCTC

4CL-like5-hybrid-seq         TCTCGGCGTGGCCATCTCGCCGTCCAACCCGCTCTCCTCCCCGTCCGAGCTGGCCCATCAAATCCAGCTCAGCAAACC
4CL-like5-KF220561           --------------------------------------------------------------------------------
4CL-like5-genome_annotation  TCTCGGCGTGGCCATCTCGCCGTCCAACCCGCTCTCCTCCCCGTCCGAGCTGGCCCATCAAATCCAGCTCAGCAAACC

4CL-like5-hybrid-seq         CTCGGTCGCCTTCTCCACCTCAGCATTCGCCCACAAACTCCCGCCGAATCTCCGCGTCGTCCTCCTCGATTCCCCTGA
4CL-like5-KF220561           --------------------------------------------------------------------------------
4CL-like5-genome_annotation  CTCGGTCGCCTTCTCCACCTCAGCATTCGCCCACAAACTCCCGCCGAATCTCCGCGTCGTCCTCCTCGATTCCCCTGA

4CL-like5-hybrid-seq         GTTCCTCTCCATGCTTGAATCTAGGTCTCCGATCAGCTCCGCCGCCGCCGCCGTGAACCAGTCGGATACCGCCGCCGT
4CL-like5-KF220561           ----------ATGCTTGAATCTAGGTCTCCGATCAGCTCCGCCGCCGCCGCCGTGAACCAGTCGGATACCGCCGCCGT
4CL-like5-genome_annotation  GTTCCTCTCCATGCTTGAATCTAGGTCTCCGATCAGCTCCGCCGCCGCCGCCGTGAACCAGTCGGATACCGCCGCCGT

4CL-like5-hybrid-seq         ACTCTATTCCTCCGGCACCACCGGTAAAGTAAAAGGCGTCGTGTTGACCCACCGCAACTTGATAGCTGTGATAACGCA
4CL-like5-KF220561           ACTCTATTCCTCCGGCACCACCGGTAAAGTAAAAGGCGTCGTGTTGACCCACCGCAACTTGATAGCTGTGATAACGCA
4CL-like5-genome_annotation  ACTCTATTCCTCCGGCACCACCGGTAAAGTAAAAGGCGTCGTGTTGACCCACCGCAACTTGATAGCTGTGATAACGCA

4CL-like5-hybrid-seq         ATTTTATCACAACGCAAAAGCGAAACGCGGAGACATGGTCGTTTACGATCATCCTGTTTCGCTTTTCACGCTGCCGTT
4CL-like5-KF220561           ATTTTATCACAACGCAAAAGCGAAACGCGGAGACATGGTCGTTTACGATCATCCTGTTTCGCTTTTCACGCTGCCGTT
4CL-like5-genome_annotation  ATTTTATCACAACGCAAAAGCGAAACGCGGAGACATGGTCGTTTACGATCATCCTGTTTCGCTTTTCACGCTGCCGTT

4CL-like5-hybrid-seq         GTTCCACGTCTTTGGATTATTCATGATGATAAGGGCGGCGTCGATGGGGGAGAGCCTGGTGCTGATGGAGAAGTTCGA
4CL-like5-KF220561           GTTCCACGTCTTTGGATTATTCATGATGATAAGGGCGGCGTCGATGGGGGAGAGCCTGGTGCTGATGGAGAAGTTCGA
```

4CL-like5-genome_annotation GTTCCACGTCTTTGGATTATTCATGATGATAAGGGCGGCGTCGATGGGGGAGAGCCTGGTGCTGATGGAGAAGTTCGA

4CL-like5-hybrid-seq TTTTGTGAAGATGCTGGCGGCGGTGGAGAGATACAGAGTGACTTATATGCCGGTGGCGCCGCCGCTCATCATTGCGAT
4CL-like5-KF220561 TTTTGTGAAGATGCTGGCGGCGGTGGAGAGATACAGAGTGACTTATATGCCGGTGGCGCCGCCGCTCATCGTTGCGAT
4CL-like5-genome_annotation TTTTGTGAAGATGCTGGCGGCGGTGGAGAGATACAGAGTGACTTATATGCCGGTGGCGCCGCCGCTCATCATTGCGAT

4CL-like5-hybrid-seq GGTGAAGTCGGATTTGGTGGATAAGTATGACTTGAGCTCGCTGCAGGTGCTGGGATCGGGAGGAGCGCCGCTGGGGGA
4CL-like5-KF220561 GGTGAAGTCGGATTTGGTGGATAAGTATGACTTGAGCTCGCTGCAGGTGCTGGGATCGGGAGGAGCGCCGCTGGGGGA
4CL-like5-genome_annotation GGTGAAGTCGGATTTGGTGGATAAGTATGACTTGAGCTCGCTGCAGGTGCTGGGATCGGGAGGAGCGCCGCTGGGGGA

4CL-like5-hybrid-seq GGATGTTGCCGAGCGGTTCAAGGCTAAATTCCCTCACGTGGGTATTGTCCAGGGCTATGGTTTGACTGAGACTGCAGG
4CL-like5-KF220561 GGATGTTGCCGAGCGGTTCAAGGCTAAATTCCCTCACGTGGGTATTGTCCAGGGCTATGGTTTGACTGAGACTGCAGG
4CL-like5-genome_annotation GGATGTTGCCGAGCGGTTCAAGGCTAAATTCCCTCACGTGGGTATTGTCCAGGGCTATGGTTTGACTGAGACTGCAGG

4CL-like5-hybrid-seq AGGGATCACACGTACAACTGAAGCTGAGGA-GTCCAGCGATATGGATCTGTAGGCCGCTTGGCTGAGAACATGGAAGC
4CL-like5-KF220561 AGGGATCACACGTATAACTGAAGCTGAGGATGTCCAGCGATATGGATCTGTAGGCCGCTTGGCTGAGAACATGGAAGC
4CL-like5-genome_annotation AGGGATCACACGTACAACTGAAGCTGAGGAGGTCCAGCGATATGGATCTGTAGGCCGCTTGGCTGAGAACATGGAAGC

4CL-like5-hybrid-seq CAAGATAGTTGATCCTGAGAGTGGGGAGTCCTTACCTCCTGGACAACGTGGAGAGTTATGGTTACGAGGGCCTGCAGT
4CL-like5-KF220561 CAAGATAGTTGATCCTGAGAGTGGGGAGTCCTTACCTCCTGGACAACGTGGAGAGTTATGGTTACGAGGGCCTGCAGT
4CL-like5-genome_annotation CAAGATAGTTGATCCTGAGAGTGGGGAGTCCTTACCTCCTGGACAACGTGGAGAGTTATGGTTACGAGGGCCTGCAGT

4CL-like5-hybrid-seq TATGAAAGGTTATGCAGGAGACGATGCAGCTACAGCTGCAACCTTGGATTCAGAAGGGTGGTTAAAGACCGGGGATCT
4CL-like5-KF220561 TATGAAAGGTTATGCAGGAGACGATGCAGCTACAGCTGCAACCTTGGATTCAGAAGGGTGGTTAAAGACCGGGGATCT
4CL-like5-genome_annotation TATGAAAGGTTATGCAGGAGACGATGCAGCTACAGCTGCAACCTTGGATTCAGAAGGGTGGTTAAAGACCGGGGATCT

4CL-like5-hybrid-seq ATGTTATTTTGACTCTGACGGGTTACTCTACATTGTTGATAGGTTGAAAGAATTGATAAAATACAAGGGATATCAGGT
4CL-like5-KF220561 ATGTTATTTTGACTCTGACGGGTTACTCTACATTGTTGATAGGTTGAAAGAATTGATAAAATACAAGGGATATCAGGT
4CL-like5-genome_annotation ATGTTATTTTGACTCTGACGGGTTACTCTACATTGTTGATAGGTTGAAAGAATTGATAAAATACAAGGGATATCAGGT

4CL-like5-hybrid-seq TCCGCCAGCAGAGCTGGAACAATTACTTCAGTCAATTCCTCAAATTGCTGATGCAGCAGTGATCCCATATCCTGATGA
4CL-like5-KF220561 TCCGCCAGCAGAGCTGGAACAATTACTTCAGTCAATTCCTCAAATTGCTGATGCAGCAGTGATCCCATATCCTGATGA
4CL-like5-genome_annotation TCCGCCAGCAGAGCTGGAACAATTACTTCAGTCAATTCCTCAAATTGCTGATGCAGCAGTGATCCCATATCCTGATGA

4CL-like5-hybrid-seq AGAAGCAGGCCAGATTCCCATGGCCTGTGTTGTGAGAAAACCCGGTACCACCATTTCTGCAACTCAGATAATGGAGTT
4CL-like5-KF220561 AGAAGCAGGCCAGATTCCCATGGCCTGTGTTGTGAGAAAACCCGGTACCACCATTTCTGCAACTCAGATAATGGAGTT
4CL-like5-genome_annotation AGAAGCAGGCCAGATTCCCATGGCCTGTGTTGTGAGAAAGCCCGGTACCACCATTTCTGCAACTCAGATAATGGAGTT

4CL-like5-hybrid-seq GATCGCGAAACAG---------------------------GTTGCTCCATATAAAAAGATTAGACGAGTTTCATTTGT
4CL-like5-KF220561 GATCGCGAAACAG---------------------------GTTGCTCCATATAAAAAGATTAGACGAGTTTCATTTGT
4CL-like5-genome_annotation GATCGCGAAACAGGCAAGTCAGCTTACTTCCATAAGCTGTGTTGCTCCATATAAAAAGATTAGACGAGTTTCATTTGT

4CL-like5-hybrid-seq GAATGCAATCCCAAAATCTCCAGCAGGAAAGATCTTACGGCGGGAGTTGGTTCATCTTGCTCTCTCCACTTCTTCATC
4CL-like5-KF220561 GAATGCAATCCCAAAATCTCCAGCAGGAAAGATCTTGCGGCGGGAGTTGGTTCATCTTGCTCTCTCCACTTCTTCATC

```
4CL-like5-genome_annotation GAATGCAATCCCAAAATCTCCAGCAGGAAAGATCTTACGGCGGGAGTTGGTTCATCTTGCTCTCTCCACTTCTTCATC

4CL-like5-hybrid-seq        TAAATTGTAAAAATCAAATACTATATCACATACCTCACAATTTCCAAAGTCTTGTTTCCAATTGTTCTTTTCTCCCTT
4CL-like5-KF220561          TAAATTGTAA--------------------------------------------------------------------
4CL-like5-genome_annotation TAAATTGTAA--------------------------------------------------------------------

4CL-like5-hybrid-seq        GTTGTAAAAGACCAATAATTAAAGGCCTGGGTGAAAATACAATACTACTTTTTGGTC
4CL-like5-KF220561          ---------------------------------------------------------
4CL-like5-genome_annotation ---------------------------------------------------------

#Sm4CL-like7
4CL-like7-hybrid-seq        GCCCCGTTGTCATCAGCTGAAAGCTCATTCCCATGGCTGAAACCAGCTCCTTCAATCCCCAAAATGGCTTCTGCCCAC
4CL-like7-KF220563          ------------------------------------------------------------------------------
4CL-like7-genome_annotation --------------------------------ATGGCTGAAACCAGCTCCTTCAATCCCCAAAATGGCTTCTGCCCAC

4CL-like7-hybrid-seq        AAACCAAAATATACCACAGCCTCCGCCCCGCCGTTCCTCTCCCGCCGCCGTCTCAACACCTCTCCATCTTCCAATACG
4CL-like7-KF220563          ------------------------------------------------------------------------------
4CL-like7-genome_annotation AAACCAAAATATACCACAGCCTCCGCCCCGCCGTTCCTCTCCCGCCGCCGTCTCAACACCTCTCCATCTTCCAATACG

4CL-like7-hybrid-seq        CTCTCTCCCTCCTCCACTCAACCGCCAACCCCAGCACCCCCTTCCTCATCGATGCCGCCACCTCCCGCCACCTCACCT
4CL-like7-KF220563          ------------------------------------------------------------------------------
4CL-like7-genome_annotation CTCTCTCCCTCCTCCACTCAACCGCCAACCCCAGCACCCCCTTCCTCATCGATGCCGCCACCTCCCGCCACCTCACCT

4CL-like7-hybrid-seq        ACTCCGCCTTCCTCCGCCAAGTGGCCTCCCTCTCCGCCTCCCTCAAACCCCTCCTCTCCAACAACGACGTCGCCTTCA
4CL-like7-KF220563          ------------------------------------------------------------------------------
4CL-like7-genome_annotation ACTCCGCCTTCCTCCGCCAAGTGGCCTCCCTCTCCGCCTCCCTCAAACCCCTCCTCTCCAACAACGACGTCGCCTTCA

4CL-like7-hybrid-seq        TCCTCTCCCCGGCATCCCTCCACATCCCCGTCCTCTACTTCTCTCTCCTCTCTCTAGGCGTCGCCGTCTCCCCCTCCA
4CL-like7-KF220563          ------------------------------------------------------------------------------
4CL-like7-genome_annotation TCCTCTCCCCGGCATCCCTCCACATCCCCGTCCTCTACTTCTCTCTCCTCTCTCTAGGCGTCGCCGTCTCCCCCTCCA

4CL-like7-hybrid-seq        ACCCGCTCTCCTCCCCCGCCGACCTCGCCCACCAAATCCGCATCAGCCGACCCGCCGTCGTCTTCGCCACCTCAGAAA
4CL-like7-KF220563          ------------------------------------------------------------------------------
4CL-like7-genome_annotation ACCCGCTCTCCTCCCCCGCCGACCTCGCCCACCAAATCCGCATCAGCCGACCCGCCGTCGTCTTCGCCACCTCAGAAA

4CL-like7-hybrid-seq        CTTCCAAGAAACTCCCGCAGATCAATATCCCCATCATCATCCTCGACTCGCCGCGGTTTCTCTCGATGCTCAAATGCG
4CL-like7-KF220563          ------------------------------------------------------------------------------
4CL-like7-genome_annotation CTTCCAAGAAACTCCCGCAGATCAATATCCCCATCATCATCCTCGACTCGCCGCGGTTTCTCTCGATGCTCAAATGCG

4CL-like7-hybrid-seq        ACGCCTCCTCGAGCTCCGCCGCCGCGGATCAGTCCTCCACCGCGGTGATCCTCTACTCATCCGGCACCACCGGGAGGA
4CL-like7-KF220563          -----------------------------------------------------------------------------A
4CL-like7-genome_annotation ACGCCTCCTCGAGCTCCGCCGCCGCGGATCAGTCCTCCACCGCGGTGATCCTCTACTCATCCGGCACCACCGGGAGGA

4CL-like7-hybrid-seq        TGAAGGGAGTCCAGCTGACGCACCGGAATTTGATCGCGTTGGTGGCGGTGGTGTATCACAACAAGTTGGCTGAAGATG
```

```
4CL-like7-KF220563               TGAAGGGAGTCCAGCTGACGCACCGGAATTTGATCGCGTTGGTGGCGGTGGTGTATCACAACAAGTTGGCTGAAGATG
4CL-like7-genome_annotation TGAAGGGAGTCCAGCTGACGCACCGGAATTTGATCGCGTTGGTGGCGGTGGTGTATCACAACAAGTTGGCTGAAGATG

4CL-like7-hybrid-seq             AGAAATTGGCGGAGAAAAATGGCGGCGCTGCGGTGTCGATTCTGACGGTGCCGCTGTTTCATGTTTTCGGGTTCTTCA
4CL-like7-KF220563               AGAAATTGGCGGAGAAAAATGGCGGCGCTGCGGTGTCGATTCTGACGGTGCCGCTGTTTCATGTTTTCGGGTTCTTCA
4CL-like7-genome_annotation AGAAATTGGCGGAGAAAAATGGCGGCGCTGCGGTGTCGATTCTGACGGTGCCGCTGTTTCATGTTTTCGGGTTCTTCA

4CL-like7-hybrid-seq             AGGTGATCAAGGCGGCGGCGTTTGGGGAGAGCCTGGTGTTGATGGAGAGATTCGATTTCGTCAAAATGCTGGAGGCGG
4CL-like7-KF220563               AGGTGATCAAGGCGGCGGCGTTTGGGGAGAGCCTGGTGTTGATGGAGAGATTCGATTTCGTCAAAATGCTGGAGGCGG
4CL-like7-genome_annotation AGGTGATCAAGGCGGCGGCGTTTGGGGAGAGCCTGGTGTTGATGGAGAGATTCGATTTCGTCAAAATGCTGGAGGCGG

4CL-like7-hybrid-seq             TGGAGAGGTATAAGGTTACCTACATGCCGGTGACGCCGCCGCTGGTGGTTGCGATGGCCAAATCTGATTTGGTGGACG
4CL-like7-KF220563               TGGAGAGGTATAAGGTTACCTACATGCCGGTGACGCCGCCGCTGGTGGTTGCGATGGCCAAATCTGATTTGGTGGACG
4CL-like7-genome_annotation TGGAGAGGTATAAGGTTACCTACATGCCGGTGACGCCGCCGCTGGTGGTTGCGATGGCCAAATCTGATTTGGTGGACG

4CL-like7-hybrid-seq             GCTTCGACTTGAGCTCCCTGCAGGCTTTGGGCTGCGGCGGAGCGCCGCTCGGGAAGGAAGTTTGCGAGCGGTTTCGGC
4CL-like7-KF220563               GCTTCGACTTGAGCTCCCTGCAGGCTTTGGGCTGCGGCGGAGCGCCGCTCGGGAAGGAAGTTTGCGAGCGGTTTCGGC
4CL-like7-genome_annotation GCTTCGACTTGAGCTCCCTGCAGGCTTTGGGCTGCGGCGGAGCGCCGCTCGGGAAGGAAGTTTGCGAGCGGTTTCGGC

4CL-like7-hybrid-seq             AGAAATTTCCCCACGTCGAGATTTTACAGGGCTATGGTCTGACGGAGAACACGGGAGGGGCGACGCGAACAATGGGGC
4CL-like7-KF220563               AGAAATTTCCCCACGTCGAGATTTTACAGGGCTATGGTCTGACGGAGAACACGGGAGGGGCGACGCGAACAATGGGGC
4CL-like7-genome_annotation AGAAATTTCCCCACGTCGAGATTTTACAGGGCTATGGTCTGACGGAGAACACGGGAGGGGCGACGCGAACAATGGGGC

4CL-like7-hybrid-seq             CGGAGGAGGCGAAGAGATACGGATCTGCAGGGCGGCTGTTGGAGAACATGGAAGCTAAGATTGTTGATCCTAAGAGTG
4CL-like7-KF220563               CGGAGGAGGCGAAGAGATACGGATCTGCAGGGCGGCTGTTGGAGAACATGGAAGCTAAGATTGTTGATCCTAAGAGTG
4CL-like7-genome_annotation CGGAGGAGGCGAAGAGATACGGATCTGCAGGGCGGCTGTTGGAGAACATGGAAGCTAAGATTGTTGATCCTAAGAGTG

4CL-like7-hybrid-seq             GAGAGCCCTTACCTCCACCACAACGTGGAGAACTATGGCTACGAGGACCTTCAGTTATGAAAGGTTATGCAGGGGATG
4CL-like7-KF220563               GAGAGCCCTTACCTCCACCACAACGTGGAGAACTATGGCTACGAGGACCTTCAGTTATGAAAGGTTATGCAGGGGATG
4CL-like7-genome_annotation GAGAGCCCTTACCTCCACCACAACGTGGAGAACTATGGCTACGAGGACCTTCAGTTATGAA---------AGGGGATG

4CL-like7-hybrid-seq             ATGCAGCCACAGCTGCAGCCATAGATTCACAAGGCTGGTTAAAGACTGGAGATCTTTGCTACTTTGACCAAGATGGCT
4CL-like7-KF220563               ATGCAGCCACAGCTGCAGCCATAGATTCACAAGGCTGGTTAAAGACTGGAGATCTTTGCTACTTTGACCAAGATGGCT
4CL-like7-genome_annotation ATGCAGCCACAGCTGCAGCCATAGATTCACAAGGCTGGTTAAAGACTGGAGATCTTTGCTACTTTGACCAAGATGGCT

4CL-like7-hybrid-seq             TTCTCTTCATTGTTGATCGCCTTAAAGAATTGATCAAATACAAGGCCTATCAAGTTGCTCCGGCGGAGTTGGAACATT
4CL-like7-KF220563               TTCTCTTCATTGTTGATCGCCTTAAAGAATTGATCAAATACAAGGCCTATCAAGTTGCTCCGGCGGAGTTGGAACATT
4CL-like7-genome_annotation TTCTCTTCATTGTTGATCGCCTTAAAGAATTGATCAAATACAAGGCCTATCAAGTTGCTCCGGCGGAGTTGGAACATT

4CL-like7-hybrid-seq             TGCTTCAATCAATTCCGGAAATTGACGATGCAGCTGTGGTTCCGTATCCTGATGAAGAAGCCGGCGAGATTCCGATGG
4CL-like7-KF220563               TGCTTCAATCAATTCCGGAAATTGACGATGCAGCTGTGGTTCCGTATCCTGATGAAGAAGCCGGCGAGATTCCGATGG
4CL-like7-genome_annotation TGCTTCAATCAATTCCGGAAATTGACGATGCAGCTGTGGTTCCGTATCCTGATGAAGAAGCCGGCGAGATTCCGATGG

4CL-like7-hybrid-seq             CCTATGTGGTGAGAAAACAGGGCAGCACTATCACTGCAACTCAGATTATGGATTACATTGCAAAGCAGGTTGCTCCTT
```

4CL-like7-KF220563 CCTATGTGGTGAGAAAACAGGGCAGCACTATCACTGCAACTCAGATTATGGATTACATTGCAAAGCAGGTTGCTCCTT

4CL-like7-genome_annotation CCTATGTGGTGAGAAAACAGGGCAGCACTATCACTGCAACTCAGATTATGGATTACATTGCAAAGCAGGTTGCTCCTT

4CL-like7-hybrid-seq ACAAGAAAATACGACGTCTAGCATTTATCGACTCCATTCCGAAATCTCCGGCTGGGAAGATCCTCCGCCGAGAACTCG

4CL-like7-KF220563 ACAAGAAAATACGACGTCTAGCATTTATCGACTCCATTCCGAAATCTCCGGCTGGGAAGATCCTCCGCCGAGAACTCG

4CL-like7-genome_annotation ACAAGAAAATACGACGTCTAGCATTTATCGACTCCATTCCGAAATCTCCGGCTGGGAAGATCCTCCGCCGAGAACTCG

4CL-like7-hybrid-seq TTCTTCATGCCATTTCTTTCACCTCATCAAAATTGTGATTTCTTAGGAACTCACCCTATTTCTCCCAATTTTTTATGA

4CL-like7-KF220563 TTCTTCATGCCATTTCTTTCACCTCATCAAAATTGTGA--

4CL-like7-genome_annotation TTCTTCATGCCATTTCTTTCACCTCATCAAAATTGTGA--

4CL-like7-hybrid-seq TTATCATTAATGTTAATAACACATGCACGAAAGATAAATTGAGCTAGTTAAGAGGGTGTTTGGCTAAGTTTATTTTA

4CL-like7-KF220563 --

4CL-like7-genome_annotation --

4CL-like7-hybrid-seq AAGAACTAACACAAAGAACGTACGTTGTATGTGTTATTGATAGTTGAGCTAATTGAAATATTATTGAC

4CL-like7-KF220563 --

4CL-like7-genome_annotation --

#SmTAT1

SmTAT1-hybrid-seq ---TCTCAAACAAACAAAATATCTTCTCTCTAACCTCT

SmTAT1-DQ334606.1 GCGGATATACTCAACATGACTATCATGAAAACAAAATTGGAAGTCTCAAACAAACAAAATATCTTCTCTCTAACCTCT

SmTAT1-genome_annotation --

SmTAT1-hybrid-seq CTCTCTCCTCTCCACTCATTGAAGCAGCTGAGATTCGGAGTTCGGAATTCGTGATTCAGATCTCTCGATTCAGTTGCG

SmTAT1-DQ334606.1 CTCTCTCCTCTCCACTCATTGAAGCAGCTGAGATTCGGAGTTCGGAATTCGTGATTCAGATCTCTCGATTCAGTTGCG

SmTAT1-genome_annotation --

SmTAT1-hybrid-seq GCAATGGAGTTGCAGAATCCAGCGCAGGAGATCGACGCGCCGACTACCATCACCATTAAGGGGATTTTGGGGTTGTTG

SmTAT1-DQ334606.1 GCAATGGAGTTGCAGAATCCAGCGCAGGAGATCGACGCGCCGACTACCATCACCATTAAGGGGATTTTGGGGTTGTTG

SmTAT1-genome_annotation ---ATGGAGTTGCAGAATCCAGCGCAGGAGATCGACGCGCCGACTACCATCACCATTAAGGGGATTTTGGGGTTGTTG

SmTAT1-hybrid-seq ATGTCGAGTACGGATCCGAAGGAGAGCGGGAAGAGAGTAATTTCGCTGGGGATTGGGGATCCGACTGCGTATTCGTGC

SmTAT1-DQ334606.1 ATGTCGAGTACGGATCCGAAGGAGAGCGGGAAGAGAGTAATTTCGCTGGGGATTGGGGATCCGACTGCGTATTCGTGC

SmTAT1-genome_annotation ATGTCGAGTACGGATCCGAAGGAGAGCGGGAAGAGAGTAATTTCGCTGGGGATTGGGGATCCGACTGCGTATTCGTGC

SmTAT1-hybrid-seq TTCCACGCTAGTAATGCTGCTCAGGAAGGTGTTGTGGAGGCTCTGCGCTCCACCAAATTCAACGGCTACGCTCCAACT

SmTAT1-DQ334606.1 TTCCACGCTAGTAATGCGGCTCAGGAAGGTGTTGTGGAGGCTCTGCGCTCCACCAAATTCAACGGCTACGCTCCAACT

SmTAT1-genome_annotation TTCCACGCTAGTAATGCTGCTCAGGAAGGTGTTGTGGAGGCTCTGCGCTCCACCAAATTCAACGGCTACGCTCCAACT

SmTAT1-hybrid-seq GCTGGTCTTCCACAAACCAGAGAGGCAATCGCCGAGTATTTGTCACGAGATCTTCCCTACAAGCTACCGGCCGACTCT

SmTAT1-DQ334606.1 GCTGGTCTTCCACAAACCAGAGAGGCAATCGCCGAGTATTTGTCACGAGATCTTCCCTACAAGCTACCGGCCGACTCT

SmTAT1-genome_annotation GCTGGTCTTCCACAAACCAGAGAGGCAATCGCCGAGTATTTGTCACGAGATCTTCCCTACAAGCTACCGGCCGACTCT

```
SmTAT1-hybrid-seq          GTGTATGTCACAGCCGGCTGCACACAAGCCATTGAGATAGCATTGTCCGTTTTGGCTCGCCCCGGTGCTAATATCTTG
SmTAT1-DQ334606.1          GTGTATGTCACAGCCGGCTGCACACAAGCCATTGAGATAGCATTGTCCGTTTTGGCTCGCCCCGGTGCTAATATCTTG
SmTAT1-genome_annotation   GTGTATGTCACAGCCGGCTGCACACAAGCCATTGAGATAGCATTGTCCGTTTTGGCTCGCCCCGGTGCTAATATCTTG

SmTAT1-hybrid-seq          CTGCCGAGACCGTGTTTCCCGATATACGGGCTTTGCGCCTCGTTTAGAAATATCGAAGTCCGCTACTTCGATCTTCAC
SmTAT1-DQ334606.1          CTGCCGAGACCGTGTTTCCCGATATACGGGCTTTGCGCCTCGTTTAGAAATATCGAAGTCCGCTACTTCGATCTTCAC
SmTAT1-genome_annotation   CTGCCGAGACCGTGTTTCCCGATATACGGGCTTTGCGCCTCGTTTAGAAATATCGAAGTCCGCTACTTCGATCTTCAC

SmTAT1-hybrid-seq          CCCGAGCAGGGATGGGAGGTTGATCTCGATGCAGTAGCAGATTTGGCAGACCACAATACAGTGGCAATGGTGATCATC
SmTAT1-DQ334606.1          CCCGAGCAGGGATGGGAGGTTGATCTCGATGCAGTAGCAGATTTGGCAGACCACAATACAGTGGCAATGGTGATCATC
SmTAT1-genome_annotation   CCCGAGCAGGGATGGGAGGTTGATCTCGATGCAGTAGCAGATTTGGCAGACCACAATACAGTGGCAATGGTGATCATC

SmTAT1-hybrid-seq          AATCCTGGGAATCCATGTGGGAACGTCTACTCATATCAGCACCTAAAGAAGATTGCTGAGACAGCCAAGAGGCTTGGT
SmTAT1-DQ334606.1          AATCCTGGGAATCCATGTGGGAACGTCTACTCATATCAGCACCTAAAGAAGATTGCTGAGACAGCCAAGAGGCTTGGT
SmTAT1-genome_annotation   AATCCTGGGAATCCATGTGGGAACGTCTACTCATATCAGCACCTAAAGAAGATTGCTGAGACAGCCAAGAGGCTTGGT

SmTAT1-hybrid-seq          ATAGTGGTGATAGCCGATGAGGTCTACGGCCATCTGGCTTTCGGGGCGAACCCTTTTGTTCCGATGGGAATATTCGGG
SmTAT1-DQ334606.1          ATAGTGGTGATAGCCGATGAGGTCTACGGCCATCTGGCTTTCGGGGCGAACCCTTTTGTTCCGATGGGAATATTCGGG
SmTAT1-genome_annotation   ATAGTGGTGATAGCCGATGAGGTCTACGGCCATCTGGCTTTCGGGGCGAACCCTTTTGTTCCGATGGGAATATTCGGG

SmTAT1-hybrid-seq          TCAATTGCCCCGGTGGTGACGCTTGGATCTTTGTCTAAGAGATGGTTGGTTCCTGGCTGGCGCCTTGGTTGGTTGGTC
SmTAT1-DQ334606.1          TCAATTGCCCCGGTGGTGACGCTTGGATCTTTGTCTAAGAGATGGTTGGTTCCTGGCTGGCGCCTTGGTTGGTTGGTC
SmTAT1-genome_annotation   TCAATTGCCCCGGTGGTGACGCTTGGATCTTTGTCTAAGAGATGGTTGGTTCCTGGCTGGCGCCTTGGTTGGTTGGTC

SmTAT1-hybrid-seq          ATCAATGACCCTGATGGCAGTTTGATGAGCCCAAAGTTTGTGGAACGCATCAAGAAATACTGTGACATCTGCGGAGGT
SmTAT1-DQ334606.1          ATCAATGACCCTGATGGCAGTTTGATGAGCCCAAAGTTTGTGGAACGCATCAAGAAATACTGTGACATCTGCGGAGGT
SmTAT1-genome_annotation   ATCAATGACCCTGATGGCAGTTTGATGAGCCCAAAGTTTGTGGAACGCATCAAGAAATACTGTGACATCTGCGGAGGT

SmTAT1-hybrid-seq          CCTGCTACATTCATACAGGCTGCAGTTCCTGAAATTGTTGAGCAAACACAAGAAGTTTTCTTCAGGAAAACAATTAAC
SmTAT1-DQ334606.1          CCTGCTACATTCATACAGGCTGCAGTTCCTGAAATTGTTGAGCAAACACAAGAAGTTTTCTTCAGGAAAACAATTAAC
SmTAT1-genome_annotation   CCTGCTACATTCATACAGGCTGCAGTTCCTGAAATTGTTGAGCAAACACAAGAAGTTTTCTTCAGGAAAACAATTAAC

SmTAT1-hybrid-seq          ATACTGAAGCAAACCTCTGATATCTGTTACCAAAAGATTGAAGACATCAATGGCATCACTTGTCCAACAAAACCTAAG
SmTAT1-DQ334606.1          ATACTGAAGCAAACCTCTGATATCTGTTACCAAAAGATTGAAGACATCAATGGCATCACTTGTCCAACAAAACCTAAG
SmTAT1-genome_annotation   ATACTGAAGCAAACCTCTGATATCTGTTACCAAAAGATTGAAGACATCAATGGCATCACTTGTCCAACAAAACCTAAG

SmTAT1-hybrid-seq          GGAGCAATGGCTTTCATGGTGAAGCTGAATCTTTCCAGGATGAAAGATATTAGTGATGATATTGACTTCTGTTTCAAG
SmTAT1-DQ334606.1          GGAGCAATGGCTTTCATGGTGAAGCTGAATCTTTCCAGGATGAAAGATATTAGTGATGATATTGACTTCTGTTTCAAG
SmTAT1-genome_annotation   GGAGCAATGGCTTTCATGGTGAAGCTGAATCTTTCCAGGATGAAAGATATTAGTGATGATATTGACTTCTGTTTCAAG

SmTAT1-hybrid-seq          CTGGCCAAAGAGGAATCTGTTATCATTCTTCCAGGGCTCGCTGTGGGTCTGAAGAACTGGATCCGGATCACATTTGCA
SmTAT1-DQ334606.1          CTGGCCAAAGAGGAATCTGTTATCATTCTTCCAGGGCTCGCTGTGGGTCTGAAGAACTGGATCCGGATCACATTTGCA
SmTAT1-genome_annotation   CTGGCCAAAGAGGAATCTGTTATCATTCTTCCAGGGCTCGCTGTGGGTCTGAAGAACTGGATCCGGATCACATTTGCA
```

```
SmTAT1-hybrid-seq         GTAGACGTACCTGCTCTTGAAGAAGCCATGGAGAGGCTCAAGTCTTTCTGTGAACGGCACTTCCTACTAAGACGCGTA
SmTAT1-DQ334606.1         GTAGACGTGCCTGCTCTTGAAGAAGCCATGGAGAGGCTCAAGTCTTTCTGTGAACGGCAC-TCCTACTAAGTCGCGTA
SmTAT1-genome_annotation  GTAGACGTGCCTGCTCTTGAAGAAGCCATGGAGAGGCTCAAGTCTTTCTGTGAACGGCAT-TCCTACTAA--------

SmTAT1-hybrid-seq         AAAATGGTAGCCGTTGAAGGAGTTGGATAGCCAAGATGATGTATTGCTCTCCTCGAGTAAATATTGTATCATCGATCG
SmTAT1-DQ334606.1         AAAATGGTAGCCGTTGAAGGAGTTGGATAGCCAAGATGATGTATTGCTCTCCTCGAGTAAATATTGTATCATCGATCG
SmTAT1-genome_annotation  --------------------------------------------------------------------------------

SmTAT1-hybrid-seq         CATTTTGGAACAATGTATTGCATTTAATTCGTTTATTTGAATAAGTCAGTTTGAGATTGTTAATTTATTATGTTCAAT
SmTAT1-DQ334606.1         CATTTTGGAACAATGTATTGCATTTAATTCGTTTATTTGAATAAGTCAGTTTGAGATTGTTAATTTATTATGTTCAAT
SmTAT1-genome_annotation  --------------------------------------------------------------------------------

SmTAT1-hybrid-seq         TTGTAATTTGCCCCGAAAAGATTGG--------------------
SmTAT1-DQ334606.1         TTGTAATTTGCCCCGAAAAAAAAAAAAAAAAAAAAAAAAAAAAAA
SmTAT1-genome_annotation  ---------------------------------------------
```

#SmTAT2

```
SmTAT2-hybrid-seq         --------------------------------------------------------------------------------
SmTAT2-KF220575           ATGCCAAATTCTACTGAGAGAGAGAGAGAAAGGGAGCTGTCGATGGAGAATGGAGGTTCGGCGGCGGCGCGGTGGCGT
SmTAT2-genome_annotation  ATGCCAAATTCTACTGAGAGAGAGAGAGAAAGGGAGCTGTCGATGGAGAATGGAGGTTCGGCGGCGGCGCGGTGGCGT

SmTAT2-hybrid-seq         --------------------------------------------------------------------------------
SmTAT2-KF220575           TTCGAGGGGAACGAGCGGCTCATCCTGGCCGGAGGAGTCACCGTTAGGGGCGTGCTCAACACTGTTATTGGAAATCTC
SmTAT2-genome_annotation  TTCGAGGGGAACGAGCGGCTCATCCTGGCCGGAGGAGTCACCGTTAGGGGCGTGCTCAACACTGTTATTGGAAATCTC

SmTAT2-hybrid-seq         -----------------------------------------GGAGATCCATCCGCCTTCCCTTCCTTCCGCACCTCTCCT
SmTAT2-KF220575           GACGGAAACGATACCAGACCGGTGATTCCGCTCGGCCACGGAGATCCATCCGCCTTCCCTTCCTTCCGCACCTCTCCT
SmTAT2-genome_annotation  GACGGAAACGATACCAGACCGGTGATTCCGCTCGGCCACGGAGATCCATCCGCCTTCCCTTCCTTCCGCACCTCTCCT

SmTAT2-hybrid-seq         TTTGCGGAGGACGCCGTCTGCTCCGCTGTCCGCTCCACCAAGTTCAACGGTTACTCCTCCACCGTCGGTATTCCCGCG
SmTAT2-KF220575           TTTGCGGAGGACGCCGTCTGCTCCGCTGTCCGCTCCACCAAGTTCAACGGTTACTCCTCCACCGTCGGTATTCCCGCG
SmTAT2-genome_annotation  TTTGCGGAGGACGCCGTCTGCTCCGCTGTCCGCTCCACCAAGTTCAACGGTTACTCCTCCACCGTCGGTATCCCCGCG

SmTAT2-hybrid-seq         GCTCGCAGTGCTGTAGCAGAGTATCTTTCTAAAGACCTTCCTTACAAGTTATCACCTGATGATGTTTTCCTGACCATT
SmTAT2-KF220575           GCTCGCAGTGCTGTAGCAGAGTATCTTTCTAAAGACCTTCCTTACAAGTTATCACCTGATGATGTTTTCCTGACCATT
SmTAT2-genome_annotation  GCTCGCAGTGCTGTAGCAGAGTATCTTTCTAAAGACCTTCCTTACAAGTTATCACCTGATGATGTTTTCCTGACCATT

SmTAT2-hybrid-seq         GGATGCACCCAAGCTTTAGAAGCCGTTGTGACTGTCCTTGCTCGTCCGGGTGCTAACCTTTTGCTTCCGAGGCCAGGC
SmTAT2-KF220575           GGATGCACCCAAGCTTTAGAAGCCGTTGTGACTGTCCTTGCTCGTCCGGGTGCTAACCTTTTGCTTCCGAGGCCAGGC
SmTAT2-genome_annotation  GGATGCACCCAAGCTTTAGAAGCCGTTGTGACTGTCCTTGCTCGTCCGGGTGCTAACCTTTTGCTTCCGAGGCCAGGC

SmTAT2-hybrid-seq         TTCCCTTACTACGAAGCCAGGGCTGGCTTTTGTAGTCTTGAATTCCGTCACTTTGATCTTCTCCCGGAACAAGATTGG
SmTAT2-KF220575           TTCCCTTACTACGAAGCCAGGGCTGGCTTTTGTAGTCTTGAATTCCGTCACTTTGATCTTCTCCCGGAACAAGATTGG
SmTAT2-genome_annotation  TTCCCTTACTACGAAGCCAGGGCTGGCTTTTGTAGTCTTGAATTCCGTCACTTTGATCTTCTCCCGGAACAAGATTGG
```

```
SmTAT2-hybrid-seq          GAAGTGGATTTGGCTTCGGTTGAAGCTCTGGCTGATGAAAATACAGTTGCTATGGTTATTATAAATCCGGGCAATCCA
SmTAT2-KF220575            GAAGTGGATTTGGCTTCGGTTGAAGCTCTGGCTGATGAAAATACAGTTGCTATGGTTATTATAAATCCGGGCAATCCA
SmTAT2-genome_annotation   GAAGTGGATTTGGCTTCGGTTGAAGCTCTGGCTGATGAAAATACAGTTGCTATGGTTATTATAAATCCGGGCAATCCA

SmTAT2-hybrid-seq          TGTGGAAATGTTTTCAAGTATGAACACTTGAAAAAGGTCGCGGAGACAGCTAGAAAGCTTGGAATCCTAGTGATCTCG
SmTAT2-KF220575            TGTGGAAATGTTTTCAAGTATGAACACTTGAAAAAGGTCGCGGAGACAGCTAGAAAGCTTGGAATCCTAGTGATCTCG
SmTAT2-genome_annotation   TGTGGAAATGTTTTCAAGTATGAACACTTGAAAAAGGTCGCGGAGACAGCTAGAAAGCTTGGAATCCTAGTGATCTCG

SmTAT2-hybrid-seq          GATGAAGTGTATGACCATCTTACCTTTGGTAGCAACCCCTTTGTCCCAATGGGAGTCTTTGCATCAATTGCCCCGATC
SmTAT2-KF220575            GATGAAGTGTATGACCATCTTACCTTTGGTAGCAACCCCTTTGTCCCAATGGGAGTCTTTGCATCAATTGCCCCGATC
SmTAT2-genome_annotation   GATGAAGTGTATGACCATCTTACCTTTGGTAGCAACCCCTTTGTCCCAATGGGAGTCTTTGCATCAATTGCCCCGATC

SmTAT2-hybrid-seq          TTGACTCTTGGATCAATATCTAAGAGATGGATTGTCCCGGGTTGGAGACTTGGCTGGCTTGTGACAAACGATCCCGAT
SmTAT2-KF220575            TTGACTCTTGGATCAATATCTAAGAGATGGATCGTCCCGGGTTGGAGACTTGGCTGGCTTGTGACAAACGATCCCGAT
SmTAT2-genome_annotation   TTGACTCTTGGATCAATATCTAAGAGATGGATCGTCCCGGGTTGGAGACTTGGCTGGCTTGTGACAAACGATCCCGAT

SmTAT2-hybrid-seq          GGGATCCTTACTAAGCAAGGGATTGTTGACAGCATCAAAGGTTTCCTGAATATTTCCGCTGACCCAGCAACCTTTATG
SmTAT2-KF220575            GGTATCCTTACTAAGCAAGGGATTGTTGACAGCATCAAAGGTTTCCTGAATATTTCCGCTGACCCAGCAACCTTTATG
SmTAT2-genome_annotation   GGTATCCTTACTAAGCAAGGGATTGTTGACAGCATCAAAGGTTTCCTGAATATTTCCTCTGACCCAGCAACCTTTATG

SmTAT2-hybrid-seq          CAGGGGGCAGTTCCACAGATTCTTGAGAATACCCCGAGCGACTTCTTTCAGAAAATTGTTAGTACACTTAGAGAAACT
SmTAT2-KF220575            CAGGGGGCAGTTCCACAGATTCTTGAGAATACCCCGAGCGACTTCTTTCAGAAAATTGTTAGTACACTTAGAGAAACT
SmTAT2-genome_annotation   CAGGGGGCAGTTCCACAGATTCTTGAGAATACCCCGAGCGACTTCTTTCAGAAAATTGTTAGTACACTTAGAGAAACT

SmTAT2-hybrid-seq          GCAGACATATGCTATGAACGCAGCAAAGAAATTCCCTGCATAACTTGCCCAAGCAGGCCTGAAGGAT-CATGTTTGCT
SmTAT2-KF220575            GCAGACATATGCTATGAACGCAGCAAAGAAATTCCCTGCATAACTTGCCCAAGCAGGCCTGAAGGATCCATGTTTGCT
SmTAT2-genome_annotation   GCAGACATATGCTATGAACGCAGCAAAGAAATTCCCTGCATAACTTGCCC-AGCAGACCTGAAGGATCCAT-------

SmTAT2-hybrid-seq          ATGGTGAAGCTCAATCTGTCTCTCCTCGAAGGCATTGAGGATGACATGGACTTCTGCTGCAAGCTCGCCAAAGAGGAA
SmTAT2-KF220575            ATGGTGAAGCTCAATCTGTCTCTCCTCGAAGGCATTGAGGATGACATGGACTTCTGCTGCAAGCTCGCCAAAGAGGAA
SmTAT2-genome_annotation   -------------------------------GCATTGAGGATGACATGGACTTCTGCTGCAAGCTCGCCAAAGAGGAA

SmTAT2-hybrid-seq          TCCGTGATACTCCTCCCAGGTTTTGCTGTAGGGCTCAAGAATTGGTTACGCGTTACATTTGCCATCGAACCATCATCT
SmTAT2-KF220575            TCCGTGATACTCCTCCCAGGTTTTGCTGTAGGGCTCAAGAATTGGTTACGCGTTACATTTGCCATCGAACCATCATCT
SmTAT2-genome_annotation   TCCGTGATACTCCTCCCAGGTTTTGCTGTAGGGCTCAAGAATTGGTTACGCGTTACATTTGCCATCGAACCATCATCT

SmTAT2-hybrid-seq          CTCGATGATGGCTTTCTCAGAATAAAAGCTTTTCATCAAAGGCACGCCAAGAAACAATGAAAGTTGTTTCTCTTAAGA
SmTAT2-KF220575            CTCGATGATGGCTTTCTCAGAATAAAAGCTTTTCATCAAAGGCACGCCAAGAAACAATGA------------------
SmTAT2-genome_annotation   CTCGATGATGGCTTCCTCAGAATAAAAGCTTTTCATCAAAGGCACGCCAAGAAACAATGA------------------

SmTAT2-hybrid-seq          GTTGTGATTCATTTGAAAAAACCATGATCGTTGTGATGTGAAACATCGTGTTTCATCTCATATTTTCTGTGCATGATT
SmTAT2-KF220575            ------------------------------------------------------------------------------
SmTAT2-genome_annotation   ------------------------------------------------------------------------------
```

```
SmTAT2-hybrid-seq          GCTTGTTTAGACATTGAAAGATTAATGATGTGTGTAATGTTTGTAGATTGATTGTAAGAAAATGGGATATGATGAAAC
SmTAT2-KF220575            --------------------------------------------------------------------------------
SmTAT2-genome_annotation   --------------------------------------------------------------------------------

SmTAT2-hybrid-seq          GCCGTGTTTCATCTTATATTTCCATGATTAATGTTTGTTTACATTGAAGGAGAAATAATGTGTAGAGTTTGTAGATTG
SmTAT2-KF220575            --------------------------------------------------------------------------------
SmTAT2-genome_annotation   --------------------------------------------------------------------------------

SmTAT2-hybrid-seq          ATTGTAAGAAAATGAAACATGTC
SmTAT2-KF220575            -----------------------
SmTAT2-genome_annotation   -----------------------
```

#SmHPPR1

```
SmHPPR1-hybrid-seq          ----------CTCAAACACCATCTCCCTCCCCCGCCACCAACGCCGCCGCTGCCGCGAAAATGGAGGCGATCGGTGTT
SmHPPR1-DQ099741.1          GCGGGGACCACTCAAACACCATCTCCCTCCCCCGCCACCAACGCCGCCGCTGCCGCGAAAATGGAGGCGATCGGTGTT
SmHPPR1-genome_annotation   --------------------------------------------------------------ATGGAGGCGATCGGTGTT

SmHPPR1-hybrid-seq          CTGATGATGTGCCCCATGAACAGCTACTTGGAGCAAGAGCTCGACAAGCGGTTCAAGCTCTTCCGCTACTGGACCCAG
SmHPPR1-DQ099741.1          CTGATGTTGTGCCCCATGAACAGCTACTTGGAGCAAGAGCTCGACAAGCGGTTCAAGCTCTTCCGCTACTGGACCCAG
SmHPPR1-genome_annotation   CTGATGATGTGCCCCATGAACAGCTACTTGGAGCAAGAGCTCGACAAGCGGTTCAAGCTCTTCCGCTACTGGACCCAG

SmHPPR1-hybrid-seq          CCGAAGCAGCGGGAATTCCTCGCTCAGCAGGCCGAGTCGATCCGCGCGATTGTCGGGAACTCCACCTACGGCGCCGAC
SmHPPR1-DQ099741.1          CCGAAGCAGCGGGAATTCCTCGCTCAGCAGGCCGAGTCGATCCGCGCGATTGTCGGGAACTCCAACTCCGGCGCCGAC
SmHPPR1-genome_annotation   CCGAAGCAGCGGGAATTCCTCGCTCAGCAGGCCGAGTCGATCCGCGCGATTGTCGGGAACTCCAACTCCGGCGCCGAC

SmHPPR1-hybrid-seq          GCCGATATCATCGACTCACTGCCGAAATTGGAGATAGTTTCGAGCTTCAGCGTGGGATTGGACAGAATCGACTTGCTC
SmHPPR1-DQ099741.1          GCCGATATCATCGACTCACTGCCGAAATTGGAGATAGTTTCGAGCTTCAGCGTGGGATTGGACAGAATCGACTTGCTC
SmHPPR1-genome_annotation   GCCGATATCATCGACTCACTGCCGAAATTGGAGATAGTTTCGAGCTTCAGCGTGGGATTGGACAGAATCGACTTGCTC

SmHPPR1-hybrid-seq          CAGTGTAAGGAAAAGGGGATTAGGGTTACCAACACGCCCGATGTGCTGACGGAAGACGTCGCGGATTTGGCGATCGGG
SmHPPR1-DQ099741.1          AAGTGTAAGGAAAAGGGGATTAGGGTTACCAACACGCCCGATGTGCTGACGGAAGACGTCGCGGATTTGGCGATCGGG
SmHPPR1-genome_annotation   CAGTGTAAGGAAAAGGGGATTAGGGTTACCAACACGCCCGATGTGCTGACGGAAGACGTCGCGGATTTGGCGATCGGG

SmHPPR1-hybrid-seq          TTGATGCTGGCGGTTCTGAGGCGGATTTGCGAGTGCGACAAGTATGTGAGGAGCGGGGCGTGGAAATTAGGAGACTTC
SmHPPR1-DQ099741.1          TTGATGCTGGCGGTTCTGAGGCGGATTTGCGAGTGCGACAAGTATGTGAGGAGCGGGGCGTGGAAATTAGGAGACTTC
SmHPPR1-genome_annotation   TTGATGCTGGCGGTTCTGAGGCGGATTTGCGAGTGCGACAAGTATGTGAGGAGCGGGGCGTGGAAATTAGGAGACTTC

SmHPPR1-hybrid-seq          AAGTTGACTACTAAGTTCAGTGGCAAAAGAGTTGGCATCATAGGATTGGGCAGAATCGGGTTAGCAATTGCTGAGCGA
SmHPPR1-DQ099741.1          AAGTTGACTACTAAGTTCAGTGGCAAAAGAGTTGGCATCATAGGATTGGGCAGAATCGGGTTAGCAGTTGCTGAGCGA
SmHPPR1-genome_annotation   AAGTTGACTACTAAGTTCAGTGGCAAAAGAGTTGGCATCATAGGATTGGGCAGAATCGGGTTAGCAATTGCTGAGCGA

SmHPPR1-hybrid-seq          GCAGAGGCATTCGATTGTCCCATCAATTACTACTCAAGATCCAAGAAAGCCAACACAAACTACACGTACTATGGCAGC
SmHPPR1-DQ099741.1          GCAGAGGCATTCGATTGTCCCATCAATTACTACTCAAGATCCAAGAAAGCCAACACAAACTACACGTACTATGGCAGC
```

SmHPPR1-genome_annotation GCAGAGGCATTCGATTGTCCCATCAATTACTACTCAAGATCCAAGAAAGCCAACACAAACTACACGTACTATGGCAGC

SmHPPR1-hybrid-seq GTTGTTGAATTGGCATCAAACAGCGACATCCTAGTGGTAGCATGTGCCCTGACTCCAGAAACAACCCACATTGTGAAC

SmHPPR1-DQ099741.1 GTTGTTGAATTGGCATCAAACAGCGATATCCTAGTGGTAGCATGTGCCCTGACTCCAGAAACAACCCACATTGTGAAC

SmHPPR1-genome_annotation GTTGTTGAATTGGCATCAAACAGCGACATCCTAGTGGTAGCATGTGCCCTGACTCCAGAAACAACCCACATTGTGAAC

SmHPPR1-hybrid-seq CGGGAAGTAATGGATGCACTGGGTCCGAAGGGAGTTCTGATCAACGTTGGACGGGGACCCCATGTTGATGAGGCCGAA

SmHPPR1-DQ099741.1 CGGGAAGTAATGGATGCACTGGGTCCGAAGGGAGTTCTGATCAACATTGGACGGGGACCCCATGTTGATGAAGCCGAA

SmHPPR1-genome_annotation CGGGAAGTAATGGATGCACTGGGTCCGAAGGGAGTTCTGATCAACGTTGGACGGGGACCCCATGTTGATGAGGCCGAA

SmHPPR1-hybrid-seq CTGGTGTCAGCTCTTGTGGAGGGTCGTCTGGGTGGCGCTGGACTTGATGTCTTTGAAAAGGAACCCGAGGTGCCCGAG

SmHPPR1-DQ099741.1 CTGGTGTCAGCTCTTGTGGAGGGTCGTCTGGGTGGCGCTGGACTTGATGTCTTTGAAAAGGAACCCGAGGTGCCCGAG

SmHPPR1-genome_annotation CTGGTGTCAGCTCTTGTGGAGGGTCGTCTGGGTGGCGCTGGACTTGATGTCTTTGAAAAGGAACCCGAGGTGCCCGAG

SmHPPR1-hybrid-seq CAACTGTTTGGCCTCGAAAACGTAGTTCTGTTGCCACATGTGGGGAGCGGCACCGTGGAAACGCGTAAAGTCATGGCT

SmHPPR1-DQ099741.1 CAACTGTTTGGCCTCGAAAACGTAGTTCTGTTGCCACATGTGGGGAGCGGCACCGTGGAAACGCGTAAAGTCATGGCT

SmHPPR1-genome_annotation CAACTGTTTGGCCTCGAAAACGTAGTTCTGTTGCCACATGTGGGGAGCGGCACCGTGGAAACGCGTAAAGTCATGGCT

SmHPPR1-hybrid-seq GACCTTGTTCTGGGAAATTTGGAAGCTCACTTTTCCAGCAAGCCTCTGTTAACACCTGTGGTTTGATAACTCTTTGAA

SmHPPR1-DQ099741.1 GACCTTGTTCTGGGAAATTTGGAAGCTCACTTTTCCAGCAAGCCTCTGTTAACACCTGTGGTTTGATAACTCTTTGAA

SmHPPR1-genome_annotation GACCTTGTTCTGGGAAATTTGGAAGCTCACTTTTCCAGCAAGCCTCTGTTAACACCTGTGGTTTGA------------

SmHPPR1-hybrid-seq AGCCGTTGATCCCATCTATTGAGAGGCACGCATGTTTGGTATCCTCAGTCAGTGATGAATTCATATTTTGGGTCTTGT

SmHPPR1-DQ099741.1 AGCTGTTGATCCCATCTATTGAGAGGCACGCATGTTTGGTATCCTCAGTCAGTGATGAATTCATGTTTTGGGTCTTGT

SmHPPR1-genome_annotation --

SmHPPR1-hybrid-seq TAATTGTTTCATTCAAATAAAAAAAACATCAATT

SmHPPR1-DQ099741.1 TAATTGTTTCATTCAAATAAAAAAA---------

SmHPPR1-genome_annotation ----------------------------------

#SmHPPR2

SmHPPR2-hybrid-seq ATTTCTTGATTCGAGCAGTGTTTTTTCATTTCTTATGTATATGTGGTTGTATCAAACCACAAGATTTCAATCACAAAT

SmHPPR2-KF220565 --

SmHPPR2-genome_annotation --

SmHPPR2-hybrid-seq TCGAATCGTGCATATAGCTTTATATATCTAAAGGATTTCTTGATTCAAGCAGTGTTTTTTTTTCATTTTGCAGAGCGA

SmHPPR2-KF220565 --

SmHPPR2-genome_annotation --

SmHPPR2-hybrid-seq AATGGAGAATGTGGGAGTGATGATGACCACTCCAATGTCAGCCTATCTGGAGCAACAGCTCCAGCAGCGCTTCACACT

SmHPPR2-KF220565 -ATGGAGAATGTGGGAGTGATGATGACCACTCCAATGTCAGCCTATCTGGAGCAACAGCTCCAGCAGCGCTTCACACT

SmHPPR2-genome_annotation -ATGGAGAATGTGGGAGTGATGATGACCACTCCAATGTCAGCCTATCTGGAGCAACAGCTCCAGCAGCGCTTCACACT

SmHPPR2-hybrid-seq CTTCAAGCTTTGGGAATCGCCCTCCCGAACTCAGTTCCTCACTCGGCGCGCCGACTCAGTCAATGCGGTGGTCGGCGA

```
SmHPPR2-KF220565           CTTCAAGCTTTGGGAATCGCCCTCCCGAACTCAGTTCCTCACTCGGCGCGCCGACTCAGTCAATGCGGTGGTCGGCGA
SmHPPR2-genome_annotation  CTTCAAGCTTTGGGAATCGCCCTCCCGAACTCAGTTCCTCACTCGGCGCGCCGACTCAGTCAATGCGGTGGTCGGCGA

SmHPPR2-hybrid-seq         CACCAAAATCGGCGCCGATTCCGAGCTGATTGACTCGCTCCCCCGCCTCGAGATCGTCTCCACTTACAGCGTCGGCCT
SmHPPR2-KF220565           CACCAAAATCGGCGCCGATTCCGAGCTGATTGACTCGCTCCCCCGCCTCGAGATCGTCTCCACTTACAGCGTCGGCCT
SmHPPR2-genome_annotation  CACCAAAATCGGCGCCGATTCCGAGCTGATTGACTCGCTCCCCCGCCTCGAGATCGTCTCCACTTACAGCGTCGGCCT

SmHPPR2-hybrid-seq         CGATAAAATCGACTTGGACAAGTGCCGAGAGAGAGGAATTAGGGTTACCAACACCCCCGACGTGCTGACGGACGACGT
SmHPPR2-KF220565           CGATAAAATCGACTTGGACAAGTGCCGAGAGAGAGGAATTAGGGTTACCAACACCCCCGACGTGCTGACGGACGACGT
SmHPPR2-genome_annotation  CGATAAAATCGACTTGGACAAGTGCCGAGAGAGAGGAATTAGGGTTACCAACACCCCCGACGTGCTGACGGACGACGT

SmHPPR2-hybrid-seq         CGCCGACCTCGCGATTGGGCTGGCTCTGACCACGTTGAGGAGAATCTGCGCCTGCGATGCGTTTGTGAGGAATGGATC
SmHPPR2-KF220565           CGCCGACCTCGCGATTGGGCTGGCTCTGACCACGTTGAGGAGAATCTGCGCCTGCGATGCGTTTGTGAGGAATGGATC
SmHPPR2-genome_annotation  CGCCGACCTCGCGATTGGGCTGGCTCTGACCACGTTGAGGAGAATCTGCGCCTGCGATGCGTTTGTGAGGAATGGATC

SmHPPR2-hybrid-seq         GTGGAGAAATGGGGATTTTCAGCTGGCTACCAAGTTCAGTGGCAAATCAGTCGGCATCCTCGGGCTAGGCAGGATTGG
SmHPPR2-KF220565           GTGGAGAAATGGGGATTTTCAGCTGGCTACCAAGTTCAGTGGCAAATCAGTCGGCATCCTCGGGCTAGGCAGGATTGG
SmHPPR2-genome_annotation  GTGGAGAAATGGGGATTTTCAGCTGGCTACCAAGTTCAGTGGCAAATCAGTCGGCATCCTCGGGCTAGGCAGGATTGG

SmHPPR2-hybrid-seq         TTCTGCCATTGCCAAAAGAGCTAAGGCATTTGGGTGCACCATCGGCTACCATTCCCGGACCAAGAAAC--ATCACCGA
SmHPPR2-KF220565           TTCTGCCATTGCCAAAAGAGCTAAGGCATTTGGGTGCACCATCGGCTACCATTCCCGGACCAAGAAACAAATCACCGA
SmHPPR2-genome_annotation  TTCTGCCATTGCCAAAAGAGCTAAGGCATTTGGGTGCACCATCGGCTACCATTCCCGGACCAAGAAACAAATCACCGA

SmHPPR2-hybrid-seq         CTACAGGTACCACTCCAATGCCGTTGATCTGGCTGCTAACTGTCGAATCCTGTTCGTGGCATGCTCCTTGACAGATGA
SmHPPR2-KF220565           CTACAGGTACCACTCCAATGCCGTTGATCTGGCTGCTAACTGTCGAATCCTGTTCGTGGCATGCTCCTTGACAGATGA
SmHPPR2-genome_annotation  CTACAGGTACCACTCCAATGCCGTTGATCTGGCTGCTAACTGTCGAATCCTGTTCGTGGCATGCTCCTTGACAGATGA

SmHPPR2-hybrid-seq         AACGAGACATATTGTGAATCGGGAGGTTATTGATGCATTGGGCCCTGGAGGGGTCCTTGTCAATGTAGGACGAGGCGC
SmHPPR2-KF220565           AACGAGACATATTGTGAATCGGGAGGTTATTGATGCATTGGGCCCTGGAGGGGTCCTTGTCAATGTAGGACGAGGCGC
SmHPPR2-genome_annotation  AACGAGACATATTGTGAATCGGGAGGTTATTGATGCATTGGGCCCTGGAGGGGTCCTTGTCAATGTAGGACGAGGCGC

SmHPPR2-hybrid-seq         ACTTGTTGATGAACATGAACTTGTGTCTGCACTGTGCGAAGGCAGGCTAGGTGGCGCTGGGCTCGACGTCTTTGAGAA
SmHPPR2-KF220565           ACTTGTTGATGAACATGAACTTGTGTCTGCACTGTGCGAAGGCAGGCTAGGTGGCGCTGGGCTCGACGTCTTTGAGAA
SmHPPR2-genome_annotation  ACTTGTTGATGAACATGAACTTGTGTCTGCACTGTGCGAAGGCAGGCTAGGTGGCGCTGGGCTCGACGTCTTTGAGAA

SmHPPR2-hybrid-seq         CGAACCTCACGTGCCCGAGCTGTTTTTCGAGCTAGTGAATGTAGTCCTGCTGCCTCATGTTGGAACCGACACCGTCGA
SmHPPR2-KF220565           CGAACCTCACGTGCCCGAGCTGTTTTTCGAGCTAGTGAATGTAGTCCTGCTGCCTCATGTTGGAACCGACACCGTCGA
SmHPPR2-genome_annotation  CGAACCTCACGTGCCCGAGCTGTTTTTCGAGCTAGTGAATGTAGTCCTGCTGCCTCATGTTGGAACCGACACCGTCGA

SmHPPR2-hybrid-seq         TACGAGCAACACTATGGCTGATCTCGTTGTTGCCAACTTGGAGTGTCATTTCCTCAAGAAACCGCTGTTGACTCCGGT
SmHPPR2-KF220565           GACGAGCAACACTATGGCTGATCTCGTTGTTGCCAACTTGGAGTGTCATTTCCTCAAGAAACCGCTGTTGACTCCGGT
SmHPPR2-genome_annotation  GACGAGCAACACTATGGCTGATCTCGTTGTTGCCAACTTGGAGTGTCATTTCCTCAAGAAACCGCTGTTGACTCCGGT

SmHPPR2-hybrid-seq         GATCTGAGCAGAATTAGCCATCTTTTGTCACCATTATGGCAAGTTCTTCTTTTGGTTGATTGATTTCGTAGGTGCAAA
```

```
SmHPPR2-KF220565           GATCTGA-----------------------------------------------------------------------
SmHPPR2-genome_annotation  GATCTGA-----------------------------------------------------------------------

SmHPPR2-hybrid-seq         AATTTGCATAGTTTTAGGGAAAGTGGCAGCATGTACAATTTTGTATCCTCATATTTATAGTTCTATAATCTCTAACTG
SmHPPR2-KF220565           ------------------------------------------------------------------------------
SmHPPR2-genome_annotation  ------------------------------------------------------------------------------

SmHPPR2-hybrid-seq         CTGATTGTGAA
SmHPPR2-KF220565           -----------
SmHPPR2-genome_annotation  -----------
```

#SmHCT1

```
SmHCT1-hybrid-seq          ------------------------------------------------------------------------------
SmHCT1-KF220570            ------------------------------------------------------------------------------
SmHCT1-genome_annotation   ATGAGAATGAATGTGAAAGAATCGACGATGGTAAAGCCAATGGCAGAAACGCCACGTGGGAGTCTGTGGCTGTCAAAT

SmHCT1-hybrid-seq          ------------------------TACCACAGCCGCTCCGTCTACTTGTACAGCTCCAATGGCGCCGCCAACTTCTTC
SmHCT1-KF220570            ------------------------------------------------------------------------------
SmHCT1-genome_annotation   TTGGACATACAAATACTTGGTAACTACCACAGCCGCTCCGTCTACTTGTACAGCTCCAATGGCGCCGCCAACTTCTTC

SmHCT1-hybrid-seq          GACGTTGGCTTGCTGAAGGCGGCTCTTGGTCGGGCTTTGGTTGACTTCTACCCCTATGCAGGGAGGCTGGAGAAGGCG
SmHCT1-KF220570            ------------------------------------------------------------------------------
SmHCT1-genome_annotation   GACGTTGGCTTGCTGAAGGCGGCTCTTGGTCGGGCTTTGGTTGACTTCTACCCCTATGCAGGGAGGCTGGAGAAGGCG

SmHCT1-hybrid-seq          GATGACGGGCGCCTCCAGATTAACTGCAACAGCGAGGGAGTGTTGTTCATGGTGGCGGAGTGCGACACCGCAGTCGAT
SmHCT1-KF220570            ------------------------------------------------ATGGTGGCGGAGTGCGACACCGCAGTCGAT
SmHCT1-genome_annotation   GATGACGGGCGCCTCCAGATTAACTGCAACAGCGAGGGAGTGTTGTTCATGGTGGCGGAGTGCGACACCGCAGTCGAT

SmHCT1-hybrid-seq          GACTTAGGTGATTTTGCCGCCCGCGCCCCCGACCTCTCCCTCGTCCCTAAAGTCGATTATTCCGGGGGGATTTCCACC
SmHCT1-KF220570            GACTTAGGTGATTTTGCCGCCCGCGCCCCCGACCTCTCCCTCGTCCCTAAAGTCGATTATTCCGGGGGGATTTCCACC
SmHCT1-genome_annotation   GACTTAGGTGATTTTGCCGCCCGCGCCCCCGACCTCTCCCTCGTCCCTAAAGTCGATTATTCCGGGGGGATTTCCACC

SmHCT1-hybrid-seq          TGGCCACTTTTGCTGCTTCAGTTGACTCGGTTCAAATGCGGTAGCATTTCCTTGGGCGTTACAGCCGACCACCACGTT
SmHCT1-KF220570            TGGCCACTTTTGCTGCTTCAGTTGACTCGGTTCAAATGCGGTAGCATTTCCTTGGGCGTTACAGCCGACCACCACGTT
SmHCT1-genome_annotation   TGGCCACTTTTGCTGCTTCAGTTGACTCGGTTCAAATGCGGTAGCATTTCCTTGGGCGTTACAGCCGACCACCACGTT

SmHCT1-hybrid-seq          AAAGATGGTACCTCCGGCCTCCATTTCATCAACACGTGGTCTGACATAGCCCGCGGCGTCACCTCTGTGGCCGTCCCC
SmHCT1-KF220570            AAAGATGGTACCTCCGGCCTCCATTTCATCAACACGTGGTCTGACATAGCCCGCGGCGTCACCTCTGTGGCCGTCCCC
SmHCT1-genome_annotation   AAAGATGGTACCTCCGGCCTCCATTTCATCAACACGTGGTCTGACATAGCCCGCGGCGTCACCTCTGTGGCCGTCCCC

SmHCT1-hybrid-seq          CCGGTCCTGGACCGCCGCCCCCTCTCGGCGCGGCAGCCACCGCAGCCCAGGTTCCCCCACGACGAATACCAGCCCCCG
SmHCT1-KF220570            CCGGTCCTGGACCGCCGCCCCCTCTCGGCGCGGCAGCCACCGCAGCCCAGGTTCCCCCACGACGAATACCAGCCCCCG
SmHCT1-genome_annotation   CCGGTCCTGGACCGCCGCCCCCTCTCGGCGCGGCAGCCACCGCAGCCCAGGTTCCCCCACGACGAATACCAGCCCCCG
```

```
SmHCT1-hybrid-seq            CCGCAGCTCATAACCCCTCTCCCCATCACCGACACATCA----CCGCGTCTCAACTCCCTCCGA--CACCTCCGCGCC
SmHCT1-KF220570              CCGCAGCTCATAACCCCTCTCCCCATCACCGACACATCACACGCAGTATTCAAACTCACTCCAGACCACCTCCGCGCC
SmHCT1-genome_annotation     CCGCAGCTCATAACCCCTCTCCCCATCACCGACACATCACACGCAGTATTCAAACTCACTCCAGACCACCTCCGCGCC

SmHCT1-hybrid-seq            ATCAAGCAGAGCTGCAGCGGCTATTCGACGTACCAGGCGGTCACCGGCCACGTGTGGCGCTGCGTCTGTGCGGCCCGC
SmHCT1-KF220570              ATCAAGCAGAGCTGCAGCGGCTATTCGACGTACCAGGCGGTCACCGGCCACGTGTGGCGCTGCGTCTGTGCGGCCCGC
SmHCT1-genome_annotation     ATCAAGCAGAGCTGCAGCGGCTATTCGACGTACCAGGCGGTCACCGGCCACGTGTGGCGCTGCGTCTGTGCGGCCCGC

SmHCT1-hybrid-seq            GGCCTCCCTCCAGACCAACCAACCAGGCTGAAATTCTCGGTGAACGGGCGTCCGAGGCTGCAGCCGCCCCTGCCGCCG
SmHCT1-KF220570              GGCCTCCCTCCAGACCAACCAACCAGGCTGAAATTCTCGGTGAACGGGCGTCCGAGGCTGCAGCCGCCCCTGCCGCCG
SmHCT1-genome_annotation     GGCCTCCCTCCAGACCAACCAACCAGGCTGAAATTCTCGGTGAACGGGCGTCCGAGGCTGCAGCCGCCCCTGCCGCCG

SmHCT1-hybrid-seq            GGTTTATTCGGCAACGTGAACTTCTATACCACGTCGACCGCTCTGTGCGGCGAACTGGTGTCGAACCCGCCGGGGTTC
SmHCT1-KF220570              GGTTTATTCGGCAACGTGAACTTCTATACCACGTCGACCGCTCTGTGCGGCGAACTGGTGTCGAACCCGCCGGGGTTC
SmHCT1-genome_annotation     GGTTTATTCGGCAACGTGAACTTCTATACCACGTCGACCGCTCTGTGCGGCGAACTGGTGTCGAACCCGCCGGGGTTC

SmHCT1-hybrid-seq            GCGGCGGAGAAAGTCAACGGGGCGGTGGCCCAAATGAACGACGAGTATTTAAGGTCGGCTATCGACTACTTGGAGGTG
SmHCT1-KF220570              GCGGCGGAGAAAGTCAACGGGGCGGTGGCCCAAATGAACGACGAGTATTTAAGGTCGGCTATCGACTACTTGGAGGTG
SmHCT1-genome_annotation     GCGGCGGAGAAAGTCAACGGGGCGGTGGCCCAAATGAACGACGAGTATTTAAGGTCGGCTATCGACTACTTGGAGGTG

SmHCT1-hybrid-seq            CAGCTGCCGAGTATCCTCAACACCACTGCCCGCAACGAAAACTTGGTGAAGTGTCCTAATCTTGATATTACGAGCTGG
SmHCT1-KF220570              CAGCTGCCGAGTATCCTCAACACCACTGCCCGCAACGAAAACTTGGTGAAGTGTCCTAATCTTGATATTACGAGCTGG
SmHCT1-genome_annotation     CAGCTGCCGAGTATCCTCAACACCACTGCCCGCAACGAAAACTTGGTGAAGTGTCCTAATCTTGATATTACGAGCTGG

SmHCT1-hybrid-seq            GTGCGGCTGCCCTTTTACGAAGGGGATTTCGGGTGGGGGAAGCCGGTTTATGCCGGGCCGGCAGTGGTTCAGTATGAA
SmHCT1-KF220570              GTGCGGCTGCCCTTTTACGAAGGGGATTTCGGGTGGGGGAAGCCGGTTTATGCCGGGCCGGCAGTGGTTCAGTATGAA
SmHCT1-genome_annotation     GTGCGGCTGCCCTTTTACGAAGGGGATTTCGGGTGGGGGAAGCCGGTTTATGCCGGGCCGGCAGTGGTTCAGTATGAA

SmHCT1-hybrid-seq            GGGAAGGCTTATTTGCTCCTTGATCCGGAGAGTGATGGGAGTTGGTTGCTTCAGATCACGCTCTTCAAGCCGCATATG
SmHCT1-KF220570              GGGAAGGCTTATTTGCTCCTTGATCCGGAGAGTGATGGGAGTTGGTTGCTTCAGATCACGCTCTTCAAGCCGCATATG
SmHCT1-genome_annotation     GGGAAGGCTTATTTGCTCCTTGATCCGGAGAGTGATGGGAGTTGGTTGCTTCAGATCACGCTCTTCAAGCCGCATATG

SmHCT1-hybrid-seq            GACGCCTTTCACAACTTGTTTTATGATATTCCTCCGTCCGCCAAAAACATCACAATTTGATTACCCACAACTTGTTTA
SmHCT1-KF220570              GACGCCTTTCACAACTTGTTTTATGATATTCCTCCGTCCGCCAAAAACATCACAATTTGA------------------
SmHCT1-genome_annotation     GACGCCTTTCACAACTTGTTTTATGATATTCCTCCGTCCGCCAAAAACATCACAATTTGA------------------

SmHCT1-hybrid-seq            TGTCTATGCTTTGATGTTGGTCTCGATTGTGTGTGTGTTGAGTCGAACCACTTTGTTTGTCGTATCTTTTATTTCAAA
SmHCT1-KF220570              --------------------------------------------------------------------------------
SmHCT1-genome_annotation     --------------------------------------------------------------------------------

SmHCT1-hybrid-seq            TAAACACTATTG
SmHCT1-KF220570              ------------
SmHCT1-genome_annotation     ------------
```

#SmCYP98A78

```
SmCYP98A78-hybrid-seq         AGGCAAGCTCATCCGCAGCAGCCATGGCAGCTCTCCTCCTCGCCCCCGTCGCCGCCGTCCTCGCCGTCGTCCTCTACC
SmCYP98A78-HQ316179.1         -----------------------ATGGCAGCTCTCCTCCTCGCCCCCGTCGCCGCCGTCCTCGCTGTCGTCCTCTACC
SmCYP98A78-genome_annotation -----------------------ATGGCAGCTCTCCTCCTCGCCCCCGTCGCCGCCGTCCTCGCCGTCGTCCTCTACC

SmCYP98A78-hybrid-seq         ATATCTACTACCGCCTCCGCTTCCGCCTCCCGCCGGGGCCGTTCCCGTGGCCGGTGGTGGGGAATCTGTACGACATCA
SmCYP98A78-HQ316179.1         ATATCTACTACCGCCTCCGCTTCCGCCTCCCGCCGGGGCCGTTCCCGTGGCCGGTGGTGGGGAATCTGTACGACATCA
SmCYP98A78-genome_annotation ATATCTACTACCGCCTCCGCTTCCGCCTCCCGCCGGGGCCGTTCCCGTGGCCGGTGGTGGGGAATCTGTACGACATCA

SmCYP98A78-hybrid-seq         AGCCCGTCCGCTTCCGCTGCTTCGCTGAGTGGGCCCAATCCTACGGCCCGATCCTCTCGGTTTGGTTCGGATCCACCC
SmCYP98A78-HQ316179.1         AGCCCGTCCGCTTCCGCTGCTTCGCTGAGTGGGCCCAATCCTACGGCCCGATCCTCTCGGTTTGGTTCGGATCCACCC
SmCYP98A78-genome_annotation AGCCCGTCCGCTTCCGCTGCTTCGCTGAGTGGGCCCAATCCTACGGCCCGATCCTCTCGGTTTGGTTCGGATCCACCC

SmCYP98A78-hybrid-seq         TCAACGTCGTCGTTTCCAACTCCGAGCTGGCTAAGGAGGTGCTGAAGGAGAAGGACGGCCAGCTGGCCGACCGCCACC
SmCYP98A78-HQ316179.1         TCAACGTCGTCGTTTCCAACTCCGAGCTGGCTAAGGAGGTGCTGAAGGAGAAGGACGGCCAGCTGGCCGACCGCCACC
SmCYP98A78-genome_annotation TCAACGTCGTCGTTTCCAACTCCGAGCTGGCTAAGGAGGTGCTGAAGGAGAAGGACGGCCAGCTGGCCGACCGCCACC

SmCYP98A78-hybrid-seq         GCAGCCGCTCCGCCGTCAAGCTCAGCAGAGACGGCCAGGATTTGATTTGGGCGGACTACGGGCCGCACTACGTGAAGG
SmCYP98A78-HQ316179.1         GCAGCCGCTCCGCCGTCAAGCTCAGCAGAGACGGCCAGGATTTGATTTGGGCGGACTACGGGCCGCACTACGTGAAGG
SmCYP98A78-genome_annotation GCAGCCGCTCCGCCGTCAAGCTCAGCAGAGACGGCCAGGATTTGATTTGGGCGGACTACGGGCCGCACTACGTGAAGG

SmCYP98A78-hybrid-seq         TCAGGAAGGTCTGTACCGTCGTCCTCTTCTCCCCCAAGCGCCTCGAGCTGCTCCGCCCGATTAGGGAAGATGAGATCA
SmCYP98A78-HQ316179.1         TCAGGAAGGTCTGTACCGTCGTCCTCTTCTCCCCCAAGCGCCTCGAGCTGCTCCGCCCGATTAGGGAAGATGAGATCA
SmCYP98A78-genome_annotation TCAGGAAGGTCTGTACCGTCGTCCTCTTCTCCCCCAAGCGCCTCGAGCTGCTCCGCCCGATTAGGGAAGATGAGATCA

SmCYP98A78-hybrid-seq         CCGCCATGGTTGAATCCATCTACAACGACTCCACTGCTTCTTCTGGCAAGAGCGTGGTGCTAAAGAAATACCTTGCAT
SmCYP98A78-HQ316179.1         CCGCCATGGTTGAATCCATCTACAACGACTCCACTGCTTCTTCTGGCAAGAGCGTGGTGCTAAAGAAATACCTTGCAT
SmCYP98A78-genome_annotation CCGCCATGGTTGAATCCATCTACAACGACTCCACTGCTTCTTCTGGCAAGAGCGTGGTGCTAAAGAAATACCTTGCAT

SmCYP98A78-hybrid-seq         CAATGGCGTTCCACAACATAACAAGGCTGGTATTTGGGAAAAGGTTCGTGAATTCGGAAGGGGCAGTGGACAAGCAAG
SmCYP98A78-HQ316179.1         CAATGGCGTTCCACAACATAACAAGGCTGGTATTTGGGAAAAGGTTCGTGAATTCGGAAGGGGCAGTGGACAAGCAAG
SmCYP98A78-genome_annotation CAATGGCGTTCCACAACATAACAAGGCTGGTATTTGGGAAAAGGTTCGTGAATTCGGAAGGGGCAGTGGACAAGCAAG

SmCYP98A78-hybrid-seq         GGCAGGAGTTCAAGGCCATTGCTATAAATGGGCTGAAGCTGGGCGCTTCCCTAGCCATGGCCGAGCACATCCCCTGGC
SmCYP98A78-HQ316179.1         GGCAGGAGTTCAAGGCCATTGCTATAAATGGGCTGAAGCTGGGCGCTTCCCTAGCCATGGCCGAGCACATCCCCTGGC
SmCYP98A78-genome_annotation GGCAGGAGTTCAAGGCCATTGCTATAAATGGGCTGAAGCTGGGCGCTTCCCTAGCCATGGCCGAGCACATCCCCTGGC

SmCYP98A78-hybrid-seq         TCCGTTGGGCCTTCCCCCTCGACGAGGACGCCTTCACCCAGCACGGAGCTCGCATGGAGCGCCTCACTCGAGAGATCA
SmCYP98A78-HQ316179.1         TCCGTTGGGCCTTCCCCCTCGACGAGGACGCCTTCACCCAGCACGGAGCTCGCATGGAGCGCCTCACTCGAGAGATCA
SmCYP98A78-genome_annotation TCCGTTGGGCCTTCCCCCTCGACGAGGACGCCTTCACCCAGCACGGAGCTCGCATGGAGCGCCTCACTCGAGAGATCA

SmCYP98A78-hybrid-seq         TGCAAGAGCACACCCTTCTCCGCCAGAAAACCGGAGGCGCCAAGAACCACTTCTTCGACGCCCTCCTCACCCTCAAGG
SmCYP98A78-HQ316179.1         TGCAAGAGCACACCCTTCTCCGCCAGAAAACCGGAGGCGCCAAGAACCACTTCTTCGACGCCCTCCTCACCCTCAAGG
SmCYP98A78-genome_annotation TGCAAGAGCACACCCTTCTCCGCCAGAAAACCGGAGGCGCCAAGAACCACTTCTTCGACGCCCTCCTCACCCTCAAGG
```

```
SmCYP98A78-hybrid-seq            ACGAGTACGACCTCAGCGAGGACACCATCATCGCCCTTCTTTGGGACATGATCGCGGCAGGAATGGACACCCCTGCGA
SmCYP98A78-HQ316179.1            ACGAGTACGACCTCAGCGAGGACACCATCATCGCCCTTCTTTGGGACATGATCGCGGCAGGAATGGACACCCCTGCAA
SmCYP98A78-genome_annotation ACGAGTACGACCTCAGCGAGGACACCATCATCGCCCTTCTTTGGGACATGATCGCGGCAGGAATGGACACCCCTGCGA

SmCYP98A78-hybrid-seq            TATCCGTGGAGTGGGCGATGGCGGAGCTGGTGAGGAATCCGAGGGTGCAACAGAAGGTGCAGGAGGAGCTGGACCGCG
SmCYP98A78-HQ316179.1            TATCCGTGGAGTGGGCGATGGCGGAGCTGGTGAGGAATCCGAGGGTGCAACAGAAGGTGCAGGAGGAGCTGGACCGCG
SmCYP98A78-genome_annotation TATCCGTGGAGTGGGCGATGGCGGAGCTGGTGAGGAATCCGAGGGTGCAACAGAAGGTGCAGGAGGAGCTGGACCGCG

SmCYP98A78-hybrid-seq            TGATAGGCCGTGATCGTGTGATGACGGAAGTGGACATCCCGAACCTGCCCTACTTGCAGTGTGTGGTGAAGGAGTCAT
SmCYP98A78-HQ316179.1            TGATAGGCCGTGATCGTGTGATGACGGAAGTGGACATCCCGAACCTGCCCTACTTGCAGTGTGTGGTGAAGGAGTCAT
SmCYP98A78-genome_annotation TGATAGGCCGTGATCGTGTGATGACGGAAGTGGACATCCCGAACCTGCCCTACTTGCAGTGTGTGGTGAAGGAGTCAT

SmCYP98A78-hybrid-seq            TGAGGTTGCACCCGCCCACCCCTCTCATGCTCCCGCACCGCGCCAACACCAATGTCAAGATCGGTGGCTACGACATCC
SmCYP98A78-HQ316179.1            TGAGGTTGCACCCGCCCACCCCTCTCATGCTCCCGCACCGCGCCAACACCAATGTCGAGATCGGTGGCTACGACATCC
SmCYP98A78-genome_annotation TGAGGTTGCACCCGCCCACCCCTCTCATGCTCCCGCACCGCGCCAACACCAATGTCAAGATCGGTGGCTACGACATCC

SmCYP98A78-hybrid-seq            CCAAGGGCTCCAACGTGAACGTGAACGTGTGGGCTGTAGCACGCGACCCGGCAGTGTGGAAGAACCCGTTGGAGTTCA
SmCYP98A78-HQ316179.1            CCAAGGGCTCCAACGTAAACGTGAACGTGTGGGCTGTAGCACGCGACCCGGCAGTGTGGAAGAACCCGTTGGAGTTCA
SmCYP98A78-genome_annotation CCAAGGGCTCCAACGTGAACGTGAACGTGTGGGCTGTAGCACGCGACCCGGCAGTGTGGAAGAACCCGTTGGAGTTCA

SmCYP98A78-hybrid-seq            GGCCGGAGAGGTTCGTGGAGGAAGATATTGATATAAAGGGGCATGATTTCCGGGTTCTGCCGTTTGGTGCCGGGAGAA
SmCYP98A78-HQ316179.1            GGCCGGAGAGGTTCGTGGAGGAAGGTATTGATATAAAGGGGCATGATTTCCGGGTTCTGCCGTTTGGTGCCGGGAGAA
SmCYP98A78-genome_annotation GGCCGGAGAGGTTCGTGGAGGAAGATATTGATATAAAGGGGCATGATTTCCGGGTTCTGCCGTTTGGTGCCGGGAGAA

SmCYP98A78-hybrid-seq            GAGTGTGCCCCGGGGCGCAGCTAGGGATTGACCTCACGACGTCGATGATAGGCCATCTGCTGCACCACTTCAGCTGGG
SmCYP98A78-HQ316179.1            GAGTGTGCCCCGGGGCGCAGCTAGGGATTGACCTCACCACGTCGATGATAGGCCATCTGCTGCACCACTTCAGCTGGG
SmCYP98A78-genome_annotation GAGTGTGCCCCGGGGCGCAGCTAGGGATTGACCTCACGACGTCGATGATAGGCCATCTGCTGCACCACTTCAGCTGGG

SmCYP98A78-hybrid-seq            CTCCTCCAGCCGGAATGAGAACGGAGGAGATCAACTTGGACGAGAATCCAGGTACCGTCACCTACATGAAGAATCCGG
SmCYP98A78-HQ316179.1            CTCCTCCAGCCGGAATGAGAACGGAGGAGATCAACTTGGACGAGAATCCAGGTACCGTCACCTACATGAAGAATCCGG
SmCYP98A78-genome_annotation CTCCTCCAGCCGGAATGAGAACGGAGGAGATCAACTTGGACGAGAATCCAGGTACCGTCACCTACATGAAGAATCCGG

SmCYP98A78-hybrid-seq            TGGAGGCGCTTCCGACGCCAAGACTAGCGCCTCACTTGTACAAGCGCGTTGCTGTCGACACCATTTGAGCTGTCTTCA
SmCYP98A78-HQ316179.1            TGGAGGCGCTTCCGACGCCAAGACTAGCGCCTCACTTGTACAAGCGCGTTGCTGTCGACACCATTTGAGCTGTTTTCA
SmCYP98A78-genome_annotation TGGAGGCGCTTCCGACGCCAAGACTAGCGCCTCACTTGTACAAGCGCGTTGCTGTCGACACCATTTGA----------

SmCYP98A78-hybrid-seq            TCATG-TTTTTGCTGCCTTTTCGTCCTCTACATTAGCTTTTGCCAAAATGTTACACGACTATTCTATTGAATTTTTCA
SmCYP98A78-HQ316179.1            TCATGTTTTTTGCTGCCTTTTCGTCCTCTACATTAGCTTTTGCCAAAATGTTACACGACTATTTTATTGAATTTTTCA
SmCYP98A78-genome_annotation ------------------------------------------------------------------------------

SmCYP98A78-hybrid-seq            CTTGTTGCAGAATAAGTTATTGCTAGCTATTTTGTAAGTGAAATGTATGTTGTTTAAGAGTTTGATTCAAACACCAAA
SmCYP98A78-HQ316179.1            CTTGTTGCAAAAAAAA---------------------------------------------------AAAAAAAAAAA
SmCYP98A78-genome_annotation ------------------------------------------------------------------------------
```

```
SmCYP98A78-hybrid-seq        GAATTTAA
SmCYP98A78-HQ316179.1        AAAAAAAA
SmCYP98A78-genome_annotation --------
```

Figure S1. The identified 15 full-length transcripts from hybrid-seq align with the gene from NCBI and genome annotation.

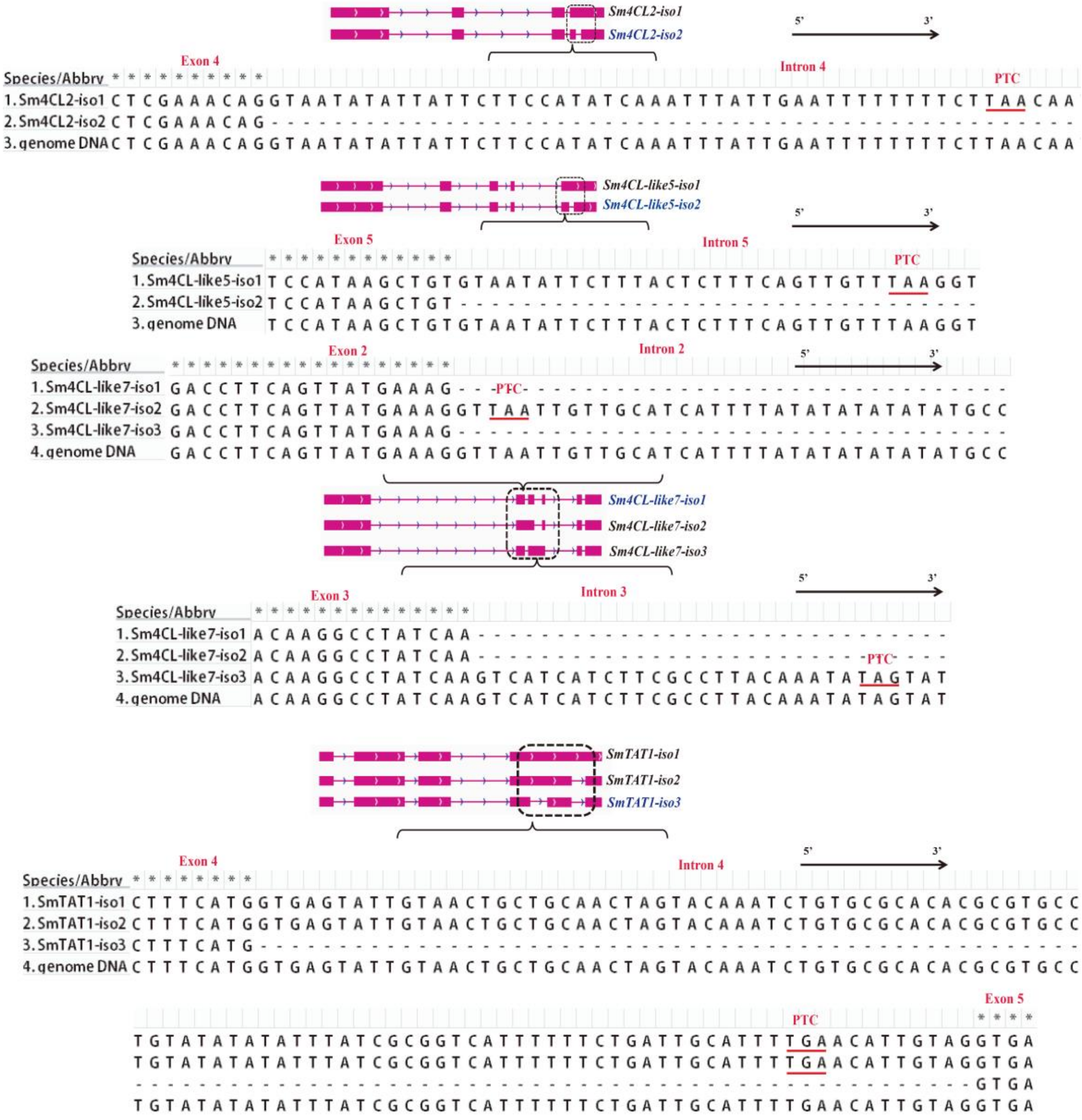

Figure S2. The alternative splicing events in rosmarinic acid biosynthetic genes introduce premature translation termination codons.

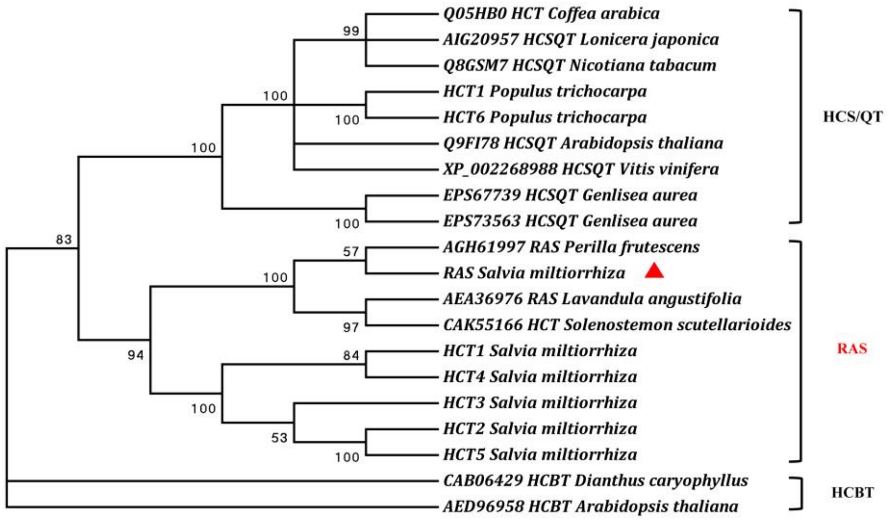

Figure S3. A phylogenetic tree constructed from 20 amino acid sequences of HCTs from *S. miltiorrhiza* and other species.

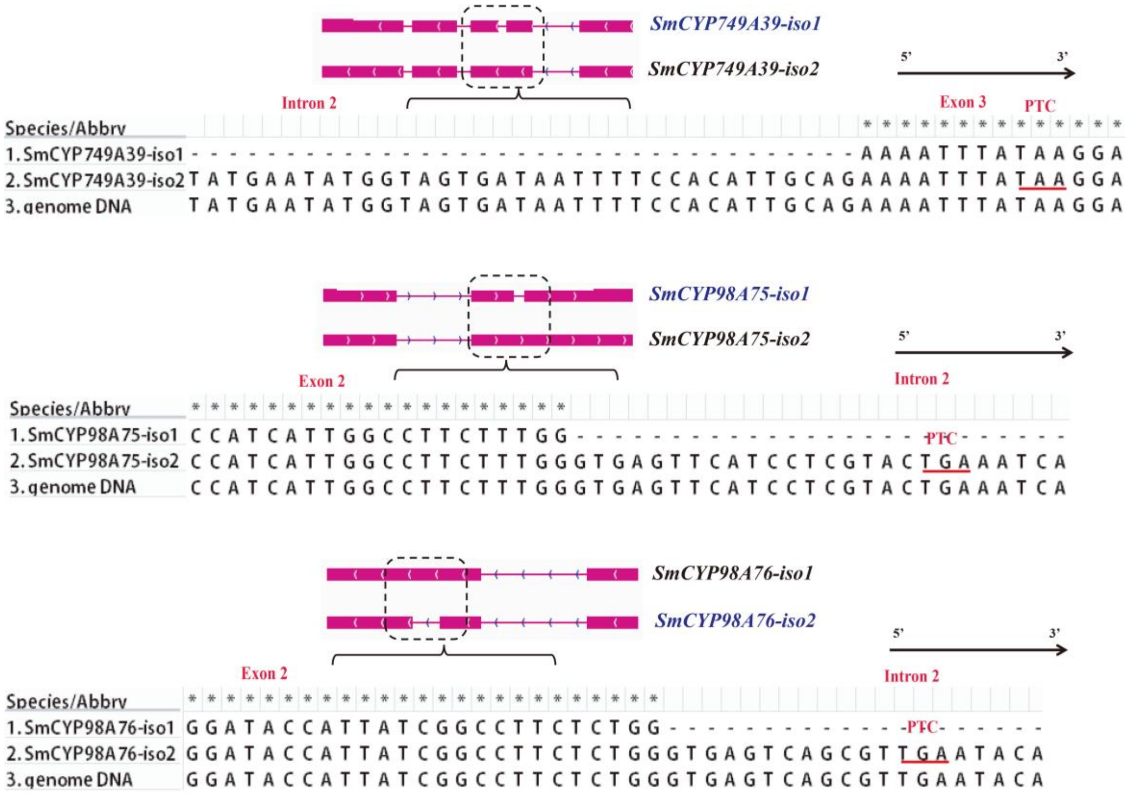

Figure S4. The alternative splicing events in candidate CYP450s related to phenolic acids biosynthesis introduce premature translation termination codons.

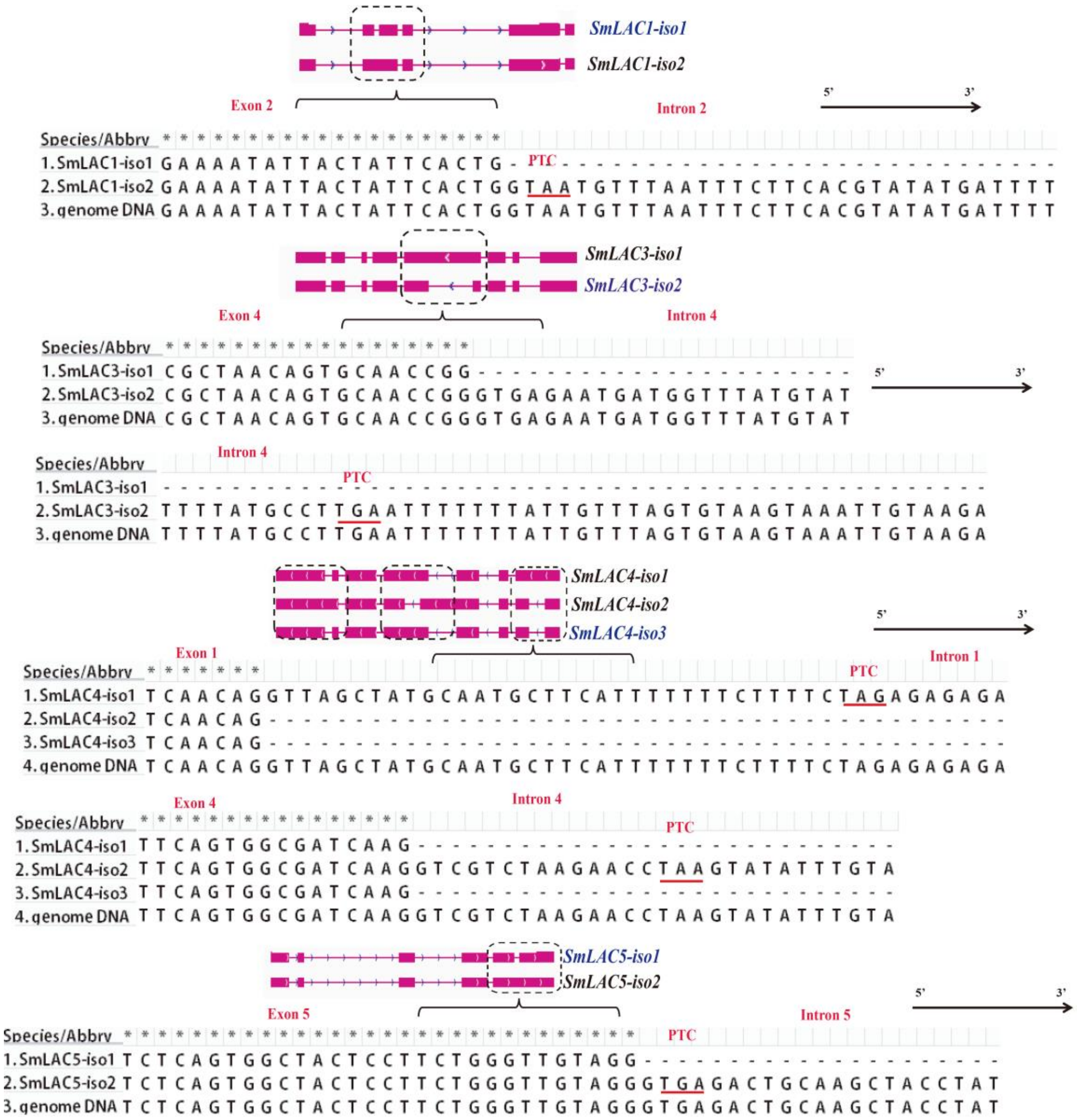

Figure S5. The alternative splicing events in candidate laccases related to phenolic acids biosynthesis introduce premature translation termination codons.

Table S1. The primers used for qPCR analysis.

**Gene_name**	**Gene_ID**	**Primer pairs (5’-3’ Forward/Reverse)**
SmCYP73A120	SMil_00000716	TCAGGCTGTGGTCAAGGAG/ACCAAGCGTTCACCAAGATC
SmCYP98A78	SMil_00028636	TAAGGAGGTGCTGAAGGAGAA/GAGAAGAGGACGACGGTACA
SmCYP749A39	SMil_00010423	GGATGGCTAAGGCAACGAAT/GTGAACATAGTCCGACGAGAG
SmCYP714C2	SMil_00023347	ACGACAAGAGGTCATTCAAGTT/GGAATACGAATGTTGCCGAATT
SmCYP707A102	SMil_00000394	CTCGTCCTCAGAGCCTTCA/GGAGAGCACTGCCACATTG
SmCYP92A73	SMil_00027803	CGGAGGAGTTCAAGAAGATGTT/CTTGCTCACCACCTTCATCC
SmCYP98A75	SMil_00026609	ATGGCTGCGATGGATGTTC/AAGTGCTGCTTGGCTCCT
SmCYP98A76	SMil_00026146	CTCCTCCACCACCTCTACTAC/AACCAAACCACACGGATATGAT
SmLAC1	SMil_00009266	TGGACAGCGATTAGGTTCAAG/GTGGAGGCAGCATCTTAGC
SmLAC2	SMil_00023004	CCTGTGCCTGATGAAGAAGTT/TTGAGCATGGAAGGTCATACAA
SmLAC3	SMil_00000484	AGGCATCTGGTCCACTTCC/ACATACACCTCGGTCACATTG
SmLAC4	SMil_00003461	AGGTCGGATGGTCTGTTGAT/GCTTGCCGTTGATGATAGGT
SmLAC5	SMil_00018228	CTCTCAGGTCCTCACCAACA/GCCGTCAGATTAGTCCTCAAG
SmActin	SMil_00015538	AGGAACCACCGATCCAGACA/GGTGCCCTGAGGTCCTGTT

Table S2. Genome-wide identification of Phenolic acid biosynthetic genes in *Salvia miltiorrhiza*.

**Gene Names**	GeneBank	Gene_ID	Periderm	Phloem	xylem	Root	Flower	Stem	MeJA-0	MeJA-12
SmPAL1	EF462460.1	SMil_00019884	127.57	217.88	295.87	274.57	361.99	273.80	703.65	1333.62
SmPAL2	GQ249111.1	SMil_00002889	2.69	0.39	6.22	77.08	110.79	239.70	3.86	1.51
SmPAL3	KF220569	SMil_00012897	48.92	82.20	63.39	208.83	221.28	182.17	314.93	317.16
SmC4H1	DQ355979.1	SMil_00000716	135.63	175.22	168.26	396.33	319.84	466.82	1147.81	1574.38
SmC4H2	KF220564	SMil_00000715	0.44	0.00	0.02	0.18	5.63	1.19	2.61	0.46
Sm4CL1	AY237163.1	SMil_00008129	1.68	1.82	2.25	6.22	12.29	16.91	7.07	9.95
Sm4CL2	AY237164.1	SMil_00021322*	23.10	3.28	11.46	114.91	67.86	278.66	19.41	11.04
Sm4CL3	KF220556	SMil_00016012	31.57	58.44	46.40	165.55	47.00	49.77	99.17	138.92
Sm4CL-like1	KF220557	SMil_00024511	61.46	74.33	75.13	857.75	45.20	372.79	24.08	60.74
Sm4CL-like2	KF220558	SMil_00004892	31.67	15.38	12.97	20.85	40.48	21.29	19.12	27.96
Sm4CL-like3	KF220559	SMil_00021404	0.04	0.01	0.00	0.05	0.53	0.00	0.10	0.03
Sm4CL-like4	KF220560	SMil_00015097	18.92	82.02	153.76	1126.21	280.54	379.51	328.31	126.69
Sm4CL-like5	KF220561	SMil_00017713*	10.65	6.87	4.97	3.96	10.94	6.74	15.24	14.02
Sm4CL-like6	KF220562	SMil_00018588	0.75	0.69	0.56	4.92	17.88	11.24	44.30	3.59
Sm4CL-like7	KF220563	SMil_00011443*	32.54	27.84	25.72	18.13	1.48	10.26	0.78	1.25
SmTAT1	DQ334606.1	SMil_00024924*	169.92	429.09	266.82	226.27	104.04	54.37	1236.21	1383.10
SmTAT2	KF220575	SMil_00002851	13.60	4.93	4.16	1.17	155.61	0.25	24.85	42.63
SmTAT3	KF220555	SMil_00020694	0.19	0.04	0.08	0.05	4.65	0.10	1.71	1.18
SmHPPR1	DQ099741.1	SMil_00002680	74.81	54.45	45.41	66.45	55.42	97.83	390.21	750.94
SmHPPR2	KF220565	SMil_00013707	3.75	3.76	4.05	3.32	6.90	11.72	8.28	2.86
SmHPPR3	KF220566	SMil_00013866	46.46	49.51	64.48	8.40	17.39	10.69	61.66	118.87
SmHPPD	EF157837.1	SMil_00015702	16.56	15.60	16.39	0.57	0.69	0.29	0.21	0.59
SmRAS	FJ906696.1	SMil_00025190	217.61	347.72	401.42	162.21	338.24	125.77	467.59	958.50

SmHCT1	KF220570	SMil_00010058	4.37	1.96	655.21	1468.09	3.19	6.75	0.00	0.00
SmHCT2	KF220571	SMil_00007200	0.00	0.19	0.05	0.37	0.07	0.47	0.00	0.00
SmHCT3	KF220572	SMil_00018429	0.00	0.09	0.00	0.12	0.00	0.00	0.00	0.00
SmHCT4	KF220573	SMil_00009296	0.25	0.64	0.00	1.90	0.00	0.34	0.00	0.00
SmHCT5	KF220574	SMil_00025059	0.06	0.30	0.02	0.56	0.57	3.82	2.69	2.76
SmCYP98A78	HQ316179.1	SMil_00028636	146.84	174.26	173.20	137.73	40.60	111.14	542.82	792.42

Table S3. Genome-wide identification of candidate CYP450s related to phenolic acid biosynthesis in *Salvia miltiorrhiza*.

**Gene Names**	GeneBank	Gene_ID	Periderm	Phloem	xylem	Root	Flower	Stem	MeJA-0	MeJA-12
SmC4H1	DQ355979.1	SMil_00000716	135.63	175.22	168.26	396.33	319.84	466.82	1147.81	1574.38
SmCYP98A78	HQ316179.1	SMil_00028636	146.84	174.26	173.20	137.73	40.60	111.14	542.82	792.42
SmCYP749A39		SMil_00010423*	102.38	116.57	123.85	188.14	62.74	81.20	20.95	31.22
SmCYP714C2		SMil_00023347	12.71	33.86	29.34	37.31	0.23	0.17	0.00	0.00
SmCYP707A102		SMil_00000394	74.41	109.56	94.65	333.54	108.59	56.29	3.54	5.08
SmCYP92A73		SMil_00027803	42.76	82.11	63.49	15.19	7.00	64.43	11.05	15.06
SmCYP98A75		SMil_00026609*	70.83	110.28	94.86	154.78	88.54	158.57	398.94	511.12
SmCYP98A76		SMil_00026146*	21.82	25.30	28.13	73.76	47.81	173.05	63.90	69.37

Table S4. Genome-wide identification of candidate laccases related to phenolic acid biosynthesis in *Salvia miltiorrhiza*.

Gene_name	Laccase	Periderm	Phloem	xylem	Flower	Stem	Root	MeJA0	MeJA12
SmLAC1	SMil_00009266*	29.30	33.65	33.38	8.29	8.84	26.20	0.85	1.73
SmLAC2	SMil_00023004	60.51	111.02	77.64	817.39	125.65	119.69	150.11	312.70
SmLAC3	SMil_00000484*	3.09	17.14	15.29	23.68	28.18	103.48	15.38	10.32
SmLAC4	SMil_00003461*	2.88	29.76	16.38	190.32	201.56	271.59	10.51	11.10
SmLAC5	SMil_00018228*	2.26	5.82	6.94	103.41	121.90	16.15	42.01	99.07

Table S5. The expression patterns of differentially spliced isoforms of enzymatic gens from phenolic acid biosynthesis in *Salvia miltiorrhiza*.

Gene name	Gene isoforms	FPKM
Sm4CL2	Sm4CL2-iso1	10.61
	Sm4CL2-iso2	42.02
Sm4CL-like5	Sm4CL-like5-iso1	1.57
	Sm4CL-like5-iso2	17.53
Sm4CL-like7	Sm4CL-like7-iso1	25.57
	Sm4CL-like7-iso2	22.70
	Sm4CL-like7-iso3	35.43
SmTAT	SmTAT1-iso1	38.42
	SmTAT1-iso2	30.12
	SmTAT1-iso3	253.99
SmCYP749A39	SmCYP749A39-iso1	93.12
	SmCYP749A39-iso2	11.26
SmCYP98A75	SmCYP98A75-iso1	179.27
	SmCYP98A75-iso2	18.95
SmCYP98A76	SmCYP98A76-iso1	4.96
	SmCYP98A76-iso2	48.52
SmLAC1	SmLAC1-iso1	47.78
	SmLAC1-iso2	6.15
SmLAC3	SmLAC3-iso1	1.09
	SmLAC3-iso2	6.33
SmLAC4	SmLAC4-iso1	7.95
	SmLAC4-iso2	6.27
	SmLAC4-iso3	36.34
SmLAC5	SmLAC5-iso1	5.40
	SmLAC5-iso2	1.33
